# Phosphoglycerate kinase: structural aspects and functions, with special emphasis on the enzyme from Kinetoplastea

**DOI:** 10.1098/rsob.200302

**Published:** 2020-11-25

**Authors:** Maura Rojas-Pirela, Diego Andrade-Alviárez, Verónica Rojas, Ulrike Kemmerling, Ana J. Cáceres, Paul A. Michels, Juan Luis Concepción, Wilfredo Quiñones

**Affiliations:** 1 Instituto de Biología, Facultad de Ciencias, Pontificia Universidad Católica de Valparaiso, Valparaiso 2373223, Chile; 2 Laboratorio de Enzimología de Parásitos, Departamento de Biología, Facultad de Ciencias, Universidad de Los Andes, Mérida 5101, Venezuela; 3 Instituto de Ciencias Biomédicas, Universidad de Chile, Facultad de Medicina, Santiago de Chile 8380453, Santigo de Chile; 4 Centre for Immunity, Infection and Evolution, The King's Buildings, Edinburgh EH9 3FL, UK; 5 Centre for Translational and Chemical Biology, School of Biological Sciences, The University of Edinburgh, The King's Buildings, Edinburgh EH9 3FL, UK

**Keywords:** phosphoglycerate kinase, domains, moonlighting protein, protists, Trypanosoma, metabolism

## Abstract

Phosphoglycerate kinase (PGK) is a glycolytic enzyme that is well conserved among the three domains of life. PGK is usually a monomeric enzyme of about 45 kDa that catalyses one of the two ATP-producing reactions in the glycolytic pathway, through the conversion of 1,3-bisphosphoglycerate (1,3BPGA) to 3-phosphoglycerate (3PGA). It also participates in gluconeogenesis, catalysing the opposite reaction to produce 1,3BPGA and ADP. Like most other glycolytic enzymes, PGK has also been catalogued as a moonlighting protein, due to its involvement in different functions not associated with energy metabolism, which include pathogenesis, interaction with nucleic acids, tumorigenesis progression, cell death and viral replication. In this review, we have highlighted the overall aspects of this enzyme, such as its structure, reaction kinetics, activity regulation and possible moonlighting functions in different protistan organisms, especially both free-living and parasitic Kinetoplastea. Our analysis of the genomes of different kinetoplastids revealed the presence of open-reading frames (ORFs) for multiple PGK isoforms in several species. Some of these ORFs code for unusually large PGKs. The products appear to contain additional structural domains fused to the PGK domain. A striking aspect is that some of these PGK isoforms are predicted to be catalytically inactive enzymes or ‘dead’ enzymes. The roles of PGKs in kinetoplastid parasites are analysed, and the apparent significance of the PGK gene duplication that gave rise to the different isoforms and their expression in *Trypanosoma cruzi* is discussed.

## 1. Introduction

Metabolism is a fundamental process in living organisms, consisting of a network of biochemical reactions catalysed and regulated by enzymes. The activities of these enzymes support a wide variety of processes such as cell growth and proliferation, synthesis of cellular components and generation of forms of energy to sustain them. Some enzymes such as kinases are critical in metabolism and other cellular processes necessary for homeostasis and cell survival, although when homeostasis breaks down they can also be involved in the development of cancer and other diseases [1]. In many eukaryotes, kinases form one of the largest gene families, representing approximately 2% of all genes [2]. In parasites of clinical interest such as *Trypanosoma brucei*, *Trypanosoma cruzi* and *Leishmania* spp*.*, which in humans cause African trypanosomiasis, Chagas disease and leishmaniasis, respectively, protein kinases (PKs) represent approximately 2% of the proteins encoded in each genome, as they do in other eukaryotic organism [3]. The significant presence of PKs encoded in these parasites suggests a key role of these enzymes in their biology [4]. These parasites belong to the Kinetoplastea, a group of flagellated protists comprising members that parasitize many plant and animal species, and in humans are responsible for diseases with serious public health threats and socioeconomic effects [5].

**Figure 1.**
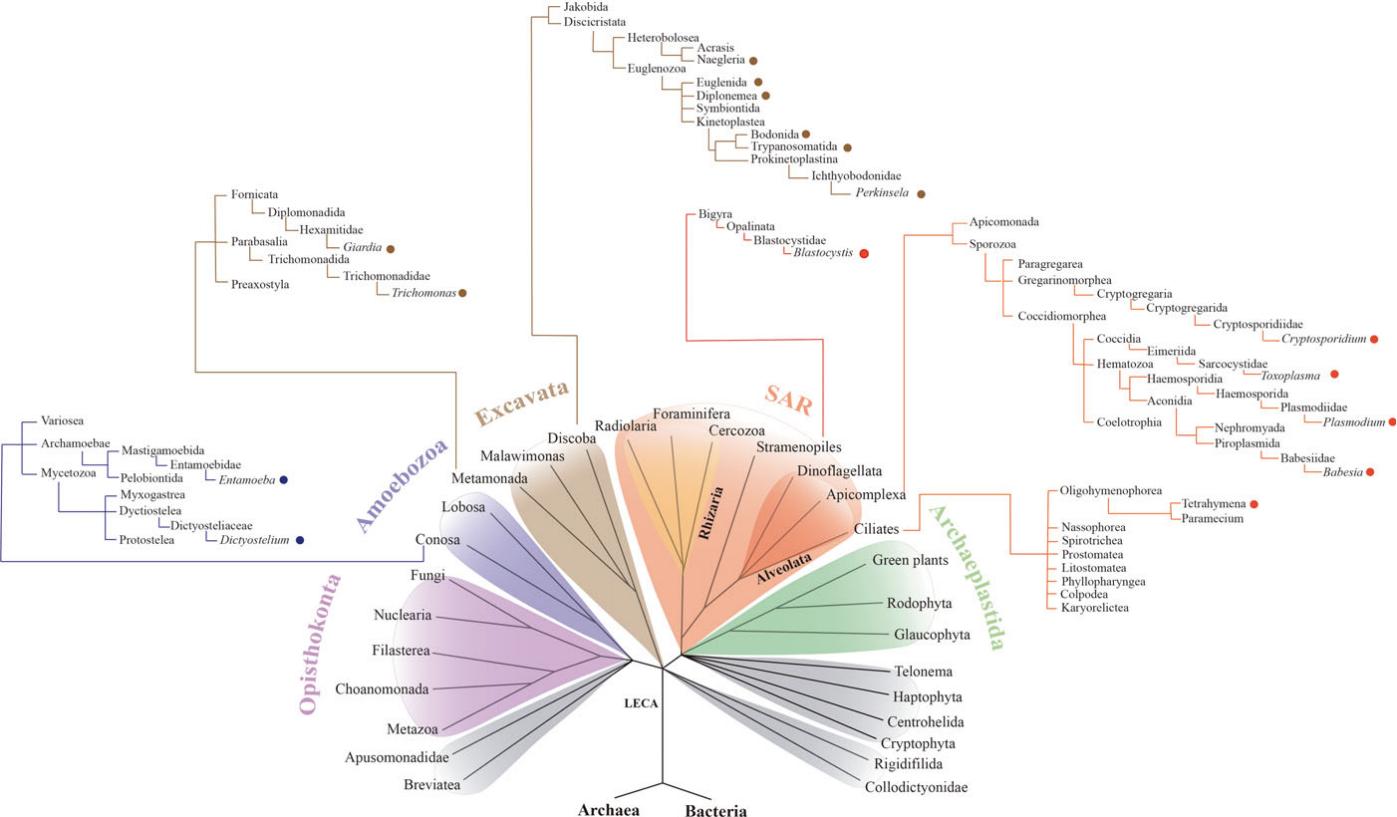
Phylogenetic relationships of the eukaryotes. LECA is the ‘last eukaryotic common ancestor’ from which different eukaryotic supergroups evolved [6]. The main protistan organisms discussed in this paper are indicated by coloured dots.

Protists embrace many diverse unicellular eukaryotes, which in modern taxonomy are understood to be paraphyletic (i.e. not forming a single clade) and exclude unicellular fungi [6]. Thus, protists are found in all so-called supergroups, the recognized major eukaryotic subdivisions: Amorphea (Amoebozoa, Opisthokonta), Archaeplastidia, SAR (which includes Stramenopila, Alveolata and the relatively little studied Rhizaria) and Excavata [6]. Figure 1 shows the phylogenetic position of the Kinetoplastea (within the Excavata) with respect to mammals and fungi (within the Opisthokonta) and to various other parasitic and free-living protistan genera which figure in this review and belong to various supergroups: *Dictyostelium, Entamoeba, Trichomonas, Giardia, Blastocystis, Plasmodium, Toxoplasma, Naegleria, Euglena* and *Diplonema*.

**Figure 2.**
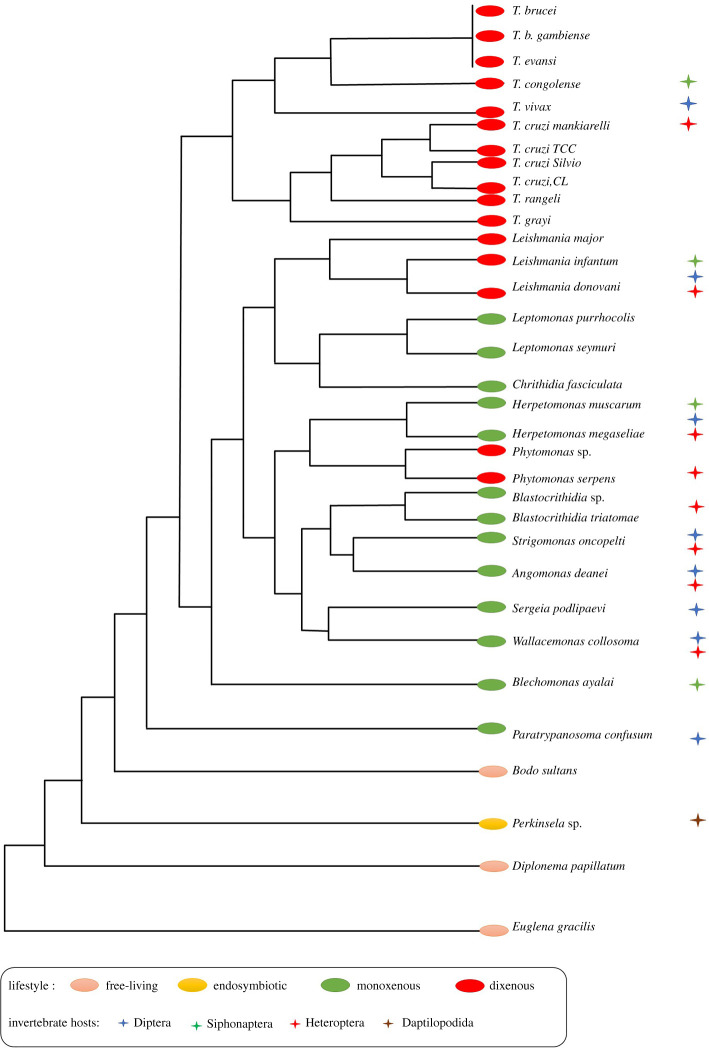
Evolutionary relationships among Kinetoplastea. Outgroups for the construction of the tree (based on small subunit ribosomal RNA sequences) are *D. papillatum* and *E. gracilis*, which, together with the Kinetoplastea, belong to the clade Euglenozoa (figure 1). Figure based on [16].

Some of the many PKs identified in kinetoplastids have been shown to be involved in different cellular processes, including transducing signals from the surface of the cell to the nucleus [7–11]. Kinases are classified according to their amino acid sequence similarity and are grouped into subfamilies which share general functional properties [12]. In recent studies, some metabolic enzymes have been included in the protein kinome [13–15]. Attributed to this group of metabolic PKs are also some proteins related to the enzyme phosphoglycerate kinase (PGK) with demonstrated and/or predicted non-metabolic function, thus so-called moonlighting PGKs. Structural and functional aspects of the PGKs from kinetoplastid organisms will be discussed in detail in this review. Figure 2 highlights these organisms which include both parasitic and free-living organisms, the endosymbiotic kinetoplastid *Perkinsela* sp*.,* as well as the free-living *Diplonema papillatum* and *Euglena gracilis,* belonging to separate taxonomic lineages which, together with the Kinetoplastea, are grouped within the Euglenozoa.

## 2. Phosphoglycerate kinase

PGK (E.C. 2.7.2.3), also known as ATP:3-phospho-d-glycerate 1-phosphotransferase, is considered an essential enzyme for many organisms. This enzyme is mainly associated with a metabolic function, when it catalyses the phospho transfer between two intermediates of carbohydrate metabolism [17]. In glycolysis, this enzyme catalyses the transfer of a phospho group from 1,3-bisphosphoglycerate (1,3BPGA) to ADP to produce 3-phosphoglycerate (3PGA) and ATP in which the high energy released from the carbon substrate is invested. The opposite reaction, in the direction of 1,3BPGA and ADP formation, takes place during gluconeogenesis [18,19]. The forward reaction is one of the two ATP-generating steps in the glycolytic pathway, hence its importance in the metabolism of many organisms, such as protists, both free-living and parasitic ones, the latter including *Plasmodium falciparum* [20], *Entamoeba histolytica* [21] and kinetoplastids of clinical interests, such as *T. brucei*, *T. cruzi* and *Leishmania* spp. [4,22–28].

The first attempts to elucidate the functional characteristics of this enzyme occurred in the 1940s, through research performed by Bücher [18,29] who isolated and crystallized a PGK from yeast extracts. After these initial studies, the analysis focused on PGK started to increase markedly, leading to the elucidation of the first low-resolution structures of PGKs from horse muscle [30] and yeast [31]. Later, the discovery of the autosomal *pgk-2* gene in human and mouse testis cells marked a great impulse in the research history of this protein [32]. Currently, the information available about PGK in the NCBI database is considerably extensive and the crystal structural data present in the RSCB Protein Data Bank comprise PGKs from a broad range of organisms, with both structures of the protein in the apo form and with bound substrates, products or molecules that mimic substrates, as well as in open and closed protein conformations [33] (table 1).

**Table 1.** A sample of PGKs available at the Protein Data Bank. 3PGA, 3-phosphoglyceric acid; AMP, adenosine 5′-monophosphate; ADP, adenosine 5'-diphosphate; ATP, adenosine 5'-triphosphate; BTB, 2-[Bis-(2-hydroxy-ethyl)-amino]-2-hydroxymethyl-propane-1,3-diol; Cl^−^, chloride ion; K^+^, potassium ion; Mg^2+^, magnesium ion; FMT, formic acid; PGE, triethylene glycol; TZ, [4-(4-amino-6,7-dimethoxyquinazolin-2-yl)piperazin-1-yl][(2R)-tetrahydrofuran-2-yl]methanone; SO_4_^2−^, sulfate ion; ANP, phosphoaminophosphonic acid-adenylate ester; GOL, glycerol; MPD (4S)-2-methyl-2,4-pentanediol; AMP-PNP, adenylyl-imidodiphosphate; NA, not available.

organism	domain	enzyme	stoichiometry	conformation	ligands	PDB	reference
*Pyrococcus horikoshii*	archaea	PGK	homodimer		3PGA, GOL, MPD, Cl^−^	2CUN	Mizutano & Kunishima (NA)
*Francisella tularensis*	bacteria	PGK	monomer	open	ADP	4FEY	Brunzelle *et al*. (NA)
*Thermotoga maritima*	bacteria	PGK	monomer	closed	3PGA, ANP, Mg	1VPE	[34]
*Staphylococcus aureus*	bacteria	PGK	monomer	—	—	4DG5	Roychowdhury *et al*. (NA)
*Streptococcus pneumoniae*	bacteria	PGK	monomer		3PGA, AMP-PNP	3ZLB	[35]
*Campylobacter jejuni*	bacteria	PGK	monomer	open	K^+^, SO_4_, FMT, PGE	3Q3 V	[36]
*Thermus caldophilus*	bacteria	PGK	monomer	open	—	2IE8	[37]
*Bacillus anthracis*	bacteria	PGK	monomer	open	Cl^−^, Mg^2+^, BTB	3UWD	[36]
*Thermus thermophilus*	bacteria	PGK	monomer	—	Na^+^, GOL	1V6S	Mizutani *et al*. (NA)
*Escherichia coli*	bacteria	PGK	monomer	—	Ca^+^	1ZMR	[38]
*Pseudomonas* sp*. 'TAC II 18*	bacteria	PGK	monomer	open	3PGA	6HXE	[39]
	bacteria	PGK	monomer	open	—	6I06	[39]
*Acinetobacter baumannii*	bacteria	PGK	monomer	—	—	5BT8	Fairman *et al*. (NA)
*Coxiella burnetii*	bacteria	PGK	monomer	—	ADP, Mg^2+^	4NG4	[40]
*Geobacillus stearothermophilus*	bacteria	PGK	monomer	open	ADP, Mg^2+^	1PHP	[41]
*Homo sapiens*	eukarya	PGK-1	monomer	open	3PGA, ADP	2XE7	[42]
*Saccharomyces cerevisiae*	eukarya	PGK	monomer	closed	3PGA, ATP, Mg^2+^	3PGK	[43]
*Mus musculus*	eukarya	PGK-1	monomer	open/closed	3PGA, ATP, TZ	4O3F	[44]
		PGK-2	monomer	open/closed	3PGA, ATP	2PAA	[45]
*Sus scrofa*	eukarya	PGK	monomer	open/closed	3PGA, AMP, Mg^2+^	1HDI	[46]
*Plasmodium falciparum*	eukarya	PGK	monomer	open	SO_4_^2−^	3OZ7	[47]
*Plasmodium vivax*	eukarya	PGK	monomer	—	K^+^, Br^−^	6Y3A	Bilsland *et al*. (NA)
*Trypanosoma brucei*	eukarya	PGK	monomer	closed	3PGA, ADP, Mg^2+^	13PK	[48]
*Equus caballus*	eukarya	PGK	monomer	open	—	2PGK	[49]

PGK is a highly conserved enzyme that has been found in members of each of the three domains of life: Bacteria, Archaea and Eukarya [50–52]. In-depth studies of the structure, function and general properties of PGKs have often been focused on seeking chemotherapeutic agents since this glycolytic enzyme has been considered a target for treatment of some diseases in humans [53]. In addition to its role in glycolysis and gluconeogenesis, and a related metabolic role in the carbon-fixation reactions in autotrophic organisms [54,55], other functions have been attributed to this enzyme. Its non-canonical functions are related to processes such as DNA replication and repair [56], angiogenesis and tumour growth [57], plasminogen binding [58], cell invasion [59], being a constituent of a flagellar axoneme [60] and viral replication [61], among others, which will be highlighted in the following sections of this paper.

In this review, several aspects related to the structure and function of PGKs in various protists will be discussed, with emphasis on kinetoplastids. This analysis could serve as a prelude to consider additional functions of this enzyme in the biology of trypanosomatid parasites, since most PGK studies of these parasites so far focused mainly on its glycolytic function.

## 3. Structural aspects of phosphoglycerate kinase

**Figure 3.**
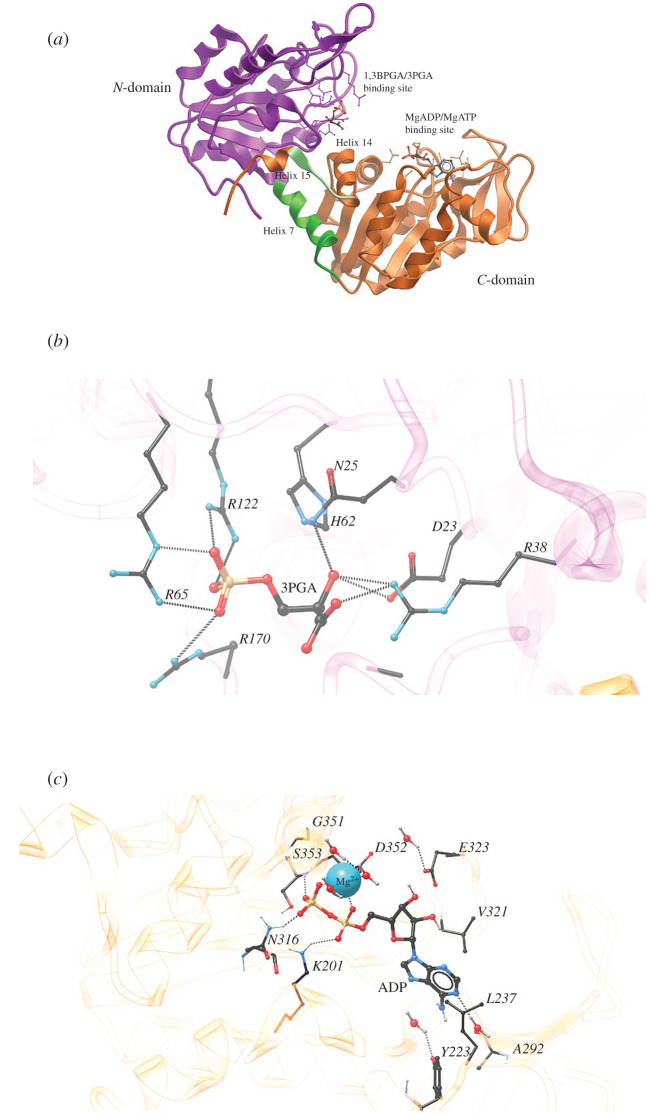
Three-dimensional structure of phosphoglycerate kinase. (*a*) Ribbon representation of the overall structure of pig muscle PGK (PDB: 1HDI). In colour is highlighted the N-domain (violet); helix 7 or interdomain helix (green); the link between helix 14 and 15; amino acids 404–408 (light green), and the C-domain (orange). The substrate binding sites for 1,3BPGA/3PGA and MgADP/MgATP are indicated in both the N- and C-domain, respectively. The pig muscle ternary complex shown here exhibits an open conformation in comparison with the ternary complex of other PGK structures [46]. (*b*) Substrate 3PGA binding site at the N-terminal domain of pig muscle PGK. (*c*) The MgADP/MgATP binding site of *B. stearothermophilus* (bacterium later renamed to *Geobacillus stearothermophilus*) for the ligand MgADP (PDB: 1PHP). In both (*b*,*c*), interactions between amino acid residues and substrate through hydrogen bonds (dashed lines) are shown. Atom colour code: black (carbon); white (hydrogen); red (oxygen); blue (nitrogen), orange (phosphorus). Ion colour code: cyan (magnesium, Mg^2+^).

PGK enzymes have been isolated from a wide variety of organisms. It is a typical hinge-bending monomeric enzyme with a molecular weight of approximately 45 kDa [62] (table 2). The enzyme is composed of a single folded polypeptide chain that forms two domains of almost equal size, which are separated by a deep cleft, and linked by two α-helices (α-helices 7 and 14) endowing it with its characteristic bilobed structure [73]. Both the N- and C-terminal domain contain a typical Rossmann fold with a core of six parallel strands of β-sheets surrounded by α-helices. The β-turns and irregular structure segments connect both the β-sheets and α-helices [19,36,43]. Both domains are involved in substrate binding, with the N-terminal domain binding 3PGA or 1,3BPGA, whereas the C-terminal domain binds MgADP or MgATP (figure 3*a*).

**Table 2.** General physico-chemical and kinetic parameters of some characterized PGKs. ND, not determined; NS, not specified.

organism	domain	enzyme	stoichiometry	enzyme type	MW (kDa)	optimal pH	pI	ATP (mM)	3PGA (mM)	reference
*Geobacillus stearothermophilus*	bacteria	PGK	monomer	wild-type	42	5.5–8.5	4.9	2.9	2.2	[63]
*Corynebacterium glutamicum*	bacteria	PGK	homodimer	recombinant	104	7–7.4	ND	0.11	0.26	[64]
*Pseudomonas* sp*. 'TAC II 18'*	bacteria	PGK	monomer	wild-type	40.1	8	4.9	0.21	0.53	[65]
				native	40.1	ND	ND	0.24	0.48	[65]
				His-tagged	ND	ND	ND	0.38	0.89	[65]
*Synechocystis* sp*.*	bacteria	PGK	monomer	recombinant	ND	ND	ND	0.19	0.18	[55]
*Thermus thermophilus*	bacteria	PGK	monomer	wild-type	44.6	6–8.5	5	0.28	1.79	[66]
*Zymomonas mobilis*	bacteria	PGK	monomer	wild-type	44	ND	ND	1.1	1.5	[67]
*Entamoeba histolytica^b^*	eukarya	PGK	ND	wild-type	ND	ND	ND	5	NS	[21]
			monomer	recombinant	48.4	5–7	ND	1.439	0.547	[21]
*Phaeodactylum tricornutum*	eukarya	PGKase-1	monomer	recombinant	45	ND	ND	0.89	1.55	[54]
*Leishmania major^c^*	eukarya	PAS-PGK	monomer	recombinant	62	5.5	ND	0.118	0.661	[28]
*Leishmania mexicana mexicana*	eukarya	PGKB	monomer	recombinant	47	ND	ND	0.24	1.45	[68]
		PGKC	monomer	recombinant	53	ND	ND	0.13	2.75	[68]
*Trypanosoma rangeli*	eukarya	PGKB	monomer	wild-type	44	ND	ND	0.13	0.28	[27]
			monomer	recombinant	44	8–10	ND	0.5	0.71	[27]
*Trypanosoma brucei*	eukarya	PGKB (cytosol)	monomer	wild-type	45	6–9	6.3	0.46	2.04	[22]
			monomer	recombinant	45	ND	ND	0.21	1.25	[69]
		PGKC (glycosome)	monomer	wild-type	47	ND	9.3	0.28	1.55	[69]
			monomer	recombinant	47	ND	ND	0.29	2.02	[69]
*Trypanosoma cruzi*	eukarya	PGKA	monomer	wild-type	56	ND	6.98^a^	0.217	0.174	[24]
			monomer	recombinant	ND	8	ND	0.236	0.850	[25]
		PGKB	monomer	wild-type	47	5.5	6.6	0.09	0.62	our unpublihed results
		PGKC	monomer	wild-type	48	ND	ND	0.10	0.192	[25]
*Spirulina geitleri*	eukarya	PGK	monomer	wild-type	ND	ND	4.8	0.48	1.5	[70]
*Spinacea oleracea* (spinach)	eukarya	PGK	monomer	wild-type	46	6.5–9.5	4.3	0.3	1.1	[70]
yeast	eukarya	PGK	monomer	wild-type	47.1	6–9.2	7.2	0.48	1.28	[71]
rabbit muscle	eukarya	PGK	monomer	wild-type	47	6–9.2	7	0.42	1.37	[71]
*Homo sapiens*	eukarya	PGK-1	monomer	wild-type	ND	ND	ND	0.12	0.22	[72]
		PGK-2	Monomer	wild-type	ND	ND	ND	0.34	0.16	[72]

^a^Predicted.

^b^Km also calculated for GTP (wild-type: 0.016 mM; recombinant: 0.151 mM).

^c^Kinetic parameters at pH 7.5 were also calculated (ATP 0.150 mM; 3PGA 0.540 mM).

Since the 1970s, numerous crystallographic studies of this metabolic enzyme have been performed and the results deposited in the Protein Data Bank (PDB) (table 1). These structural studies of PGKs from different organisms, both prokaryotes and eukaryotes, such as horse [74], pig muscle [75], *Bacillus stearothermophilus* [41], *Thermotoga marítima* [34], *T. brucei* [48], *P. falciparum* [47] and *Bacillus anthracis* [36], have demonstrated that the overall structure of this enzyme is highly conserved. However, these studies also revealed the presence of subtle differences in the PGK structure that must have evolved in response to the environments where this enzyme works. This was most clearly evidenced by crystallographic analysis of PGKs isolated from organisms that live at different temperature ranges. In response to the environmental temperature, PGK has undergone multiple specific adaptations (global and local) [34,39]. Some of these adaptations involved an alteration in the content of hydrophobic and polar amino acids in the N-terminal core. In PGK from isolates of *Pseudomonas psychrophiles*, the N-terminal core is enriched in polar amino acids, a clear difference compared with its mesophilic and thermophilic counterparts in which predominantly hydrophobic interactions take place. The presence of these polar amino acids could be responsible for protein flexibility, a property that allows the enzyme to proceed with catalysis under low-temperature conditions. Another difference observed at the N-terminal domain of the psychrophilic PGK is an unstructured portion between the sheets β5 and β6. In the same location, the mesophilic PGK has a one-turn helix while the thermophilic and hyperthermophilic PGKs have a two-turn helix [39]. It has been suggested that an increase in flexibility in psychrophilic enzymes allows a better coupling of their substrates and undergoing rapid conformational changes necessary for catalysis, at low energy cost [76]. On the other hand, some determinants of the stability of this enzyme at extremely high temperatures have been identified in hyperthermophilic organisms such as *Thermotoga maritima*. This PGK is one of the most stable enzymes; it is characterized by having a half-life of irreversible thermal inactivation of 2 h at 100°C. The stability of this enzyme is related to a drastic decrease in its flexibility. Its thermostability is attributed to loop stabilization and a depletion phenomenon, which results in a shortening of the loops, which in turn induces a stabilization of the folded state. A reduction in flexibility in protein loop regions by stabilization possibly occurs by the formation of additional hydrogen bonds, as well as shortening or elimination of loops. The stiffness of *T. maritima* PGK is further augmented by an increase in salt bridges, especially between some residues exposed on the surface of the protein [34].

### 3.1. The substrate binding regions

The location of the substrates on PGK as well as their conformation and nature of interactions with the enzyme have been elucidated by X-ray diffraction studies, through the comparison of electron density maps [19]. The 3PGA or 1,3BPGA binding site at the N-terminal domain is characterized by the presence of a ‘basic patch’ which comprises arginines and histidines that are highly conserved among PGKs. The study of the PGK from pig muscle identified three arginine residues (at positions 65, 122 and 170) which interact through hydrogen bonds with the oxygen atoms of the substrate's 3-phosphate group (figure 3*b*). Additionally, a protonated His62 residue interacts through a hydrogen bond with the oxygen in the bridge between the phosphate and carbon skeleton of 3PGA. On the other hand, amino acids Asp23 and Asn25 are responsible for forming hydrogen bonds with the 2-oxygen of 1,3BPGA, acting as the hydrogen receptor and hydrogen donor, respectively (figure 3*b*) [77].

In addition to being the binding site of 3PGA, the basic patch seems to be a site for regulation of the catalytic activity of PGK. The inhibitory or stimulatory effect of some ions on the activity of some PGKs [47,69,78] has led to the proposal that this basic patch, besides being part of the 3PGA interaction site, can be a place of regulation of the catalytic activity, through binding an inorganic anion. However, only for *P. falciparum* is there PGK structural evidence that demonstrates the binding of ions to this ‘basic patch’ [47].

As illustrated in figure 3*c* for *B. stearothermophilus*, the C-terminal domain contains the binding site of ATP or ADP through a ‘hydrophobic pocket’ present on its surface within the cleft. The structure of this pocket is highly conserved in all PGKs studied so far. The adenine ring is flanked by residues Va1321 and Leu237 [41]. The −NH_2_ group associated with the adenine ring forms weak hydrogen bonds with the protein, mainly via the carbonyl oxygen atom of Ala292; however, it also has an additional interaction through a water molecule to the hydroxyl group of Tyr223. The pentose in the nucleotide forms hydrogen bonds with Asp323 through the ribose 2′- and 3′-hydroxyl groups (figure 3*c*). This molecule adopts an unusual conformation due to the location of the adenine ring and the phosphate groups in the active site. The α- and β-phosphate groups form coordinate bonds with the Mg^2+^ which also establishes coordinate contacts with Asp352 and two water molecules. Additionally, Lys201 forms a hydrogen bond specifically with the β-phosphate, while Asn316 and Ser353 interact through hydrogen bonds with the α-phosphate group. Finally, the β-phosphate lies close to the amino end of the α-helix (helix 12 in the yeast nomenclature) to interact with the characteristic Gly349–Gly350–Gly351 motif. This motif is presumably stabilized by the α-helix dipole and via hydrogen bonding interactions with the main-chain amide H atoms of residues Gly351 and Asp352 [41]. The phosphoryl-group transition state is stabilized by the required divalent Mg^2+^. This metal ion exhibits a tetragonally distorted octahedral coordination. Axial bonds are through a single water molecule and one of the carboxylate oxygen atoms of Asp352. In equatorial bonds intervene two water molecules and two oxygen atoms of the phosphate groups, one of each of the phosphate groups α and β. Except for the interaction with the Asp, most of the coordinating atoms are at a distance between 2.27 and 2.29 Å from the Mg^2+^. The oxygen atom from the aspartate carboxylate is only at a distance of 1.96 Å from the Mg^2+^, which reflects the charged nature of this interaction [41]. It has been suggested that the presence Mg^2+^ is key to PGK activity, because this divalent ion, by forming a complex with the nucleotide's α- and β-phosphates, and thus shielding their negative charges, allows the nucleophilic attack to occur when the ligands are bound to the enzyme. This charge-stabilization phenomenon is a distinctive characteristic of phosphoryl transfer reactions [79].

The amino acid composition of the substrate-binding sites of PGKs appears to be conserved between the different organisms, including those of the kinetoplastids studied so far [4,27,51,80,81].

### 3.2. The PGK hinge-bending motion and its catalytic mechanism

In the absence of substrate, PGK exhibits an ‘open’ conformation. The simultaneous binding of both substrates, 3PGA/1,3BPGA and Mg-ADP/Mg-ATP, in the N- and C-terminal domain, respectively, induces an extensive hinge-bending motion, which leads to a ‘closed’ conformation by the domains approaching each other and so bringing their bound substrates into proximity [82]. In the case of the glycolytic reaction, this close proximity favours the nucleophilic attack by the ADP-β-phosphate oxygen atom at the 1-phosphate of 1,3BPGA. This reaction involves a change in the configuration in the γ-phosphate group, followed by a unique displacement mechanism, in which a direct transfer of the phosphoryl group between the bound substrates occurs through a charged transition state. The additional negative charge, which evolves in the SN2-reaction at the pentagonal transition state phosphate, is stabilized by the enzyme [34]. During its catalytic cycle, PGK seems to spend most of its time in a completely open conformation with short periods of closure and catalysis; this allows rapid diffusion of substrates and products into and out of the binding sites. It is important to note that PGK has a propensity for the open form, and only the simultaneous binding of both substrates affects this conformation [42,83].

Studies of the human PGK have revealed that the binding of 3PGA induces the alignment and approach of two residues, Arg65 and Arg170 (both present in the N-terminal domain), allowing them to form essential salt bridges with a reoriented Asp218 (present in the C-terminal domain) only in the closed conformation. These salt bridges are the main contributors to the stabilization of the catalytically active closed conformation. In turn, the binding of ADP promotes the displacement of catalytically essential residues in a ‘catalytic loop’, extending helix-8 and prepares the C-terminal domain to stabilize the closed conformation. The presence of a hydrophobic region or hydrophobic ‘patch’ encrypted in the enzyme seems to favour the open conformation. This region, comprising several residues in the hinge helix-7 as well as other residues in the protein's core, becomes exposed during domain closure, and is responsible for returning the enzyme to the open conformation by acting as a ‘spring-loaded’ release mechanism and makes the open conformation the thermodynamically most stable one. On the other hand, ionic interactions act to keep the conformation closed to allow catalysis [42].

The catalysis-associated mechanism of hinge movement, documented for human PGK, is very similar to that reported for PGKs from other organisms, including the PGK isoenzyme of *T. brucei* that is located in peroxisome-related organelles called glycosomes which, in kinetoplastids, contain the major part of the glycolytic pathway [34,36,48]. Although the *T. brucei* PGK is a hinge flexion enzyme, its catalysis activation mechanism is very peculiar, since the effects induced by the binding of the two substrates are synergistically combined to induce important conformational changes in the enzyme [48].

## 4. PGK functional forms

With regard to its structural organization, PGK is generally monomeric [62], except in some extremophilic microorganisms where it adopts a dimeric or tetrameric conformation [51,52], while in others, it has been observed as a bifunctional enzyme [52,84] (table 3). In Archaea such as *Pyrococcus woesei* and *Methanothermus fervidus*, the PGKs are homodimers in their native state, formed by monomers of approximately 46 kDa [51]. In other archaean organisms such as *Sulfolobus solfataricus*, the PGK is particularly unusual, since it consists of a tetramer of four identical subunits, each of them with an approximate molecular weight of 45 kDa, like the monomeric PGK form found in almost all other organisms [126].

**Table 3.** PGK enzymes and PGK-related proteins identified by genomic analyses in different protists. Sequences of different PGKs were retrieved from the following databases: Tetrahymena Genome Database (TGD) [85], National Center for Biotechnology Information (NCBI) [86], The UniProt Consortium (UniProt) [87], Database for *Dictyostelium discoideum* (DictyBase) [88], Ensembl Protists, The Amoeba Genomics Resource (AmoebaDB) [89], The Giardia Genomics Resource (GiardiaDB) [90], The Trichomonas Genomics Resource (TrichDB) [90], The Toxoplasma Genomics Resource (ToxoDB) [91], The Plasmodium Genomics Resource (PlasmoDB) [92], The Cryptosporidium Genomics Resource (CryptoDB) [93] and The Kinetoplastid Genomic Resource (TriTrypDB) [94]. Alignments and determination of identity percentages reported in the text were performed using *Clustal Omega* and *Muscle* (EMBL-EBI) [95]; query sequences used were Tb927.1.710, Tb927.1.700, Tb927.1.720, Tb927.11.2380, TcCLB.511419.40 and TcCLB.511419.50. Identification of domains and motifs present in PGK sequences was done using the following bioinformatics servers: *PAS domain*: Protein Blast (NCBI) [96], InterProScan (EMBL-EBI) [97] and Simple Modular Architecture Research Tool (SMART) [98]; *e*-values ranges between 1 e^−6^ and 8.9 e^−9^. *CNB domain*: NCBI, EMBL-EBI and SMART; *e*-values ranges between 2.6 e^−6^ and 4 e^−36^. *HTH (Helix–Turn–Helix)* motif: ExPaSy [99], GYM [100] and iDNA-Prot [101]; *e*-values range between −0.08 and 1.45. For the PGK of *T. congolense*, the HTH domain was identified with a score of 29. *Membrane helix*: SMART [98], Predicting transmembrane protein topology with a hidden Markov model (TMHMM prediction) [102] and Phobius [103]; *e*-values ranges between 0.8 and 1. A specific *e*-value for each identified protein is given in electronic supplementary material, table SIII. The subcellular localization prediction of identified PGKs was based on either recognition of a PTS1 consensus sequence for glycosomal localization as described by Acosta *et al*. [104] or using the following servers: DeepLoc [105], WoLF PSORT [106] and Cell-Ploc [107]. Subcellular localization symbology: *, predicted location through bioinformatics tools; ♦, established localization through experimental studies; **◊**, established localization through experimental studies in a non-sequenced isolate; ▴. based on consensus sequence proposed in other studies [104]. Localization symbology: Cytosol, Cytosol; Mitoch, Mitochondrion; P. memb, Plasma membrane; Lys, lysosome; nucl, Nucleus. Domain symbology: MYCBP-associated protein domain 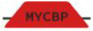, calmodulin-binding domain 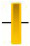, AAA-ATPases domain (Walker A/B) 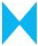, PAS domain 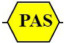, transmembrane domains 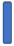/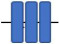, PGK domain 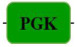, PGK domain having lost residues involved in binding substrate (3PGA) 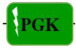, PGK domain having lost residues involved in binding ATP 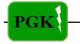, PGK domain having lost residues involved in binding substrate (3PGA) and residues binding ATP 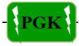, triosephosphate isomerase domain 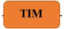, HTH domain 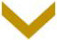, cyclic nucleotide-binding domain 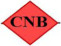.

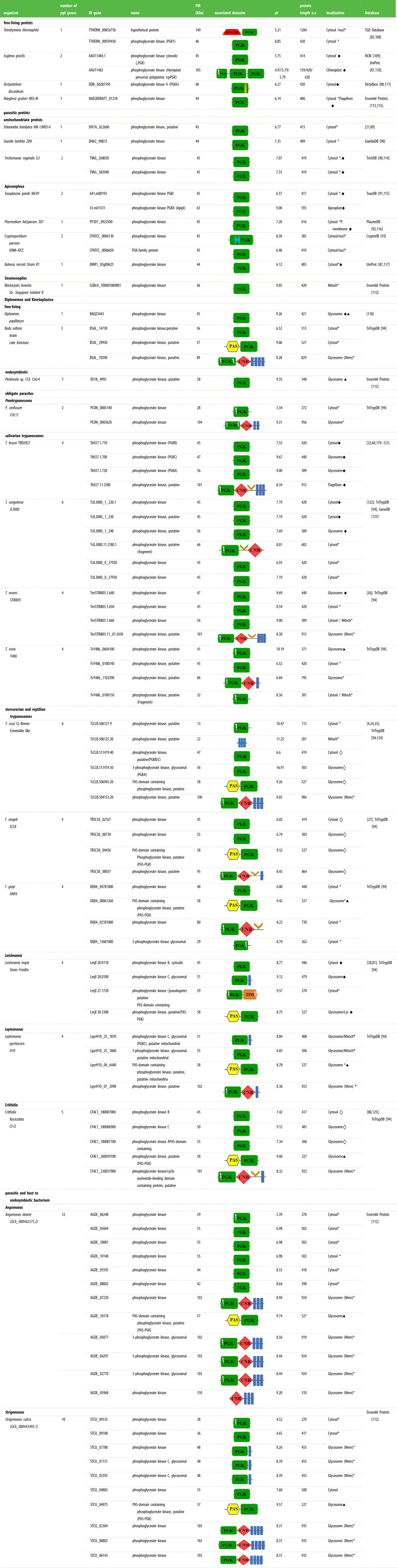

The structure of the enzyme may not only vary in the number of PGK subunits that catalyse the glycolytic reaction, but also in the presence of additional domains with other catalytic activity than PGK, as the enzymes that have been identified in some extremophiles [84]. In the thermophilic bacterium *T. maritima*, the existence of two functional forms of this enzyme has been documented, a monomeric PGK and a PGK–triose-phosphate isomerase (TIM) bifunctional complex. In this bifunctional enzyme, both a PGK domain and a TIM domain are covalently associated into a single structure. This 70 kDa PGK-TIM fusion protein is encoded by the *fus* gene. Similar to some other archaeal PGKs, this fusion protein forms tetrameric complexes [84]. Although the formation of macromolecular complexes of PGK is not a specific feature of Archaea, it has been suggested that this may be related to thermo-adaptation. Probably, dimerization contributes to greater stability of the protein by favouring hydrophobic interactions through subunit contacts and by reducing the surface area exposed to the solvent [51,127].

When analysing the *Leishmania* genome, something similar to what is observed in these extremophilic organisms was found: an open-reading frame (ORF) that showed indications for a fusion of fragments of PGK- and TIM-related genes (table 3). This will be further discussed in §8 of this review. Furthermore, proteins with additional regulatory domains associated with the PGK structure have been identified in several organisms including kinetoplastids as documented later in this review and various recent publications. The presence of some of these regulatory modules, such as the PAS-domain (Per-ARNT-Sim), calmodulin-binding domain (CaMBD), cyclic nucleotide binding domains (CNB) and transmembrane domains (TMD), possibly endow the PGK with non-canonical functions (table 3) [4,28,128]. An additional discussion of the possible implications of the presence of these regulatory domains in isoenzyme function will be discussed in the following sections.

On the other hand, in *Escherichia coli*, the PGK has been catalogued as an enzyme that is part of a complexome, associated with proteins involved in glycolysis and stress response [129]. The PGK is a component of the GmpI (phosphoglyceromutase)–Pgk–AhpC (alkyl hydroperoxide reductase) (GmpI–Pgk–AhpC) complex. The formation of such complexes could indicate some linkage of intermediate metabolism with the response to stress conditions. Furthermore, in pancreatic β cells exposed to endoplasmic reticulum (ER) stress, the integrated stress response is coupled to metabolic alternations triggered by sulfhydration of key enzymes in intermediary metabolism [130]. Additionally, among archaeal members, including extreme halophiles and some methanogens, PGK appears as a couple with glyceraldehyde-3-phosphate dehydrogenase (GAPDH/PGK) that is functionally active in the glycolytic direction, while the same coupled enzymes operate in the opposite direction (gluconeogenis) in anaerobic and hyperthermophilic Archaea [131]. A GAPDH/PGK interaction has also been reported for human erythrocytes as a pH-dependent phenomenon. This specific protein–protein interaction between GAPDH and PGK may play a role in determining the fate of 1,3BPGA produced in the GAPDH-catalysed reaction [132].

## 5. PGK activity regulation

### 5.1. Regulation by ions

PGK activity is influenced by the presence of mono- and divalent ions [51,63,64,133]. Some ions, including Ni^2+^, Co^2+^, Mn^2+^, Cd^2+^, Ca^2+^ or Zn^2+^, could stimulate or inhibit its activity. Mg^2+^ ions are necessary during catalysis by this enzyme, as reported for different PGKs studied so far (see §3.1) [63,64,134]. In *Corynebacterium glutamicum*, Zn^2+^ ions strongly inhibit the PGK activity (Ki = 0.45 mM) [64]. However, such inhibition should not be considered as a generality, since other PGKs are activated in the presence of metal ions other than Mg^2+^ [63,133]. Kinetic studies of *B. stearothermophilus* PGK have shown that Mg^2+^ and Mn^2+^ have the same contribution to its activity, while in the presence of other cations such as Co^2+^ and Ca^2+^, the enzyme showed only 58% and 15% activity, respectively [63]. On the other hand, the PGK present in pea seed tissue is apparently activated in the presence of Mn^2+^ and Co^2+^, ions that appear to be as effective as Mg^2+^ at equivalent concentrations. On the contrary, Ca^2+^ and Fe^3+^ induce a decrease of 52 and 30% of enzymatic activity, respectively. The activity of this PGK is completely inhibited by ions such as Cu^2+^, Zn^2+^ and Hg^2+^ [133]. As in other kinases, such as pyruvate kinase (PYK), divalent cations form metal–nucleotide complexes that become the substrate of the catalysed chemical reaction and lead to the activation of the enzyme (see §3.1) [135]. With regard to monovalent ions such as Na^+^ and K^+^, the influence of these ions on the activation of the enzyme can vary, even for PGKs from organisms belonging to the same kingdom. In the case of *C. glutamicum* PGK, the presence of these ions significantly increases the activity of this enzyme [64], while in *Thermus thermophilus*, PGK is slightly inhibited by Na^+^ [66]. On the other hand, in a hyperthermophilic organism such as *M. fervidus*, where PGK is a homodimeric enzyme, the activity increases in the presence of K^+^ ions. In this organism, the activity of this enzyme appears to be affected differently by Na^+^ and K^+^ salts. Apparently, there are several non-cooperative binding sites with different specificity and affinity for these ions. Hill coefficient analysis suggested that the enzyme could have at least six cooperative binding sites per dimer for K^+^ ions [51]. In the psychrophilic *Pseudomonas* sp., PGK activation is influenced by sulfate ions in a concentration-dependent manner. At lower concentrations (below 20 mM), an activation of the enzyme occurs, while at higher concentrations (50 mM), it induces a significant inhibition of enzyme activity [65]. Studies with some protists revealed similar results to those found in kinetic studies of prokaryotic PGKs. In *T. brucei*, the cytosolic and glycosomal PGK isoenzymes (recombinant and native) are negatively affected by sodium salts (NaCl and NaSO_4_) [69]. The activity of the corresponding PGK isoenzymes of *T. cruzi* appears also to be negatively affected by NaCl (at concentrations above 150 mM). In the case of *T. cruzi*, this inhibition is probably due to reduction in the solvation capacity that leads to the formation of oligomeric forms other than monomers and loss of PGK activity (W. Q. *et al*. 2006, unpublished results). It is important to note that, contrary to what has been observed in trypanosomatids, *P. falciparum* PGK is stimulated by high concentrations of Na^+^ and K^+^ salts (up to 200 mM). In addition, it has been proposed that the basic patch in the N-terminal domain of this enzyme could be involved in catalytic regulation by divalent anions, similar to yeast PGK (see §3.1) [47,78].

### 5.2. Regulation by nucleotides

PGK activity is regulated by various nucleotides (AMP, ATP, ADP), as has been documented for different organisms [25,64,71]. Rabbit muscle PGK inhibition occurs by the presence of AMP, ADP, GDP, GMP, IDP and IMP. Apparently, the inhibitory action is of a mixed type [71]. This purine nucleotide-mediated regulation has also been observed in plants. The activity of cytosolic and chloroplast PGKs of *Pisum sativum* in the direction of ATP generation is regulated by AMP and ATP, while in the opposite direction (use of ATP), regulation occurs through AMP and ADP [136]. For yeast, there are references for the inhibition of PGK by ADP and AMP. Apparently, this enzyme has two nucleotide-binding sites, one binding site for the substrate ATP/ADP and another regulatory site for ADP separate from that for the nucleotide substrate. AMP also binds to the enzyme, probably at the same site as the substrate ATP. In addition, ADP is a competitive inhibitor, while AMP is a non-competitive inhibitor of 3PGA binding. This mechanism of inhibition might reflect an interesting way to regulate the direction of the enzyme's reversible reaction [137].

In bacteria such as *C. glutamicum*, inhibition of the ATP/3PGA-dependent reaction of PGK by ADP with a low Ki value (0.1 mM) has been documented [64]. The apparent mixed-type inhibition by ADP reveals the key role of PGK in gluconeogenesis. In trypanosomatids, regulation of PGK isoenzymes by nucleotides has also been reported [25]. *Trypanosoma cruzi* PGKC, a glycosomal enzyme, is inhibited at concentrations higher than 230 µM ATP, with a Ki of 270 µM. The inhibition of this isoenzyme by ATP has led to the proposal of a key role of this PGK in the regulation of glucose metabolism, specifically in gluconeogenesis. It is likely that this enzyme is optimized to work in the gluconeogenic direction even at low ATP concentrations by having a high affinity for this substrate. So far, this PGKC from *T. cruzi* is the only report that is available about a PGK of kinetoplastids regulated by ATP.

### 5.3. Regulation by redox state

In photosynthetic organisms, thioredoxin-dependent redox regulation of enzyme activities serves as a mechanism to control a large number of cellular processes, including the Calvin–Benson cycle. Most of the enzymes involved in this cycle are activated by light through dithiol/disulfide exchanges controlled by a chloroplastic thioredoxin. Several studies have shown that chloroplast PGK (chlPGK) is one of the enzymes undergoing such redox regulation [55,138]. Also in the cyanobacterium *Synechocystis* sp. (PCC6803), PGK activity is under redox control. A chloroplast-type thioredoxin (Trx) is responsible for maintaining this PGK in its reduced and active form, even under conditions of oxidative stress. Critical amino acids for this redox regulation are a cysteine pair, Cys314 and Cys340, located in the C-terminal domain of the *Synechocystis* PGK. The role of these two Cys residues for such regulation is linked to their proximity to the catalytic site and the associated conformational change that promotes their oxidized state [55]. In the unicellular green alga *Chlamydomonas reinhardtii*, a similar redox regulatory mechanism for chlPGK1 has been reported, mediated primarily by a Trx enzyme that is active during the light phase. However, structural analyses showed that the formation of disulfide bonds, mediated by two cysteine residues (Cys227 and Cys361), is a phenomenon that does not directly affect the affinity for the substrates; however, it has an obvious impact on the turnover and catalytic efficiency of the enzyme [139]. In addition to this redox regulation of the PGK, dependent on Trx, the chlPGK of *C. reinhardtii* was identified as a potential candidate for other post-translational redox modifications such as *S*-thiolation, glutathionylation and *S*-nitrosylation. It was found that some Cys residues, such as Cys361, may be subject to modification by glutathione. This means that these residues can also play a regulatory role under certain conditions or in the presence of specific other proteins [139–141]. It is possible that chlPGK can be regulated by multiple redox mechanisms. The complex interaction between the variable environmental conditions and the intracellular redox state perhaps determine the type and extent of each redox modification to which it is subjected. Additionally, these mechanisms could contribute to the fine adjustment of carbon fixation in photosynthetic organisms [139].

### 5.4. Regulation by non-coding RNAs

For tumour cells also, a regulation of PGK1 function by specific non-coding RNAs, microRNAs (miRNAs) and long non-coding RNAs (LncRNAs), has been reported [142–144]. In hepatocellular carcinoma (HCC), the dysregulation of miR-450b-3p induces inhibition of cell viability, colony formation and cell-cycle progression *in vitro*. This effect is attributted to the inhibition of PGK1 expression. Apparently, the 3′-UTR of the PGK1 gene contains a complementary sequence of miR-450b-3p; therefore, PGK1 probably is a direct target of miR-450b-3p. This regulation of the PGK not only has an effect on its expression, but also on its function through an inhibition of the PGK1-mediated AKT phosphorylation in HCC cells [142]. AKT is a kinase that acts as a key regulator of cell growth, cell-cycle progression and apoptosis [145]. In colorectal cancer cells, another miRNA, miR-548c-5p, suppresses proliferation by recognizing the 3′-UTR of *PGK1* to decrease the expression of this gene [143]. Unlike miRNAs, LncRNAs have an opposite effect on the function of PGK1 by acting at the posttranslational regulation level [144,146]. Through RNA pull-down assays and immunoblotting, it was confirmed that MetaLnc9 interacts directly with the PGK1 in lung cancer NSCLC cells. This interaction prevents PGK1 degradation by ubiquitination and stimulates the PGK1-activated oncogenic AKT/ mTOR signalling pathway, eventually promoting metastasis [144]. This mechanism of PGK regulation through direct interaction with an LncRNA has also been documented for gall bladder carcinoma (GBC). The direct interaction of LncRNA GBCDRlnc1 with PGK1 also prevents its ubiquitin-mediated degradation; however, in this case, the consequence of such interaction is the induction of chemoresistance of GBC cancer cells by activating autophagy. This activation could be mediated through the regulation of the autophagy-associated proteins ATG5 and ATG12 [146].

## 6. PGK as a moonlighting protein

**Figure 4.**
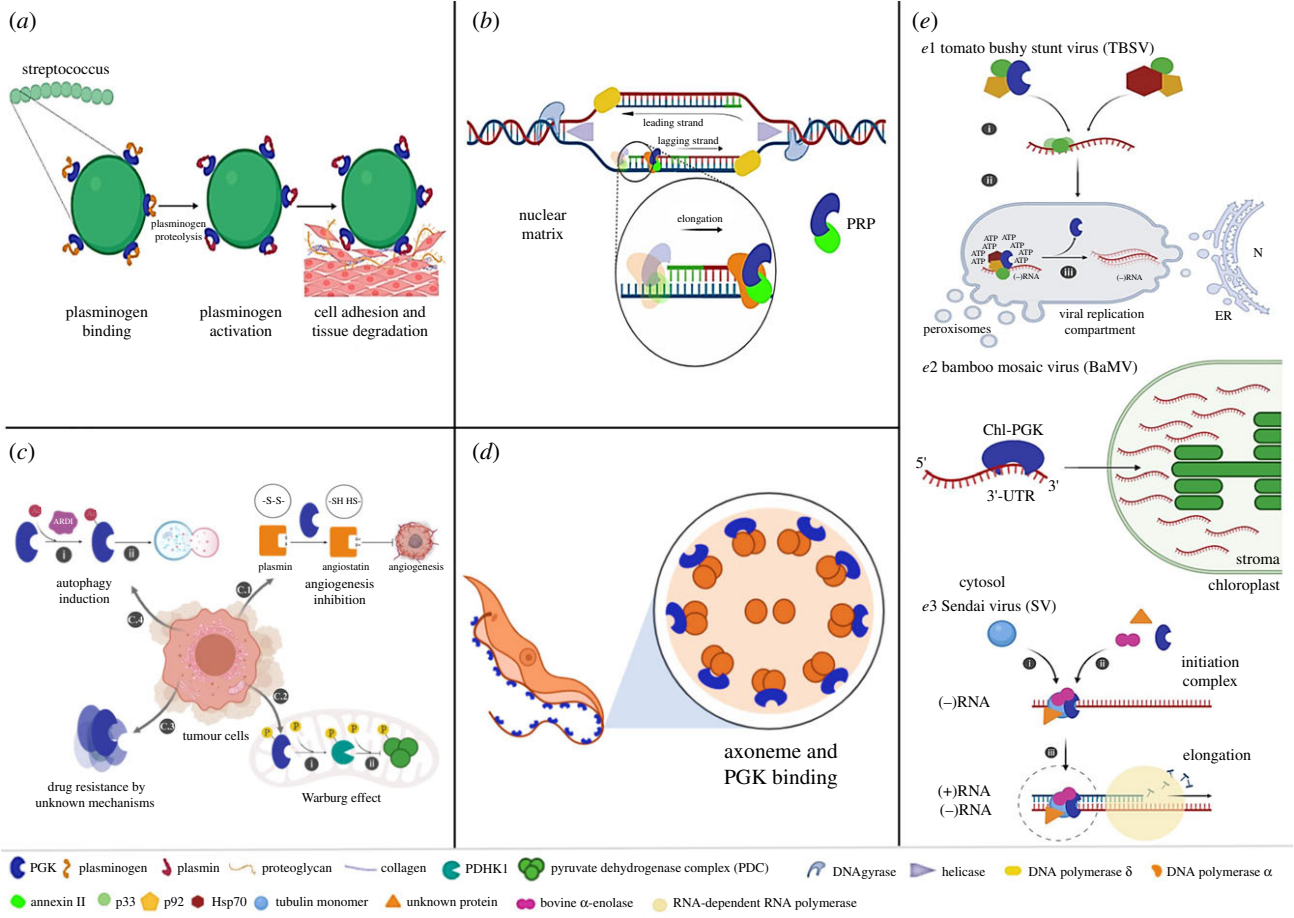
PGK moonlighting functions. (*a*) Cell invasion: PGK is located at the surface of some pathogenic bacteria where the processing of plasminogen to plasmin is promoted. (*b*) DNA replication: Together PGK and Annexin II protein constitute the Primer Recognition Particle (PRP) localized in the nuclear matrix. (*c*) Tumour growth and cancer progression: C.1. PGK is secreted from cancer cells. In the extracellular space, PGK allows the conversion of plasmin into angiostatin and the subsequent inhibition of angiogenesis. C.2. PGK phosphorylated at residue S203 is translocated into mitochondria to activate PDHK1 by phosphorylation (I). PDHK1 phosphorylates and inhibits the catalytic activity of the pyruvate dehydrogenase complex (PDC) and downstream metabolic reactions in mitochondria (II). C.3. Overexpression of PGK involved in drug resistance. C.4. PGK posttranslational modification (acetylation of residue K388) by ARD1 (I) induces PGK activation and subsequent phosphorylation of intermediate proteins that finally switch on the autophagy mechanism (II). Acetylation of other K residues contributes to increasing the glycolytic flux of tumour cells. (*d*) Functions associated with the flagellum. (*e*) Viral replication in TBSV: PGK and other host proteins are involved in viral mechanisms and able to bind to viral RNA. PGK also provides local ATP required in viral replication. (*e*.2) The bamboo mosaic virus (BaMV): chloroplast PGK (chlPGK) interacts with 3′-UTR of Viral RNA to direct the translocation of viral RNA from the cytosol to the chloroplast and allow its accumulation in the stroma. (*e.*3). In Sendai virus (SV): PGK participates in the initiation complex and stimulates viral gene transcription.

Moonlighting proteins comprise a heterogeneous collection of proteins from different classes that can perform multiple physiologically relevant biochemical or biophysical functions. Currently, more than 300 moonlighting proteins have been identified. These proteins are expressed in organisms throughout the ‘tree of life’, with their primary functions being attributed to different biochemical processes. Some of these proteins have the ability to perform their primary and secondary—moonlighting—functions simultaneously, while others change their function in response to environmental changes [147,148]. Moonlighting proteins can provide many potential benefits to an organism, such as the coordination of cellular activities. Proteins with moonlighting functions include receptors, transcription factors, adhesins and metabolic enzymes [149]. Among the metabolic enzymes, seven glycolytic enzymes have been identified as moonlighting proteins, one of them being PGK [150–152]. In various organisms, other functions in various cellular processes are attributed to PGK in addition to exerting its canonical function in glycolysis and gluconeogenesis, including roles in parasite–host relations (figure 4).

### 6.1. Cell invasion

In bacteria, PGK has been generally reported as a soluble, cytosolic protein, but a surface-associated form has also been described for the enzyme of several species. In some pathogenic bacteria, PGK has been identified as a major outer surface protein [35,153–156]. In isolates belonging to the genus *Streptococcus*, the enzyme is expressed on the cell surface, where it seems to be involved in pathogen–host interaction through binding to host proteins such as plasminogen and actin. In the case of plasminogen, the binding to streptococcal PGK occurs specifically through its angiostatin domain (kringle domains 1–4) [35]. It has been suggested that this interaction induces a conformational change in plasminogen that leads to its opening and facilitated conversion to plasmin [157,158]. Also, phytopathogenic organisms such as *Spiroplasma citri* contain a membrane-associated PGK that interacts with actin of the *Circulifer haematoceps* insect vector during the invasion of the bacterium into the insect's salivary glands [155]. Peptide mapping and site-directed mutagenesis studies indicated that the binding of the streptococcal PGK to both plasminogen and actin is mediated through three lysine-rich regions in the PGK structure [159]. This interaction is a key event for tissue degradation, cell adhesion and immune evasion during the infection process [35,59,156].

### 6.2. Nuclear roles

During evolution, cells have developed several strategies to detect and adapt to environmental changes, including those that cause changes in cellular energy levels. Studies have demonstrated the existence of a link between the metabolic flux and the regulation of gene expression. This relationship involves also moonlighting metabolic enzymes in the nucleus. Apparently, the existence of metabolic enzymes in the nucleus allows to provide information about the cell's nutritional and/or energy status by sensing the levels of metabolites and cofactors such as ADP, ATP and NAD(H) which can freely permeate from the cytosol to the nucleus through the nuclear pores. By their acquisition of also a role in the regulation of gene expression, such enzymes may link the supply and demand of energy and nutrients to gene transcription and so perhaps provide a way by which the cell can establish a quick and efficient adaptative response [160].

A role for the regulation of nuclear processes by the energy/redox state has been shown in various studies. For example, the redox state controls the activity of transcription factors such as NPAS2 and CtBP that are involved in the maintenance of mammalian circadian rhythms and in cell differentiation and development, respectively. This is exerted by differential binding of NAD^+^ and NADH to lactate dehydrogenase, acting as a cofactor with NPAS2, or directly to CtBP [161,162]. With regard to ATP, chromatin remodelling and several forms of hormonal gene regulation are known to require this cofactor [163].

There are several reports about the presence of glycolytic enzymes in the nucleus. To some of these enzymes, functions have been attributed associated with processes such as DNA replication and repair, and histone methylation and acetylation [160,164–171].

PGK has also been detected in the nucleus of both animal and plant cells [56,169,172,173]. In the pea *P. sativum*, both chloroplast and cytosolic PGK are also present in nuclei of leaf and root cells, where they were located primarily in the euchromatin. This location in the euchromatin might suggest participation with DNA polymerase α in replication. Both enzymes could act as nuclear receptors for cytosolic metabolites (e.g. ATP, ADP and/or phosphoglycerates) signalling the metabolic status of the cell [169]. Additionally, there are reports about mammalian cells where DNA synthesis catalysed by DNA polymerase α and ε on single-stranded DNA is stimulated by PGK. This activation might be due to a primer recognition capability of PGK. In this context, PGK, in complex with annexin II in the nuclear matrix, would act as a Primer Recognition Protein (PRP), with both proteins acting together as cofactors of DNA polymerase α, fulfilling a role in lagging strand DNA replication. [56,169,174]. An alternative suggestion is that PGK, in the presence of ATP, could regulate the replication complex through the phosphorylation of some proteins (such as DNA polymerase α-Primase B Subunit) [169] forming this complex that undergoes a cell cycle-dependent phosphorylation and dephosphorylation [175].

Together with other glycolytic enzymes in the nucleus, PGK could have a leading role in the modulation of repair and the replicative synthesis of DNA. By retaining their binding capacity for cofactors, substrates and inhibitors (which are mainly those of energy metabolism), one can imagine they establish a regulatory link between the energy status of a cell, and its DNA replication and repair functions [169].

### 6.3. Angiogenesis, tumour growth and drug resistance

There is growing evidence demonstrating that PGK performs, in some cases, a role in mediating tumour suppression mechanisms while in other cases promoting cancer initiation and progression, and drug resistance [53,57,176–178]. In different tumour cell lines, PGK participates in angiogenesis by acting as a disulfide reductase [57,176]. The PGK is secreted into the environment where it reduces plasmin that will generate angiostatin, a tumour angiogenesis inhibitor [57,179]. The formation of angiostatin can occur through mechanisms that involve the autoproteolysis of plasmin in alkaline conditions or due to proteases, specifically serine and metalloproteases [57,179]. However, when PGK is involved, the process seems to be a thiol-independent phenomenon which facilitates reduction in the disulfide bond(s) in plasmin [176]. This is a non-conventional mechanism due to the lack of the typical sequence Cys–Gly–X–Cys normally present in the active sites of protein reductants, but in this case involving predominantly hydrophobic interactions. Apparently, when PGK binds to plasmin, it induces a conformational change in kringle 5, by which the target disulfide bond (Cys-512–Cys-536) becomes accessible for nucleophilic attack. These events allow the exposure and proteolysis of target site residues in the plasmin's kringle 5 [176].

With regard to the initiation and progression of cancer, PGK can function as an oncogene, through its participation in oncogenic signalling pathways (such as AKT/Mtor, MYC, Nocth, CXCR4/β-catenin) [142,180–182]. Importantly, this enzyme and its post-translational modifications (PTMs), such as phosphorylation, acetylation, ubiquitination and succinylation, play a key role in regulating tumour metabolism. Depending on the PTMs to which this enzyme is subjected, there is an inhibition or induction of cell growth and progression [53]. PGK in tumour cells can behave like a canonical kinase, phosphorylating other proteins. Li *et al*. [183] discovered that in tumour cells, under certain conditions, translocation of PGK1 to mitochondria is induced. Once within mitochondria, this PGK1 functions as a PK, phosphorylating pyruvate dehydrogenase kinase (PDHK1). The subsequent phosphorylation of the pyruvate dehydrogenase complex (PDH) by this PDHK1 induces inhibition of the complex, subsequently causing suppression of the mitochondrial metabolism of pyruvate, thus promotion of the Warburg effect with an increase of lactate production [183].

On the other hand, drug resistance of tumour cells is associated with several factors, such as an enhancement of the cellular anti-apoptotic capacity, improved ability to repair DNA damage and an upregulation of the expression of the ATP-dependent drug transport protein. A significant amount of clinical and experimental evidence has shown that overexpression of intracellular PGK1 plays also an important role in resistance to a wide variety of medications and radiation [53]. The precise role of PGK1 in drug resistance mechanisms remains so far unknown. Some proposed suggestions are that it may be linked to increasing the glycolytic flux in response to drug-induced tumour starvation and intratumoral hypoxia, upregulating autophagy, triggering the HSP90/ERK pathway mediated DNA repair and methylation and/or scavenging ROS and inhibiting ROS-stimulated apoptosis [53].

In liver cancer cells, SIRT7 (sirtuin type 7) regulates the acetylation of PGK1 residue Lys323, a mechanism that appears to enhance the efficiency of the glycolytic pathway during the Warburg effect, and consequently promoting cancer proliferation and tumorigenesis [184].

### 6.4. Autophagy induction

A moonlighting role of PGK has also been found in its involvement in autophagy induction in human tumour cells, a process that requires its prior activation via lysine acetylation. Specifically, Lys388 is acetylated by the acetyltransferase ARD1 under glutamine deprivation and hypoxia conditions, when mTOR cannot inhibit ARD1 by phosphorylating its Ser228 residue. Subsequent to the ARD1-dependent PGK1 acetylation are the interaction with Beclin1 and the activation of the latter by its phosphorylation of residue Ser30, inducing activation of the Beclin1–VPS34 complex through conformational changes that increase the affinity of VPS34 to phosphatidylinositol. Hence, the generation of increased phosphatidylinositol 3-phosphate (PI3P) finally promotes the occurrence of autophagy [185].

### 6.5. Functions associated with the flagellum

The presence of a large (110 kDa) PGK-like protein (designated PGKL) and other proteins related to glycolytic enzymes associated with the flagellum of trypanosomatids suggests the possibility of the presence of a flagellar-associated glycolytic pathway that disappeared during early evolution of these parasites [60]. Studies involving a null mutant of *T. brucei* corroborate that PGKL's presence and function in the flagellum is not essential, at least in the procyclic, insect-stage form of the parasite and under the growth conditions evaluated. The PGKL was found to be tightly associated with the axoneme and most likely catalytically inactive since sequence comparison and structural analysis with functional PGKs demonstrated no conservation of key catalytical residues, similarly as observed for a homologous PGKL protein identified by genomic analysis of several other trypanosomatids and the distantly related kinetoplastid *Bodo saltans*. Brown *et al*. [60] suggested that glycolytic enzymes may have relocated from the flagellum to other cell compartments of the trypanosomatids (peroxisomes, giving rise to glycosomes), possibly as a response to environmental changes in the niche. Further details about the occurrence of this PGK-like proteins in kinetoplastid protists are discussed in §8 of this review.

### 6.6. Viral replication

PGK with moonlighting activity is also implicated in viral replication mechanisms [61]. Recruitment of host cellular components is promoted to allow the formation of replication particles of tomato bushy stunt virus (TBSV). TBSV replication requires the aggregation of peroxisome and ER compartments as well as the recruitment of several host proteins (Hsp70, Vps4p AAA+ ATPase, DEAD-box helicases) which participate in the assembly of the viral replicase complex, and in the activation of polymerases, as was evidenced for Hsp70. PGK is also recruited into this viral replication assembly. Its likely role was demonstrated to be the local supply of energy in the form of ATP for use by the molecular chaperones that promote efficient replicase assembly and activation of proteins involved in viral replication [61]. Interestingly, the supply of ATP during viral assembly is not the only function reported for PGK in viral mechanisms. In other studies, the enzyme was found to be involved in bamboo mosaic virus (BaMV) infection, interacting with the 3′-UTR of the viral RNA. Specifically, chloroplast PGK (chlPGK) was shown to be involved in the accumulation of BaMV in the *Nicotiana* cells, targeting the viral RNA to chloroplasts in the early stage of infection, to allow the viral replication within these organelles [186]. This chlPGK, together with the host heat shock protein Hsp90, translocate the viral ribonucleoprotein complex (RNP) across chloroplast membranes to the stroma. Subsequently, assembly of viral replication complexes (VRC) in association with the thylakoid membranes is stimulated [187].

Furthermore, it has been shown that bovine PGK, other mammalian PGKs and yeast PGK can successfully stimulate the *in vitro* synthesis and elongation of Sendai virus RNA. However, PGK catalytic activity appeared not required to stimulate this transcription, but in association with tubulin, the transcript elongation rate was drastically increased, as was also demonstrated for α-enolase [188,189]. Therefore, the experimental evidence summarized above demonstrates that PGK participates as a host protein factor implicated in different steps of the replication process of some viral entities.

## 7. Phosphoglycerate kinase in protists

PGK has been studied in detail in several protist organisms, both parasitic and free-living ones. In these organisms, the enzyme has been found in different subcellular compartments and different physiological functions have been attributed to it. Additionally, as we will see in the following sections, some of these organisms have different isoenzymes (table 3) and some of these PGKs undergo PTMs necessary to carry out their function.

### 7.1. Free-living protists

#### 7.1.1. Tetrahymena thermophila

*Tetrahymena thermophila* is a species of free-living ciliates, closely related to dinoflagellate and apicomplexan protists [190]. Like other ciliated protists, this organism is characterized by exhibiting nuclear dimorphism. It has a large non-germinal nucleus and a small nucleus which are cytologically and functionally different. Each of these nuclei is specifically used during the two different stages of the life cycle. The micronucleus (MIC) is transcriptionally silent and only plays a role during sexual life stages. It has a diploid genome consisting of five pairs of chromosomes, while the macronucleus (MAC) contains 200 different chromosomes that encode inheritable information passed from one sexual generation to the next [190–192]. The two most important functions attributed to the MIC are the generation of competitive sexual progeny and to provide genetic variation for improving the chances of survival of the progeny during periods of environmental changes [193]. For its part, the MAC is transcriptionally active, and its products maintain and control somatic cell functions during vegetative growth. The MAC chromosomes of *T. thermophila* encode multiple genes and exist, on average, in approximately 45 copies of each chromosome per MAC. The MAC genome directly determines the phenotypic characteristics of the cells [194]. Most genomic studies of *T. thermophila* have focused on the MAC genome [195–197]. When analysing the genome of this organism [85,108] we found two candidate genes that code for PGK enzymes (table 3). One of these isoenzymes is a canonical PGK [85,108,198], while the other is a multidomain PGK containing also a sequence related to the MYCBPAP protein family (i.e. proteins associated with binding the metazoan transcriptional activator Myc, so far not described for *T. thermophila*; table 3). Some of these MYCBPAPs, such as AMAP-1, associated with AMY-1 (amylase-alpha 1A) bound to Myc, may have a role in spermatogenesis in human testis cells [199].

Transcriptomic studies showed that both PGK genes are expressed in different phases of *Tetrahymena*'s cell cycle, during each of the three major physiological/developmental stages: growth, starvation and conjugation [108]. During the last stage, the gene TTHERM_00856750 (MYCBP-PGK) is differentially expressed in time, between 6 and 18 h after having initiated the sexual process. Its expression pattern is similar to that of other metabolic enzymes, and proteins such as kinesins, alkyrin, heterochromatin-binding proteins and TATA-binding proteins (TetraFGD, http://tfgd.ihb.ac.cn). In turn, the gene TTHERM_00929450 for the canonical PGK is only expressed 10 h after starting the conjugation. TTHERM_00929450 has an expression pattern very similar to other enzymes involved in glycolysis, the pentose-phosphate pathway and Krebs cycle. Notably, there are reports about the importance of these proteins in different cellular processes, especially chromosome segregation during mitosis and meiosis, programmed DNA elimination and MAC development [200–203]. One can imagine that this MYCBPAP-PGK is involved in any of the processes that occur during conjugation, when gene expression correlates with the stages of meiosis, nuclear differentiation and DNA elimination [204]. The expression levels of MYCBPAP-PGK increase near 6 h post-conjugation, coinciding with the time of new MAC development [201]. It is possible that this MYCBPAP domain allows us to activate the transcription of specific regions of the MIC genome related to the formation of the *T. thermophila* MAC. Therefore, this PGK could act as a moonlighting protein, fulfilling additional functions besides its metabolic one.

#### 7.1.2. Euglena gracilis

*Euglena gracilis* is a photosynthetic protist in which two isoforms of PGK with different subcellular locations were identified. One was located in the chloroplast (cpPGK) and the other in the cytosol (cPGK) (table 3) [110]. PGK was included in the 1345 candidate plastid proteins of a proteomic analysis [205]. The cpPGK isoform is synthesized as a precursor polyprotein, which yields two mature protein units (cpPGK) by its processing, after having been imported into the chloroplast. The processing is regulated by a thiol protease, located in the chloroplast stroma [206–208]. The result of this processing is two almost identical cpPGK proteins of 423 amino acids that differ in only one residue, Asp422 versus an Asn [110]. The strong similarity of *E. gracilis* cpPGK with its cytosolic homologues from other protists, animals and fungi suggests that this isoform is a molecular relic, in the sense that it is the only representative of the original cytosolic PGK found in chloroplasts among photosynthetic eukaryotes to date. With regard to the *E. gracilis* cytosolic PGK isoform, it is an orthologue of the cytosolic and glycosomal PGKs in the Kinetoplastea (see §8). Apparently, after an ancestor of the euglenid lineage within the Euglenozoa (figure 1) engulfed a chlorophytic alga, a non-endosymbiotic PGK replacement occurred in the *E. gracilis* plastid [110].

In addition to the cpPGK isoenzyme, other proteins such as the small subunit of RuBisCO (SSU) and the light-harvesting chlorophyll proteins (LHC) are synthesized as polyproteins in *E. gracilis* [209,210]. These proteins are part of a group of nuclear-encoded plastid-targeted proteins that are translated as polyprotein precursors. Once synthesized, they are routed to the chloroplast via complex N-terminal targeting sequences. Inside the chloroplast, the individual proteins are liberated through proteolytic cleavage of conserved decapeptide linkers [210,211]. Like for other chloroplast proteins, the expression, transport and processing of the SSU and LHC proteins are controlled by light and temperature [210,212]. In the case of cpPGK, some similar process is likely to happen. Studies based on Y_9_ mutants of *E. gracilis* have shown that the expression of this enzyme is influenced by light [213], although little is known about the molecular basis and function of this expression. Intriguingly, *Chlorella* chloroplast PGK exhibits circadian expression and appears to be an essential clock protein [214]. It would be interesting to study the physiological significance of a possible circadian regulation of PGK in *E. gracilis*. It could provide understanding of circadian regulation of glycolytic genes in trypanosomatids such as *T. brucei* [215].

#### 7.1.3. Dictyostelium discoideum

*Dictyostelium discoideum* is a free-living amoebozoan. It is a so-called social amoeba where, during its life cycle, individual protists can aggregate into a multicellular slug and then into a fruiting body, from which spores are formed that in turn differentiate back into amoebae. The single PGK present in this protist—called PGKA—is located in the cytosol as in most other eukaryotic organisms and has additional functions to its canonical ones. Sequence analysis revealed the presence of a putative calmodulin-binding domain in its structure, as reported by Myre & O'Day [128] and Catalano & O'Day [216]. The calmodulin-binding domain is situated next to the hinge and within the ATP-binding domain [128]. The activity of this PGK may be negatively regulated by calcium via calmodulin-mediated signalling (Ca^2+^-CAM-mediated signalling) [128]. Until now, the functionality and physiological significance of such putative regulatory mechanism in *D. discoideum* PGK are still unknown.

Calmodulin (CAM), being a sensor protein of intracellular calcium fluxes, controls metabolism in rabbit skeletal muscle through regulation of glycolysis and mitochondrial oxidative metabolism [217,218]. There are reports of glycolytic enzymes regulated by CAM binding [219]. Muscle phosphofructose kinase (PFK-M) is a CAM-binding protein. CAM atypically acts as a Ca^2+^-dependent inhibitor of PFK-M polymerization. The active tetrameric PFK is stabilized in an inactive dimeric form, upon inhibitor binding in the central region linking the two PFK dimers. This regulation by Ca^2+^-CAM-mediated signalling would lead to the existence of two PFK pools, a highly active pool of enzyme (A-conformers), and a pool of partially polymerized enzyme of very low catalytic activity in the D conformation [219,220]. In the case of PGKA of *D. discoideum*, it would be valid to think that the activity of this enzyme is probably modulated in response to Ca^2+^ signalling, similar to that has been reported for other glycolytic enzymes such as GAPDH associated with the sarcoplasmic reticulum membrane in skeletal muscle [221]. During the life cycle of this protist, Ca^2+^-CAM signalling plays important roles in processes such as chemotaxis, aggregation and development [222,223].

#### 7.1.4. Naegleria gruberi

*Naegleria gruberi* is a ubiquitous freshwater flagellated amoeba, characterized by drastic morphological changes, from having a true amoeboid form (which lacks a cytoplasmic microtubule cytoskeleton) to a flagellate (which has an elaborate microtubule cytoskeleton) [113]. Despite the few studies of some metabolic enzymes, so far little knowledge is available about the metabolism of *N. gruberi*. However, the sequencing of its genome and some transcriptomic studies allowed a broader view of the metabolic capacities of this protist [224,225]. Analysis of its genome suggested that this organism can use a wide variety of carbohydrates [225]. Experimental studies have shown that glucose is an important source of carbon for *Naegleria*; its presence stimulates growth and reduces the generation time [226]. Additionally, transcriptomic analysis revealed that genes involved in metabolism (including glycolysis, Krebs cycle and pyruvate–acetate metabolism) are differentially regulated during the differentiation process [224]. Although most regular glycolytic enzymes are present in *Naegleria*, this route apparently differs from that found in most other eukaryotic organisms [224]. The differences concern two aspects: (i) it lacks a hexokinase (HK), instead it has a glucose-specific glucokinase that is very similar to that found in trypanosomatids [225,227–229]; and (ii) the second phosphorylation step of glycolysis is catalysed by an inorganic pyrophosphate (PPi)-dependent PFK, instead of an ATP-dependent one [230]. Regarding the other glycolytic enzymes detected in the genome of this organism, a single *pgk* gene was identified [224]. It codes for a canonical PGK that is similar to those previously described for other protists [113]; table 3. It is noteworthy that *N. gruberi*, although it passes through significant morphological and physiological changes, contains in its genome only one gene for a PGK with a canonical structure. This enzyme is present in the cytosol [225], but *Naegleria* differentiation studies identified PGK, together with other glycolytic enzymes, also as a protein associated with the flagellum [113]. The expression of this enzyme occurs during the 60 min after the onset of the differentiation process, which coincides with the morphological transition from cysts to flagellates [113]. Although the function of PGK in the flagellum of *Naegleria* is unknown, the presence of glycolytic enzymes in flagella of other organisms (from protists to mammals) has led some authors to suggest that these enzymes may provide a localized source of ATP that is essential for cellular motility [60,231].

*Naegleria* catabolizes glucose through glycolysis and the Krebs cycle, with the electrons of NADH and FADH_2_ fuelling the branched respiratory chain, while fatty acid catabolism occurs through β-oxidation followed by respiratory chain activity [232]. Though *N. gruberi* contains all genes necessary for the metabolism of amino acids, fatty acids and carbohydrates [225], this protist, during its growth, exhibits a notable preference for fatty acid oxidation as the main source for ATP synthesis. Like in other organisms, fatty acid oxidation occurs in both peroxisomes and mitochondria [232]. This preference of nutritional source is a distinctive feature of *Naegleria*, not documented for other pathogenic protists [232], although the role that glycolysis plays in covering the metabolic needs of the related pathogenic species *N. fowleri* is poorly understood. It has been shown that glucose can be an important source of carbon for this protist during human infection [233,234]. Trophozoites cultured in medium without glucose leads to a reduction in growth and induces encysting. Additionally, the addition of 3-bromopyruvate, a well-known inhibitor of glycolysis in tumours, to standard glucose-containing medium, also induces a dramatic decrease in the growth of this protist [234], in a similar way as in trypanosomes [235].

The PGK enzymes from the free-living protists described in §§7.1.1–7.1.4 have very similar characteristics to each other, except for the presence of an additional domain in the case of one of the PGKs of *T. thermophila* (MYCBPAP) and *D. discoideum* (calmodulin-binding domain). They have a similar number of amino acids and molecular weight (between 45 and 46 kDa, except for the *E. gracilis* cpPGK, which has a weight of 105 kDa before being processed), and apparently, most have only the proper PGK domain associated with its structure (table 3). In some cases, it has a dual subcellular localization, in the cytosol and essential organelles such as the *E. gracilis* chloroplast and *N. gruberi* flagellum (table 3). In these free-living organisms, the PGK seems often to be linked to functions beyond glycolytic/gluconeogenic metabolism, such as catalysis of the reverse PGK reaction as part of the Calvin cycle and with a regulatory role (for PGK functioning as a glycolytic/gluconeogenic enzyme) controlled by Ca^2+^-CAM signalling. Through its pattern of expression, it may also be involved in processes like the circadian cycle in some of these free-living protists.

### 7.2. Parasitic protists

#### 7.2.1. Amitochondriate protists

*Entamoeba histolytica*, the aetiological agent of amebiasis, depends exclusively on glycolysis for the generation of ATP, because it lacks the Krebs cycle and oxidative phosphorylation [236–239], a metabolic adaptation shared with two other pathogenic amitochondriate protists, *Giardia lamblia* and *Trichomonas vaginalis* [238]. Strikingly, these parasites, living in anaerobic/microaerophilic environments, use PPi instead of ATP as the high-energy phospho group donor in glycolysis [230]. *Entamoeba histolytica* has an unusual PGK. This monomeric PGK with a canonical structure (table 3) is selective for guanine nucleotides rather than adenine nucleotides [21]. The extent of the preference for GTP varies with the direction of the reaction that this enzyme catalyses. In the glycolytic direction, the selectivity for GTP over ADP is 150-fold, whereas in the gluconeogenic one, it is about 50-fold [240]. The presence of a guanine nucleotide-dependent PGK, responsible for the first substrate-level phosphorylation reaction of glycolysis, could most likely be indicative of changes in its interplay with other metabolic pathways and cellular processes in the parasite [21]. In addition, this peculiar nucleotide preference may have physiological relevance. The simultaneous presence of a GTP-dependent PGK and a nucleoside diphosphate kinase, previously identified [241], should be able to make ATP readily available from the GTP pool [21]. Alternatively, since *E. histolytica* lacks a de novo purine synthesis pathway [242], this PGK could serve to provide a source of GTP for DNA synthesis.

The PGKs of *G. lamblia* and *T. vaginalis* have been less well studied. *Giardia lamblia* has a PGK which is more alike to those found in most other eukaryotes [243]. For *T. vaginalis,* there have been several molecular and biochemical studies related to PGK. Two candidate *pgk* genes have been reported [114,244]. The encoded enzymes are 98% identical, have the same predicted molecular weight, a similar pI and both are apparently catalytically active (table 3). Probably, they are the products of a recent *pgk* gene duplication. In previous studies, it has been documented that a distinctive feature of the *T. vaginalis* genome is the retention of multiple paralogous copies of some genes, including those encoding glycolytic enzymes [244]. The expression of these isoenzymes is influenced by environmental factors such as temperature and nutritional condition [114,244,245]. PGK is one of the enzymes with a relatively high activity in *T. vaginalis* and, unlike other glycolytic enzymes (HK, aldolase (ALD), enolase (ENO), phosphoglycerate mutase (PGAM)), its activity is not affected by the presence or absence of oxygen [246]. Additionally, transcriptomic studies focused on regulation mechanisms of metabolic pathways have shown that the expression of the two PGK isoenzymes is downregulated under limited availability of glucose [114]. Additionally, the glycolytic pathway is apparently one of the major iron-regulated pathways in *T. vaginalis*. Transcription of at least one (but usually several) of the multiple gene copies encoding enzymes involved in glucose metabolism showed significant iron-dependent regulation. Especially those enzymes that supply substrates to the pathways involved (such as glucokinase, glycogen phosphorylase and phosphoglucomutase), as well as some other enzymes such as PGK, are upregulated [244]. This could be related to the fact that establishment of *T. vaginalis* infections is dependent on the efficient acquisition of essential nutrients such as iron from the host environment. The iron ion performs important functions in the cytoadherence of *Trichomonas* to host target cells [247], the expression of diverse proteinases [248], and it increases resistance to complement-mediated lysis [249].

#### 7.2.2. Apicomplexan protists

In the genome of the apicomplexan organism *Toxoplasma gondii*, genes for all glycolytic enzymes from PFK down to PYK are present as duplicates. They code for proteins with different subcellular localization, with one set of enzymes constituting a complete pathway in the cytosol [115]. The expression of the two PGK isoforms (table 3), PGKI and PGKII, shows no significant difference between the two life cycle stages living in the warm-blooded vertebrate host, the rapidly proliferating tachyzoites and the slowly proliferating, encysted bradyzoites. Interestingly, PGK II (and isoenzymes of some other glycolytic enzymes) has a peculiar localization, within the apicoplast, a non-photosynthetic plastid relict characteristic of this group of organisms (except in *Cryptosporidium—*see below—that has lost it) [115]. PGK II serves to convert 1,3BPGA to 3PGA that is subsequently transferred to the cytosol through a system (pPTS) that transports phosphorylated glycolytic intermediates in antiport with inorganic phosphate (Pi) across the inner envelope membrane [250]. In the cytosol, this 3PGA can be converted to PEP that could be directed to the apicoplast through a PEP/Pi translocator (PPT) where it is converted to pyruvate by PYK. The pyruvate is then used as a substrate for the pyrvuvate dehydrogenase (PDH) to produce acetyl-CoA, necessary for the synthesis of fatty acids [250].

In *P. falciparum*, the agent that causes malaria, anaerobic glycolysis is known already for long time as the main source of ATP in the erythrocytic stage, while oxidative phosphorylation may play a more important role in the mammalian liver and mosquito vector stages [251,252]. Thus, the parasite's enzymes of the glycolytic pathway are essential for the development of its asexual blood stages [20]. The PGK has been characterized at the molecular, structural and biochemical levels [20,253,254]. Our genome analysis demonstrated the presence of a single *pgk* gene on chromosome 9 of this parasite, in agreement with the result previously reported by [253]. The crystal structure of this glycolytic enzyme in the open conformation is similar to the structure of the monomeric PGKs from other organisms [47] (table 3). However, the kinetic properties of the parasite enzyme differ from those reported for its human counterpart. This has led to consider it as a potential target for chemotherapy. It has a greater affinity for its substrates than the human PGK. Additionally, it can use different divalent cations (Ca^2+^/Mg^2+^/Mn^2+^) for its activity, showing a preference for Mg^2+^ [254]. One of the most distinctive features of this *P. falciparum* PGK is its high temperature sensitivity that has been attributed to a change in the proportion of basic amino acids in its structure [253]. Unlike the PGK of some trypanosomatids such as *T. brucei*, the *P. falciparum* PGK is activated in the presence of sulfate anions (as Na or K salts) [254].

*Cryptosporidium parvum*, an obligate intracellular parasite, can be a major cause of diarrhoea in human and mammals [255]. This parasite has a 9.4 Mb genome with 3807 protein coding genes [256]. Species of the genus *Cryptosporidium* have lost the apicoplast (and metabolic pathways associated with this organelle) as well as many of their mitochondrial metabolic capabilities, which causes that they depend even more on host cells to get some basic nutrients and rely heavily on glycolysis for their ATP generation [256–258]. However, this is not a general rule for all *Cryptosporidium* species. *Cryptosporidium marinus* and *C. andersoni* contain all enzymes of the Krebs cycle and a conventional respiratory chain [14,258].

*Cryptosporidium* possess genes for all 10 enzymes of glycolysis in the genome [259] and all enzymatic activities of this pathway, except for hexokinase, were detected in the cytosolic cell fraction [257]. PGK of this organism has been little studied. However, the databases (e.g. http://CryptoDB.org) list, in addition to a canonical PGK (CPATCC_0006650) with molecular weight 42 kDa and all key residues for activity, at the same chromosome 7, a very different 45 kDa PGK-family protein (CPATCC_0006130), which contains the Walker A and B motifs for ATP binding (table 3), but seems to lack any features specific for PGK activity. The metabolic function of this latter protein remains unknown. However, transcriptomic studies suggested that both enzymes may have a role in survival and resistance to environmental stress [260].

Studies of other apicomplexans of economic and clinical interest such as *Babesia*, the causative agent of babesiosis in many vertebrates including human and transmitted by ticks [261], have revealed important data about this protist. *Babesia bovis* has a genome with a size of 9.4 Mb that is organized in four chromosomes [262,263]. It is one of the smallest apicomplexan genomes found so far. Several genes associated with metabolic pathways (such as glycolysis, the Krebs cycle and oxidative phosphorylation) have been identified [264,265]. However, unlike in other protists, many pathways, such as fatty acid oxidation and de novo purine, haem and amino acid biosyntheses, are apparently absent [264]. In addition, proteomic analysis of erythrocytic stage cells of *B. microti* has revealed a significant reduction in its metabolic potential. During this life cycle stage, *B. bovis* and other species depend on glucose fermentation for their energy production and redox balance [117,265–267]. Some enzymes involved in the metabolism of *Babesia* have been studied [268]; however, so far there are only a few reports on its PGK. It is one of the glycolytic enzymes identified in *B. microti* through transcriptomic studies [265]. The enzyme is encoded by a gene located on chromosome 3 and has characteristics of a canonical PGK, with no other domains associated with its structure (table 3). Therefore, its function is most likely limited to generating ATP in the glycolytic pathway. Studies of *B. gibsoni* showed that diminazene aceturate (DA)-resistant isolates have an increased glucose consumption and intracellular ATP level compared to wild-type isolates [269]. Although the mechanism by which the glycolytic activity and ATP concentration are increased is still unknown, it would be interesting to analyse candidate enzymes that confer this characteristic to DA-resistant *B. gibsoni* isolates.

#### 7.2.3. Stramenopile protists

*Blastocystis* belongs to the non-photosynthetic stramenopile group of protists containing also free-living unicellular algae and diatoms. But *Blastocystis* is an anaerobic parasite responsible for gastrointestinal infections in humans and a large variety of animals worldwide. Some studies have been conducted on the subcellular localization of its glycolytic enzymes, where both cytosolic and mitochondrial forms of some of these enzymes have been found [270]. When analysing the genome of this parasite, we found a single gene that codes for a PGK with a canonical structure, with a possible localization in its mitochondrial remnants (mitochondrial-related organelles, MROs) (table 3). These results concur with previous reports by Nakayama *et al*. [271] and Abrahamian *et al*. [272]. A bioinformatics analysis of 72 genomes covering 13 eukaryotic groups about the subcellular distribution of all glycolytic enzymes predicted that not only cytosolic but also mitochondrial forms of glycolytic enzymes are present in several representatives of the stramenopiles [271,272]. However, only the ‘payoff-enzymes’, i.e. enzymes of the second part of the pathway responsible for producing ATP and intermediates for lipid and amino acid biosynthesis, were located in both compartments of the stramenopiles [272]. In *Blastocystis* sp., this second half of the glycolytic pathway is even solely localized in its MROs [273].

The PGK of *Blastocystis* has a cyanobacterial origin [274] and is present in its MROs, along with the other enzymes (GADPH, PGAM, ENO, PYK) of the ‘payoff phase’ of glycolysis. In most stramenopiles, the compartmentalization of these glycolytic enzymes within mitochondria goes along with that of enzymes involved in the synthesis of serine (phosphoglycerate dehydrogenase, phosphoserine aminotransferase and phosphoserine phosphatase). This co-compartmentalization may facilitate the transfer of intermediates between both pathways, since they share a common intermediate, 3PGA. Whereas in most eukaryotes, the enzymes of serine biosynthesis, like those of glycolysis, are present in the cytosol, such cytosolic localization of the amino acid biosynthetic enzymes is missing from oomycetes and most other stramenopiles [272]. However, *Blastocystis* does not possess a serine biosynthesis pathway; its ancestors may have lost it with when adapting to an anaerobic parasitic lifestyle and reducing their mitochondria to MROs, while retaining the glycolytic ‘payoff’ enzymes in the organelles.

Most of the PGKs identified in the parasitic protists described above in this section have a canonical PGK structure. These enzymes have molecular weights ranging between 45 and 63 kDa. Most of these enzymes are present in the cytosol, with some exceptions having a localization in the mitochondria or MROs and the apicoplast. In the case of these parasitic protists, these glycolytic enzymes seem to be much more often linked to functions beyond metabolic ones, such as de novo purine synthesis, virulence, protein homeostasis, functions that have not been observed for these enzymes in free-living protists.

## 8. Diplonemid and Kinetoplastid protists

### 8.1. Diplonemids

Diplonemids form a group of biflagellate, heterotrophic protists, most of them inhabiting marine ecosystems. These organisms, together with the euglenids (§7.1.2) and kinetoplastids, constitute the Euglenozoa phylum (figure 1). Diplonemids, which also include some pathogenic taxa [275], are considered metabolic versatile organisms. Genomic studies have shown that these organisms are characterized by having the most protein-encoding-rich transcriptomes when compared with euglenids and kinetoplastids. Of its 43 107 predicted proteins, approximately 2.55% correspond to metabolic enzymes, of which 246 are unique to diplonemids [276]. In *D. papillatum*, the presence and localization of glycolytic enzymes have been documented [118,277]. Several glycolytic enzymes of this organism, such as glucokinase, glucose-6-phosphate isomerase, TIM and PGK, have been associated with peroxisome-related microbodies called glycosomes, and their sequences contain a canonical type 1 peroxisomal-targeting signal (PTS1) at their C-terminus. Glycosomes (i.e. peroxisomes containing glycolytic enzymes) have previously been found in all Kinetoplastea studied (see next sections). The finding of these enzymes also in glycosomes of diplonemids, while not in *E. gracilis*, provides evidence for the notion that such compartmentalization originated in the common ancestor of diplonemids and kinetoplastids [118]. Whereas *D. papillatum* seemed to lack a fully functional glycolytic pathway (HK and PFK were not expressed), this protist is able to perform gluconeogenesis [118]. The gluconeogenic flux would be sustained by the use of amino acids as sources of energy and carbon. These are catabolized via the Krebs cycle, and the metabolites subsequently used for anabolic purposes in the gluconeogenic and pentose–phosphate pathways [118]. Retention of a full spectrum of genes encoding biosynthetic pathways allows *Diplonema* to obtain its amino acids both from nutritional sources and through biosynthesis, a property that could be an important factor for the ecological success of the diplonemids, since amino acids are the main nutritional component in the phagocytic diet of these heterotrophic organisms [118,276].

When analysing genes for glycolytic enzymes of *D. papillatum*, we obtained results similar to those previously reported. Some of these genes, such as for PFK and GAPDH, are duplicated [118]. Three ORFs were found for PGK, one of them (BAQ25443) coding for a fully functional PGK with a canonical PTS1 (a C-terminal tripeptide -SKL) (table 3). This PGK was located in glycosomes where it can perform the catalytic reaction in the gluconeogenic direction [118]. In addition, at least two ORFs that encode PGK-like proteins have been identified. These ORFs are located in different parts of the genome and encode proteins with molecular weights of 42 and 26 kDa. Both products appear to be catalytically ‘dead’ PGKs and lack a canonical PTS sequence (data not shown). The absence of activity of these PGKs may be due to redundancy associated with the adaptation to the heterotrophic lifestyle and development of the gluconeogenic state of *D. papillatum*.

### 8.2. Kinetoplastids

PGK is one of the most studied enzymes of kinetoplastids. Not only because it is one of the enzymes responsible for generating ATP in the glycolytic pathway that for its major part is compartmentalized within glycosomes, but also because multiple genes and PGK isoenzymes have been detected in these organisms [24,25,122,278]. Additionally, in many of the Kinetoplastea species studied, most of the genes for these isoenzymes are organized in tandem [23,80,279]. However, these duplicated, tandemly arranged genes show variations in their sequences. Such tandem arrays have the capacity to harbour important variation for enzyme function and subcellular localization [280]. Variations may occur in the subcellular localization of the PGKs in different species and in their expression in the successive developmental stages of these kinetoplastids [60,119,125,279,281]. The characteristics of these PGK genes and their products are summarized in table 3.

#### 8.2.1. Free-living kinetoplastids

##### 8.2.1.1. Bodo saltans

*Bodo saltans* is a free-living kinetoplastid that is evolutionarily related to trypanosomatid parasites. A comparative study of the genomes of *B. saltans* and parasitic trypanosomatids revealed that the transition from a free-living to a parasitic lifestyle has resulted in the loss of approximately 50% of protein-coding genes [282]. This protist has genes for several PGKs, like the parasitic kinetoplastids [24,25,60,122,278]. However, its three *pgk g*enes are present in three separate loci [282]. Of these three genes, one (SAL_14730) codes for a potentially active enzyme of 56 kDa that is predicted to be present in glycosomes. The other two ORFs code for isoenzymes that both exhibit an unusual modular architecture, being multidomain PGKs in which, respectively, two and three different domains are combined (table 3). The gene SAL_29930 codes for a 57 kDa PGK with, at its N-terminal side, an extra PAS domain, similar to the enzyme that previously has been reported for *T. cruzi* and *Leishmania major* (see also sections below). In *T. cruzi*, a function for sensing environmental conditions has been attributed to this PAS-PGK protein, while in *Leishmania*, it is associated with adaptation to pH change, virulence and cell survival through autophagy [4,28]. The PAS-PGK seems to have different subcellular localizations in *B. saltans* and the two parasitic kinetoplastids; in *B. saltans*, bioinformatics analyses predict PAS-PGK to be a cytosolic protein, while in *T. cruzi*, it is associated with glycosomes, and in *Leismania*, it appears to have a dual localization, in glycosomes and the lysosome [4,28,104]. In turn, gene BSAL_70390 encodes an 89 kDa PGK-like protein with a cAMP-binding domain (CNB) and a transmembrane domain. It seems orthologous with the PGKL enzyme reported for *T. brucei* [60], as discussed in §6.5 and below in §8.2.3.2.1, although it lacks the seemingly relict DNA-binding domain present in the protein of the latter and some other trypanosomatid species. In both protists, the protein has as distinctive feature that the PGK domain lacks key catalytic residues; in *B. saltans*, the PGK domain has specifically lost residues necessary for 3PGA binding. In addition, in *B. saltans*, this PGK is predicted to be targeted to a membrane (table 3), while in *T. brucei*, this enzyme has been located in the flagellum [60], along with some other glycolytic enzymes, such as a GAPDH isoform. The finding of these similar multidomain PGKs in bodonids and trypanosomatids indicates that their presence pre-dates the divergence of the parasitic trypanosomatid family. Although the presence of orthologues of PGK with a CNB and one or more membrane-spanning domains is not limited to *T. brucei* and *B. saltans*, it seems to be absent in several other kinetoplastids such as *Perkinsela* sp. and *Leishmania* spp. Moreover, its subcellular location seems to have undergone changes during the divergence of these parasites (table 3; electronic supplementary material, table SI). Although a possible explanation previously proposed for the presence of this flagellar degenerated PGK enzyme in *T. brucei* is a change to glycolysis regulation and compartmentalization during kinetoplastid evolution [60], we suggest that it may rather be related with a protein that acts as a ‘functionally flexible’ enzyme, fulfilling different cellular tasks not related to the proper PGK function, but adapted to the environmental and nutritional requirements of the different groups of kinetoplastids. This would be the reason why it is conserved in most kinetoplastids, although it is catalytically not functional. This type of enzyme can fulfil a wide variety of functions, without depending on his catalytic properties [283–285].

#### 8.2.2 Endosymbiotic kinetoplastids

##### 8.2.2.1. *Perkinsela* sp.

*Perkinsela* sp. is a kinetoplastid living as an endosymbiont within the amoebozoan *Paramoeba pemaquidensis* that infects marine animals. Its nuclear genome is approximately 9.5 Mb [286], much smaller than that of *B. saltans* [5], *Phytomonas* sp. [287], *Leptomonas pyrrhocoris* [288] and human parasites such as *T. brucei* [289], *Leishmania* spp. [290] and *T. cruzi* [291]. *Perkinsela* sp. is a sister group of *Ichthyobodo*, both of which make up the group of the Prokinetoplastea within the Euglenozoa phylum (figures 1 and 2). They represent an early branching lineage within the Kinetoplastea. By the robust branching of the clade base, the stable intracellularity of *Perkinsela* can be considered as an ancestral form of parasitism [292]. Although genomic analyses showed that *Perkinsela* sp. has lost the ability to develop a flagellum, as do other kinetoplastids, it retains hallmark features of kinetoplastid biology, included glycosome-like organelles. While the repertoire of its genes and proteome is markedly reduced, *Perkinsela* sp. possesses kinetoplastid-specific biochemical pathways [286]. With regard to the presence of glycosome-like organelles, various genes coding for PEX proteins involved in glycosome biogenesis, including both membrane proteins and cytosolic ones (such as the receptors for import of matrix proteins with a type 1 PTS (at the C-terminus) or type 2 (close to the N-terminus) PTS), were identified in the genome of this endosymbiont. Additionally, putative PTS1 or -2 motifs were identified in the first seven enzymes of the glycolytic pathway encoded in its genome [286]. Like in other kinetoplastids, in addition to glycolysis, other biochemical processes are predicted to occur in the putative glycosomes of *Perkinsela* sp., which include amino acid, nucleotide and sterol/isoprenoid metabolism. PGK is one of the glycolytic enzymes identified in *Perkinsela* sp. Apparently, this organism has only one gene coding for a PGK [286], which could have two explanations. First, PGK isoenzymes present in the other kinetoplastids were lost in *Perkinsela* sp. as a result of its adaptation to an endosymbiotic lifestyle. Alternatively, *Perkinsela* represents the ancestral situation and the multiple isoenzymes were acquired by free-living kinetoplastids and parasites with complex life cycles. The *Perkinsela* enzyme has a canonical structure of 58 kDa, with only a PGK domain associated with its structure; it has all residues required for the catalysis and contains a PTS1 (-GKL).

*Perkinsela* sp. also lacks the PGK isoforms with possible capacity of sensing environmental conditions domains, such as the PAS-PGK (table 3). Intriguingly, this PGK isoenzyme with the domain related to the sensing of certain environmental conditions is also absent in *T. brucei*. Although this parasite alternates during his life cycle between two hosts, it is permanently extracellular, while other kinetoplastids, such as *Leishmania* spp. and *T. cruzi*, not only have an alternation of host but also, during certain stages live intracellularly.

#### 8.2.3. Parasitic kinetoplastids

##### 8.2.3.1. Paratrypanosoma confusum

*Paratrypanosoma confusum* is a monoxenous kinetoplastid isolated from the gut of *Culex pipiens* mosquitoes [293]. Evolutionarily, it represents the most basal trypanosomatid lineage branching between the free-living *B. saltans* and the parasitic trypanosomatids [293,294], and may thus provide insight into the emergence of parasitism. Preliminary studies of its genome showed that it shares at least 114 genes with 15 species of trypanosomatids, *B. saltans* and the heterolobosean *N. gruberi* and 129 protein genes with the endosymbiotic *Perkinsela* sp. [293]. *Paratrypanosoma* and stercorarian trypanosomes, including *T. cruzi* and *Trypanosoma grayi*, retain more ancestral genes than other trypanosomatid clades [294].

When analysing the *P. confusum* genome, we found two genes (PCON_0085140 and PCON_0003620) encoding PGK-like proteins, with sizes of 28 and 104 kDa, respectively (table 3). Both PGKs are apparently catalytically inactive, due to substitutions and deletions of residues essential for substrate binding. The predicted 104 kDa protein has the same domain organization as the 89 kDa PGKL of *B. saltans*, although the overall amino acid sequence identity is only about 39%. Surprisingly, no gene for a conventional PGK was detected. Until now, there have been no metabolic studies of *P. confusum*. However, it is very likely that its network for intermediary metabolism is organized very similarly as that in other kinetoplastids, with sequestering of a major part of the glycolytic pathway within glycosomes, because homologous genes encoding enzymes like HK, ALD and GAPDH, each with a PTS, are present in the genome. The absence of a functional PGK is most likely to be due to incompleteness of the genome data. Less probable alternatives are that this parasite contains a very different PGK not recognized in our bioinformatics analysis or has some mechanism to bypass the catalytic passage of this enzyme. An active PGK or bypass for it should be necessary, because genes for seemingly functional downstream glycolytic enzymes (PGAM, ENO, PYK) are present in the *P. confusum* genome. A possible bypass that could be considered is the Rapoport–Luebering pathway, which is best known from its functioning in erythrocytes, and has also been documented in protists like *D. discoideum* [295]. This pathway comprises evolutionarily conserved reactions that generate and dephosphorylate 2,3-bisphosphoglycerate (2,3BPGA), which in turn is transformed to 3PGA. These two reactions are catalysed by the 2,3BPGA synthase/2-phosphatase (BPGM) enzymes and the multiple inositol polyphosphate phosphatase (MIIP1), respectively [295]. For *Dictyostelium*, it has been demonstrated that these enzymes provide a physiologically relevant regulation of cellular 2,3BPGA content. They also establish a molecular link between the turnover of phosphorylated inositol derivatives and glycolytic flux [296]. However, genomic searches did not reveal indications for its existence in this organism or any other kinetoplastid.

##### 8.2.3.2. PGK in African trypanosomes

**Figure 5.**
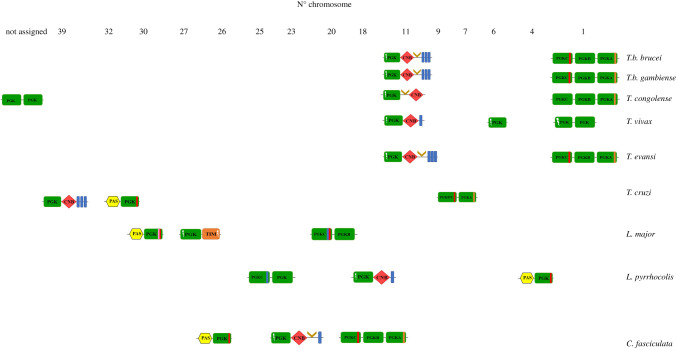
Genomic location of *pgk* genes in different species of Kinetoplastea. Determination of the chromosomal localization of identified *pgk* genes was performed using the Kinetoplastid Genomic Resource (TriTrypDB) [94]. Identification of domains and motifs present in PGK sequences was done using the following bioinformatics servers: *PAS domain*: Protein Blast (NCBI) [96], InterProScan (EMBL-EBI) [95,97] and Simple Modular Architecture Research Tool (SMART) [98]; *CNB domain*: NCBI, EMBL-EBI, SMART; *HTH (Helix–Turn–Helix)* motif: ExPaSy [99], GYM [100], iDNA-Prot [101]; *Membrane helix*: SMART [98]; Predicting transmembrane protein topology with a hidden Markov model (TMHMM prediction) [102] and Phobius [103]. Glycosomal localization prediction was based on recognition of a PTS1 consensus sequence as reported by Acosta *et al*. [104]. *Domain symbology:* PGK domain 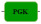, PGK domain having lost substrate (3PGA)-binding residues 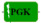, triosephosphate isomerase domain 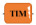, HTH domain 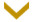, transmembrane segment 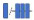, PAS domain 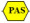, cyclic-nucleotide binding-domain 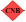. Import sequences and inserts identified in PGK isoenzymes through experimental and theoretical studies: putative PTS1 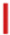, putative lysosome 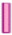, PGKA-like insert sequence 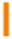.

8.2.3.2.1. *Trypanosoma brucei. T. brucei* has three *PGK* genes (A, B and C) organized in tandem on chromosome 1 (table 3 and figure 5). The *pgkA* gene encodes a variant (PGKA) that is constitutively present at a low level within the glycosomes of both the mammalian bloodstream and insect procyclic forms of this parasite [120,297]. The second gene, *pgkB*, encodes the cytosolic enzyme PGKB, found at considerable concentration but only in procyclic forms, and the third gene, *pgkC*, encodes the major glycosomal enzyme PGKC, abundantly present only in bloodstream forms [23,119]. PGKC is directed to the glycosomes by a PTS1-like sequence present at the end of a 20-amino-acid C-terminal extension. The gene cluster *pgkA–pgkB–pgkC,* located on chromosome 1, is part of a long multicistronic transcription unit. However, the different concentrations of the individual mRNAs during the life cycle are controlled post-transcriptionally [23,80].

The PGKB and PGKC gene products are monomeric proteins with molecular weights of 45 and 47 kDa, respectively. Their amino acid sequences are 95% identical, and they share approximately 45% identity with other, non-kinetoplastid eukaryotic PGKs [119]. Unlike PGKs from yeast and rabbit muscle, which can also use GTP and ITP, the *T. brucei* enzymes are strictly ATP dependent [22]. This specificity for ATP as substrate is related to substitution of amino acids in the structure of these isoenzymes, what probably prevents phosphoryl-group transfer during catalysis in the case of the other nucleotides*.* PGKA is monomeric protein with around 60% identity to the PGKB and C isoforms, and has the distinctive feature of an 80 amino acids long insertion in its N-terminal half. Part of the amino acid sequence (position 24–91) of this insertion has been shown necessary for glycosomal targeting of this isoenzyme, although so far, no specific sequence motif for it has been identified. It has been suggested that the glycosomal entry of PGKA is by virtue of its interaction with a protein that does contain an import signal [297]. This enzyme is not only less abundant than the PGKB and PGKC isoenzymes but has also negligent catalytic activity [121]. All these PGKA/B/C isoenzymes of *T. brucei* have very similar substrate affinities (table 2). The Km for ATP varies between 0.12 and 0.4 mM, while for 3PGA, it ranges between 1.62 and 2.40 mM.

The PGKs were the first glycolytic enzymes of *T. brucei* for which it was shown that their specific compartmentalization in glycosomes and cytosol has a key role in the proper execution of the parasite's energy metabolism and thus is essential for viability [298].

Whereas previous studies of PGK in *T. brucei* focused on the PGKA/B/C isoenzymes, recent genome analysis of this parasite by us and others revealed that it has an additional gene for a candidate PGK isoenzyme. This gene (Tb927.11.2380), located on chromosome 11, codes for a 101 kDa multidomain PGK-like protein (figure 5), which is constitutively expressed in the procyclic and bloodstream stages of the parasite and has a location in the flagellum, specifically in the axoneme. This flagellar PGK, designated PGKL, has an unusual structure with its PGK domain at the N-terminal side, followed by a cyclic nucleotide monophosphate-binding (CNB) homologous domain and a region matching a helix–turn–helix (HTH) domain of DNA-binding proteins (table 3). A distinctive feature of this PGKL is the apparent lack of catalytic PGK activity. Residues highly conserved in other PGKs and known to be involved in catalysis are absent, and its region homologous to DNA-binding proteins was predicted to have no longer DNA-binding ability [60]. Nonetheless, as will be discussed below (and is shown in table 3; electronic supplementary material, table SI), related PGKLs with CNB and HTH-like domains are also found in many other trypanosomatid species suggestive of a functional role. The combination of CNB and HTH domains is very common in transcription factors of Bacteria and Archaea, and has also been documented in eukaryotic proteins [299]. The HTH domain is frequently combined with a diverse set of catalytic domains, and several general functions are associated with such combinations. In proteins with also a catalytic module, the HTH domain functions as a substrate-recognition or localization domain and for recruitment of the enzyme to its site of action [299]. Sometimes, the combined HTH and enzymatic domains are used for feedback regulation of metabolic pathways, for example, when an HTH domain is linked to a domain of an enzyme catalysing a key step in a biosynthetic pathway to regulate the transcription of the genes for that pathway. This is what happens with the *E. coli* biotin operon repressor, BirA, which contains an N-terminal HTH domain fused to a C-terminal biotin ligase domain. In the presence of biotin, the enzymatic domain synthesizes the co-repressor, and the HTH domain represses the transcription of the biotin biosynthesis genes [300]. Other studies with this PGKL have shown that this isoenzyme may be necessary for ‘social motility’ during the *T. brucei* procyclic stage [301]. This term refers to group behaviour, in which parasites assemble into multicellular communities with emergent properties that are not evident in single cells [301]. Oberholzer *et al*. [302] and Lopez *et al*. [303] discovered that procyclic *T*. *brucei*, when cultured on semi-solid agarose, assemble into groups of cells that undergo collective movements, forming multicellular projections that radiate outward from the site of inoculation. Although individual parasites can move freely within a group, movement of the group is polarized such that it advances at a single, leading edge. Moreover, groups alter their movements when they sense other parasites nearby. This social motility requires sensing and responding to extracellular signals [302]. It may be regulated by a cAMP-dependent mechanism, in which cNMP-binding proteins (cNMP-BPs) are possibly involved [301,304,305]. PGKL is a candidate among the cNMP-BPs involved. Parasites in which PGKL gene expression was knocked down showed a slightly greater social motility than wild-type cells [301], although null mutants did not show morphological or growth phenotypes (see also §6.5) [60].

In *T. brucei,* social motility is probably regulated and depends on cAMP signalling systems in the parasite's flagellum. This flagellar cAMP signalling may control trypanosome progression through fly host tissues. cAMP-specific phosphodiesterases, localized in the flagellum and associated with social motility, are essential for virulence of bloodstream-form trypanosomes [306]. The social behaviour in this parasite seems to be important for differentiation and for the parasite to navigate through diverse environments encountered during their transmission and infection cycle [307,308].

8.2.3.2.2. *Trypanosoma brucei gambiense.* So far, PGK enzymes of *T. b. gambiense* have not been studied. However, in a preliminary analysis of the genome of strain *DAL972* of this subspecies that was originally isolated from a patient in West Africa, we identified genes for four different PGK isoenzymes (table 3 and figure 5). These enzymes have PGK-like characteristics very similar to those described above for the homologues in the non-human-infective subspecies *T. brucei brucei* 427 (the strain for which most of the biochemical and molecular biological studies related to PGK isoenzymes have been performed) [22,23,80,119]. Besides genes for the isoenzymes PGKA, B and C, each 99–100% identical to its orthologue in *T. brucei*, there is also one coding for a protein identical to *T. brucei*'s flagellar PGKL, described by Brown *et al*. [60], but the expression and localization of this latter protein in *T. b. gambiense* remain to be established (electronic supplementary material, table SI; figure 5).

8.2.3.2.3. *Trypanosoma congolense*. In the genome of *T. congolense*, responsible for African animal trypanosomiasis, six genes encoding PGK isoenzymes have been identified (table 3 and figure 5). Of these six *pgk* genes, three (TcIL3000_1_220.1; TcIL3000_1_230; TcIL3000_1_240) were already reported by Parker *et al*. [122]. These genes have a genomic organization in tandem similar to that in *T. brucei* and code for two identical cytosolic proteins, C1PGK and C2PGKC (45 kDa), and a glycosomal membrane-associated PGK (56 kDa), which are each 83% identical to *T. brucei* PGKB/C and A, respectively. These isoenzymes are constitutively expressed during the life cycle of *T. congolense*; however, ClPGK is most abundant in procyclic forms while C2PGK is more abundant in the highly glycolytic bloodstream and metacyclic forms. This expression pattern of *T. congolense* PGKs is reminiscent of the expression of the PGKA/PGKB/PGKC isoenzymes of *T. brucei*. With regard to the PGK activity, more than 90% was detected in the cytosol, corresponding to both C1PGK and C2PGK. Although the 56 kDa PGK is co-expressed with the other two isoenzymes, its activity could be shown to be associated with the glycosomes due to its larger molecular weight [122].

Metabolic differences between *T. brucei* and *T. congolense* have not only been observed in the expression of their PGK isoenzymes, but also in those of many other glycolytic enzymes. Comparative transcriptomic studies of *T. brucei* and *T. congolense* showed differences for components of several processes between both parasites. The expression of most of the enzymes involved in glycolysis was negatively regulated in the so-called stumpy form of *T. brucei*, the non-proliferative but tsetse fly-infective forms that accumulate in the blood at increasing parasite density, meanwhile no change between ascending and peak parasitaemia was seen for *T. congolense* [309].

Each of the isoenzymes has apparently a structure comprising only the PGK domain, except the 66 kDa protein encoded by TcIL3000.11.2180.1. This latter protein seems related to the 101 kDa PGKL of *T. brucei*. It has an incomplete PGK domain, lacking the 1,3BPGA binding site, but the residues involved in nucleotide binding and those that are part of the flexible hinge are well conserved. Like in *T. brucei* PGKL, the PGK domain is followed by CNB and HTH domains, but it differs from it (and the PGKL of some other *Trypanosoma* species—see below) by inversion of the order of these domains, suggesting that the *T. congolense* gene has undergone a recombination that changed its architecture. Moreover, the *T. congolense* protein lacks any transmembrane domains at the C-terminus (table 3). Genes for enzymes that participate in the glycolytic pathway are differentially expressed in *T. congolense,* being upregulated in parasites in the fly's proboscis. This is also the case for TcIL3000.11.2180.1. Differential expression of enzymes such as HK could be indicative of pre-adaptation of these parasites for the glucose-rich bloodstream environment in their mammalian host [310]; however, the significance of this expression pattern of TcIL3000.11.2180.1 is unclear, because this PGK isoenzyme is a catalytically inactive enzyme, or a ‘dead enzyme’. A group of enzymes encoded in the genomes of other kinetoplastids such as *L. major*, *T. cruzi* and *T. rangeli* have also been identified as ‘dead’ enzymes [3,311]. In metazoan organisms, such ‘dead’ enzymes were found to represent a source of biological regulators [312,313]. For example, kinases that have lost catalytic activity can exhibit biological activities via regulation of protein–protein interactions or allosteric modulation of the activity of enzymes. Additionally, a ‘dead’ enzyme can be involved in influencing the availability of substrate for other active enzymes, immobilization of the substrate to a particular subcellular space, or protein–DNA interactions [314,315]. The presence of an HTH domain, as in the protein encoded by TcIL3000.11.2180.1, points to some function in the regulation of gene expression. Proteins with an HTH type domain have been documented in many eukaryotes and bacteria. They participate in ribonucleoprotein complexes in the cytoplasm where they specifically recognize various classes of double-stranded RNAs [316].

Another distinctive feature in *T. congolense* is that 4 (TcIL3000_1_220.1; TcIL3000_1_230; TcIL3000_0_37920; TcIL3000_0_37930) of the 6 PGK genes appear to code for identical protein sequences. Two of these genes (TcIL3000_1_220.1; TcIL3000_1_230) are located on chromosome 1, while the chromosomal localization of the other two genes (TcIL3000_0_37920; TcIL3000_0_37930) is still unknown (figure 5). These genes must have resulted from recent duplication events in contrast with the other two genes (TcIL3000_1_240 and TcIL3000.11.2180.1). It is possible that natural selection has been acting on the retention of these gene duplicates, similar to that has been proposed for other eukaryotic organisms with many gene copies of enzymes that catalyse the same reaction [317].

8.2.3.2.4. *Trypanosoma evansi*. *T. evansi* is another parasite that causes animal trypanosomiasis, also outside Africa because its transmission is not dependent on tsetse flies, which are endogenous only to sub-Saharan Africa, but occurs mechanically. Studies of this parasite's metabolism involved glycolysis, including its enzymes like PGK. Rivero *et al*. [26] have shown that this parasite (strain TEVA 1), residing in the blood, has a more complex metabolism than has been postulated for bloodstream-form *T. brucei*. They showed that PGK activity occurs mainly within glycosomes, but it was not the only source for ATP synthesis within the organelles. Additional production of glycosomal ATP, necessary for other processess such as biosynthetic pathways compartmentalized in the glycosomes, was attributed to phosphoenolpyruvate carboxykinase (PEPCK) and pyruvate phosphate dikase (PPDK) [26]. Although there are so far no reports about the characterization of PGK isoenzymes in *T. evansi*, analysis of its genome showed that it has four *pgk* genes (table 3). Three genes code for the distinct PGKA/B/C isoenzymes (TevSTIB805.1.640; TevSTIB805.1.650; TevSTIB805.1.660, respectively), as in *T. brucei*. Two of them (TevSTIB805.1.640 and TevSTIB805.1.650) encode proteins that are 95% identical in sequence among them and to PGK isoenzymes B and C of *T. brucei*. This is in agreement with the notion that *T. evansi* evolved (independently, multiple times) from *T. brucei* after loss of critical parts of its mitochondrial DNA and can be considered as a subspecies of *T. brucei*; the two species exhibit overall a high identity of their genomes (approx. 95%) [318]. Through MS-based proteomics studies, more than 100 gene products, expressed by the parasite during infection in a rodent model, were identified [319]. Metabolic enzymes constitute approximately 23% of the identified proteins. Within that group of enzymes, the glycolytic enzymes were the most conspicuous, indicating their abundance and confirming the dependence of the parasite on this pathway as the main source of energy. Among the metabolic enzymes identified was PGK, along with other glycolytic enzymes (such as HK, GAPDH, PGAM, ALD, glucose-6-phosphate isomerase) as well as glycerol kinase. When performing an alignment of the 12 peptides obtained by the MS analysis from the isolated PGK protein [319] with the amino acid sequences predicted from the different *T. evansi* PGK genes we identified, 7/12 peptides appeared to match perfectly with the 45 kDa product of the TevSTIB805.1.650 gene (PGKB) and 12/12 peptides with the 47 kDa product (PGKC) encoded by the TevSTIB805.1.640 gene. In the case of the 56 kDa product (PGKA), only 3/12 peptides completely aligned with its sequence (these peptides also aligned with the sequences of the 45 and 47 kDa isoenzymes). However, none of the peptides aligned perfectly with the sequence of a 101 kDa PGK-like protein, encoded by the fourth gene that we also identified in our bioinformatics study and which is 100% identical to the *T. brucei* PGKL containing the CNB domain. Based on these analyses, we conclude that it is very likely that at least PGK isoenzymes of 47 and 45 kDa are expressed in the bloodstream form of *T. evansi*.

8.2.3.2.5. *Trypanosoma vivax*. *T. vivax* is another member of the African trypanosomes responsible for animal trypanosomiasis. However, it also occurs outside Africa because the parasite can be transmitted by different biting insects. *Trypanosoma vivax’* life cycle does not involve a procyclic stage in tsetse flies. When its bloodstream forms are ingested by an insect, they move directly to the proboscis, while differentiating via epimastigotes into metacyclic forms that can be introduced in new mammalian hosts. Only a few reports are available about PGK isoenzymes of *T. vivax*. Gene expression profiling through the complete, short life cycle of this parasite found that transcripts preferentially expressed in bloodstream forms are associated with glycolysis and glycosomes, indicating substrate-level phosphorylation as the dominant process for ATP generation in this stage, a consistent feature of bloodstream-form African trypanosomes. Within these enzymes that undergo a difference in expression and that are remarkably abundant in the bloodstream form are two PGK isoenzymes of 45 and 41 kDa (TvY486_0100140 and TvY486_0604100) [320] and table 3, 82/83% and 46% identical to *T. brucei* PGK isoenzymes B and C, respectively. However, the 41 kDa protein has undergone substitutions in its 3PGA binding site and is probably not active. Similar to the different subspecies of *T. brucei* (including *T. evansi*), an orthologue of PGKL was also identified in *T. vivax*, having 66% identity with the *T. brucei* protein, although with the lower molecular weight of 86 kDa due to the absence of an HTH-like domain (as also described above for the *B. saltans* and *P. confusum* PGKL) and shortening of membrane-spanning region at the C-terminal side. Additionally, a gene (TvY486_0100150) coding for an unusually small 32 kDa PGK is present in the genome of this protist. Despite its size, this enzyme has an apparently functional active site. An aspect that caught attention when analysing the genes for PGK isoenzymes was that the amino acid sequence of this 32 kDa protein contains a mitochondrial-type N-terminal targeting sequence. This mitochondrial PGK may have a non-canonical function in the organelle, for example, comparable with PGK's function as a PK as has been reported for mitochondria in some tumour cells [183]. Similar findings in other kinetoplastids will be described later in this review.

The analysis carried out in this work showed that *pgk* genes in at least some African trypanosomes are distributed in more than two loci. In these parasites, these genes are concentrated mainly on chromosomes 1, 6 and 11 (figure 3). Some of these parasites, such as *T. congolense*, have up to six candidate PGK genes. These genes code for a wide diversity of proteins. Some have a canonical PGK structure, while others are multi-domain, ‘dead’ PGKs or truncated PGKs (table 3 and figure 5). One of the most notorious aspects of these *pgk* genes is that they can be variable in number and structure between different species of African trypanosomes. Nevertheless, some of these PGK isoenzymes are highly conserved among different species.

##### 8.2.3.3. Stercorarian and reptilian trypanosomes

8.2.3.3.1. *Trypanosoma cruzi*. *T. cruzi* is one of the kinetoplastids of which PGK isoenzymes have been characterized in detail [4,24,25,124]. Until now, three genes encoding PGKs in *T. cruzi* have been reported. Two, *pgkA* and *pgkB/C*, are located in tandem on chromosome 9, while the third one, the *pas–pgk* gene, is found on chromosome 32 (table 3 and figure 5) [4,24]. However, as will be described in the next sections, bioinformatics analysis of its genome revealed that this parasite has at least 6 *pgk* genes, some of them encoding proteins with very peculiar characteristics (table 3).

Most studies of *T. cruzi* have focused on PGKA, B and C. Similar to the situation in *T. brucei*, these PGK isozymes of *T. cruzi* have a location within glycosomes (PGKA, 56 kDa/ PGKC, 47 kDa) and in the cytosol (PGKB, 45 kDa). These three isoenzymes are expressed simultaneously in the different life cycle stages of this parasite [24]. However, PGKC and PGKB expression is increased when glucose in the culture medium is limiting and amino acid consumption increases. This differential expression of these PGK isoenzymes is highly indicative of their participation in the change of energy and carbon metabolism in the parasite, specifically in a transition from glycolysis to gluconeogenesis [124].

The PGKA isoenzyme is characterized by having an insertion of approximately 80 amino acids in its N-terminal half and is tightly associated with the inner surface of the glycosomal membrane, although so far no region responsible for this association has been identified. The protein's amino acid sequence has 75%, 77% and 59% identity with the PGKAs from *T. brucei*, *T. congolense* and *Crithidia fasciculata*, respectively [24]. However, the 80 residues long insertion, specific for all PGKAs, is less conserved. The insertion of the *T. cruzi* enzyme shows, respectively, 57%, 54% and 52% identity with the corresponding peptide of the three other trypanosomatid PGKAs. An additional striking feature of the PGKAs of the different trypanosomatids is the calculated high isoelectric point of the insertion (between 9.6 and 10.5) [24]. Until now, little is known about the possible function of this insertion within the protein's structure. Kinetic studies demonstrated that its deletion has no effect on the catalytic activity of the *T. cruzi* PGKA. It is thought that the insertion could have some function in the formation of multienzyme complexes [25]. Although the nature of PGKA's intimate association with the glycosomal membrane has not yet been determined, results from different experiments indicated that this isoenzyme plays an important role in maintaining the glycolytic flux in *T. cruzi* [124].

*Trypanosoma cruzi* PGKB and C are essentially identical and share about 80% identity with their *T. brucei* orthologues [24]. The initial finding of only two PGK genes (one of them for PGKA) and the fact that PGKB and PGKC could barely be distinguished by western blotting, except for their different subcellular localization, have led to the following hypothesis [24]. First, that the PGK B and C isoenzymes are encoded by the same gene (*pgkb*: TcCLB.511419.40), and second, that the product of this single gene has a dual location, in the cytosol and in the glycosomes [124]. The protein predicted from the *pgkb* gene does not have an apparent PTS sequence. A routing signal might be encrypted internally in the sequence of the protein and would be responsible for the partial compartmentalization of the enzyme within the glycosomes. Additionally, the slight differences between PGKB and PGKC as detected on western blots may be due to covalent posttranslational modification of the protein, that in turn then also contributes to the regulation of the PGKB and PGKC levels in their respective compartments in response to the available carbon source. Such PTM may also influence the catalytic activity of the PGKC isoenzyme, as will be discussed below [25].

The activity of PGKB accounts for approximately 80% of the total activity and is cytosolic, whereas PGKA and PGKC are together responsible for an estimated 20% of the total cellular activity [24]. PGKA is characterized by low catalytic activity; the 20% glycosomal PGK activity is for 23% attributed to this enzyme, while 77% corresponds to PGKC activity. Kinetic studies of recombinant PGKA yielded Km values for 3PGA and ATP of 850 and 236 µM, respectively [25,124]. On the other hand, the PGKB and PGKC isoenzymes, despite having very similar sequences and molecular weight, have very different affinities for both substrates. PGKB's Km values for 3PGA and ATP are 625 µM and 99 µM, respectively (W. Q. *et al*. 2006, unpublished results), with the Km value for ATP being 2.4 times lower than that reported for the recombinant PGKA of this parasite. The PGKC is partially inhibited by ATP at concentrations higher than 130 µM, showing a Ki value (ATP) of 270 µM. The Km for ATP was 10 µM, an order of magnitude below that of PGKA and PGB. However, the Km of PGKC for 3PGA was 192 µM, similar to that reported for isoenzymes PGKA and PGKB. This was the first report of a PGK enzyme inhibited by ATP [25]. From these studies, we have tried to understand the possible function of each of these isoenzymes and the physiological significance of their simultaneous expression during all developmental stages of *T. cruzi* studied so far. Data suggest that PGKA is responsible for the glycolytic reaction of converting 1,3BPGA to 3PGA, with concomitant formation of ATP, within the glycosomes. On the other hand, the inhibition of PGKC by ATP and its high affinity for this substrate would suggest that this enzyme works in the gluconeogenic direction, supporting this anabolic pathway through glycosomes even when the ATP/ADP ratio within the organelles is very low. Finally, the cytosolic PGKB may rather exert a glycolytic function [25,124].

Recently, we identified and characterized also the product of a gene, TcCLB.506945.20, that codes for a PAS–PGK in the *T. cruzi* strain CL-Brener Esmeraldo like. A PAS–PGK protein is also present in the genome of all other *T. cruzi* strains analysed (electronic supplementary material, table SI), and other kinetoplastids (table 3), except African trypanosomes [4,321]. The *T. cruzi pas–pgk* gene is on a different chromosome than the *pgkA*–*pgkB/C* gene array (figure 5). The encoded protein has a molecular weight of 58 kDa, exhibits 44–45% identity to PGKA and PGKB and has all residues involved in PGK substrates binding and catalysis (W. Q. *et al*. 2018, unpublished results). Structurally, this PAS–PGK is a multidomain protein with, in addition to a PGK domain, a PAS domain at its N-terminal end [4]. Like the other PGK isoenzymes of *T. cruzi*, this PAS–PGK is expressed throughout the life cycle of the parasite [94].

The PAS–PGKs of *T. cruzi* and other trypanosomatids possess a PTS1, in the case of the *T. cruzi* PAS–PGK the C-terminal sequence -PRL [4]. Indeed, proteomic analysis of purified *T. cruzi* glycosomes has shown that this protein is present in glycosomes where it appears to be peripherally associated with the membrane. Like the other PGK isoenzymes, PAS–PGK is expressed during all phases of epimastigotes (life cycle stage in the triatomine insect vector) growth in culture; however, it is more abundant in the proteome of glycosomes of epimastigotes harvested in the stationary growth phase than in exponentially growing cells [104]. PAS domains are known to have a site with the ability to bind a wide diversity of ligands (FMN, FAD, haem, chromophores and fatty acids) but they can also function in signalling without binding a small molecule, through the dimerisation of PAS domains. The PAS domain of *T. cruzi* PAS–PGK contains amino acids predicted to be involved in the formation of a similar ligand-binding site as the PAS domain of other proteins from different organisms [4]. It is noteworthy that the *pas–pgk* gene in the genome of the *T. cruzi* CCC strain has apparently undergone a duplication. The products of both sibling genes are predicted to contain a catalytically active PGK domain (electronic supplementary material, table SI). Duplication of PAS–PGK genes has not been detected in any other *T. cruzi* strains or kinetoplastid species we analysed. The absence of this PAS–PGK in African trypanosomes (such as *T. brucei*), reported by us in previous studies [4], was also reported by Durrani *et al*. [321].

In *T. cruzi*, three *pgk*-related genes were identified in addition to those already previously documented (table 3). One of these genes, TcCLB.504153.20, codes for a 95 kDa PGK similar to the PGKL reported in *T. brucei,* a multidomain protein with PGK and CNB domains, but like *T. vivax* and *B. saltans* lacking a separate HTH domain. Target prediction studies suggest that this protein may be located at the glycosomal membrane (result not shown). Moreover, it has a pI value around 9, even higher than the PGKL of *T. brucei*. A high pI is characteristic of many glycosomal proteins [322]. Additionally, a gene (TcCLB.506127.9) encoding a small-sized (13 kDa) protein was found in the *T. cruzi* genome that was identical to the N-terminal part of the PGKL, indicating that it must have resulted from a partial gene duplication, or a gene duplication and subsequent truncation. Finally, the TcCLB.506125.30 gene codes for a fragment of a possible PGK protein. All residues involved in catalysis were completely lost; the remaining amino acids cover three segments of a transmembrane domain, showing 93% identity with the C-terminal end of the PGKL, thus also pointing to a partial duplication of the TcCLB.504153.20 gene. These last two ‘*pgk*-like’ genes probably do not code for functional proteins but are evolutionarily recent genomic junk.

The analysis of the *T. cruzi* genome not only revealed the existence of PGK isoenzymes and ORFs for potential PGK-related proteins additional to those already previously described for this parasite, but also that the number and nature of these possible PGK isoenzymes and related proteins may vary among different strains (electronic supplementary material, table SI). In some strains such as *Sylvio X10/1–202*, the number of ‘dead’ PGK isoenzymes exceeds that of catalytically active isoenzymes. On the other hand, analysis of the genome of the *T. cruzi* CCC strain revealed that its *pas–pgk* gene underwent a duplication process (electronic supplementary material, table SI). Together, these findings may be indicative of the high ‘fluidity’ of the genome of these organisms, resulting in frequent (partial) gene duplications and subsequent rapid evolution of redundant genes. Another important fact of this analysis is that, among all kinetoplastids analysed up to this point, *T. cruzi* is the one with the highest number of PGK isoenzymes located and expressed simultaneously in the glycosomes.

8.2.3.3.2. *Trypanosoma rangeli*. In *T. rangeli,* a protist closely related to *T. cruzi*, some *pgk* genes (and their products) have also been characterized. Our genome analysis of this kinetoplastid revealed four *pgk* genes (table 3), a result similar to that reported by Villafraz *et al*. [27]. The product of the TRSC58_02767 gene is a 45 kDa protein, lacking a typical PTS and indeed detected in *T. rangeli* epimastigotes. Its amino acid sequence has 85.7% identity with the cytosolic PGKB of *T. cruzi* [27]. The products of the TRSC58_00037 and TRSC58_04456 genes probably correspond to glycosomal proteins [27] (table 3). TRSC58_00037 codes for a multidomain 95 kDa PGK-like protein similar to the PGKL (with domains: PGK-CNB-HTH-TMD; table 3) reported for several African trypanosomes (*T. brucei* TREU927, *T. b. gambiense* and *T. evansi*) and also found in *Crithidia* (table 3). As in other trypanosomatids, this protein is predicted to be located in the membrane of glycosomes, except for the procyclic form *T. brucei*, where it was shown to be flagellar [60]. Regarding the product encoded by the TRSC58_00739 gene, previous studies have shown that this PGK is very similar to the 56 kDa PGKA of *T. cruzi*, with 82.1% sequence identity [27]. The similarity between these sequences has led us to propose that this enzyme probably has a location in the glycosomes [27], although its sequence does not have a PTS1 motif. The TRSC58_04456 gene codes for a PAS–PGK, similar to that reported in previous studies of *T. cruzi* [4]. It shares 86% sequence identity with *T. cruzi* PAS-PGK. In addition to having a PAS domain in the N-terminal half, it contains a C-terminal tripeptide PKL (as its counterpart in *T. cruzi*), a PTS1, suggesting a glycosomal location of the protein [4,27].

The genomic organization of some of these genes in *T. rangeli* is similar to that reported for other trypanosomatids. The TRSC58_00739 and TRSC58_02767 genes are linked, while the other two genes, TRSC58_00037 and TRSC58_04456, are apparently located elsewhere in the genome [27]. This organization is reminiscent of that in *T. cruzi*, where the PGKA gene is followed by a PGKB/C gene. However, with regard to the subcellular localization of the expressed proteins, a difference was found between epimastigotes of the two species. PGKB of *T. rangeli* (product of the TRSC58_02767 gene) has an exclusively cytosolic location [323]. No PGK activity was detected in any other compartment than the cytosol [323], although the possibility that the products of genes TRSC58_00739 and TRSC58_04456 have also negligible activity in the glycosomes cannot be discarded, since the residues involved in substrate binding, catalysis and flexible hinge structure are highly conserved [27].

Regarding the kinetic characteristics of the PGKB of this parasite, both the natural and recombinant enzymes displayed standard Michaelis–Menten kinetics. The Km values for ATP are 0.13 mM and 0.5 mM, and for 3PGA 0.28 mM and 0.71 mM, in each case for the natural and recombinant enzyme, respectively (table 2) [27]. These values are comparable with previously reported Km values for the PGKB isoenzyme of *T. brucei* (Km^ATP^ 0.46 mM, Km^3PGA^ 2.04 mM) [69] and *T. cruzi* (Km^ATP^ 0.09 mM, Km^3PGA^ 0.62 mM; W. Q. *et al*. 2006, unpublished results). The similarity of these values reflects the high level of amino acids conservation in the active site between the PGKB of *T. brucei*, *T. cruzi* and this PGKB of *T. rangeli* [27]. The results obtained so far suggest the presence of a single PGK in the cytosol of the *T. rangeli* epimastigotes that acts in glycolysis when glucose is available in the environment. However, when this monosaccharide or other nutrients that may enter the glycolytic pathway is limited or absent in the vector insect, this PGK should participate in gluconeogenesis, when the parasite consumes lipids and/or amino acids [27,323].

8.2.3.3.3. *Trypanosoma grayi*. *T. grayi* is an African crocodile-infecting trypanosome; however, it is evolutionarily closer to *T*. *cruzi*, *T*. *c*. *marinkellei* and *T*. *rangeli* than to African trypanosomes [324,325]. Little is known about the metabolism of this trypanosome, but its genome has been sequenced. Like for other trypanosome species, several *pgk* genes are present in the *T. grayi* genome (table 3). The DQ04_00061260 and DQ04_02181000 genes apparently code for multidomain PGK-like proteins, while the DQ04_04781080 gene codes for a PGK protein consisting of a single domain.

Also a gene encoding a truncated PGK protein (DQ04_13681000) was observed in this trypanosome. It only retains the amino acids involved in the 3PGA substrate binding, but has completely lost key amino acids to carry out other functions such as catalysis and nucleotide binding, similar to what has been observed in some other trypanosomatids (see above and table 3). *Trypanosoma grayi* has a 58 kDa PAS-PGK (DQ04_00061260), like *T. cruzi*, with a possible location in the glycosomes, where it seems to be the only PGK isoenzyme. The multidomain PGK encoded by the DQ04_02181000 gene is characterized by having also CNB and HTH domains, but not a transmembrane domain. Sequence analysis of this 80 kDa protein suggests that it is located in the cytosol, and lacks amino acids involved in substrate binding, thus it could be considered a catalytically ‘dead’ PGK enzyme, although it may exert other functions as discussed for PGKLs elsewhere in this paper (§8.2.3.2.1). By contrast, the 48 kDa PGK, encoded by DQ04_02181000, has all characteristics of a fully active enzyme and apparently has a location in the cytosol.

It is very interesting that half (2 of 4) of the genes in *T. grayi* discussed here are predicted to code for proteins that are probably catalytically inactive PGKs. This characteristic seems to be shared with *T. cruzi* and *T. congolense*. For several of these ORFs, information about their expression is still lacking. In some cases, the ORFs are clearly derived from partial gene duplications and can almost certainly be considered as silent, non-functional DNA. For some other ORFs, it is feasible that proteins are produced that exert other physiological functions than catalysing the PGK reaction. This aspect will be discussed in more detail, in §9.

##### 8.2.3.4. *Angomonas* and *Strigomonas*

*Angomonas deanei* belongs to the trypanosomatid group of monoxenous parasites, including also *Crithidia*, *Blastocrithidia*, *Strigomonas*, *Herpetomonas* and *Leptomonas*, genera of organisms that together infect a wide range of insects [326,327]. *Angomonas*, along with the genera *Strigomonas* and *Kentomonas*, form a monophyletic clade within the Kinetoplastea, the subfamily Strigomonadinae (figures 1 and 2), that is characterized by the presence of a single β-proteobacterial endosymbiont in their cytoplasm [328]. Typically, there is only a single bacterium per trypanosomatid cell (*Candidatus* Kinetoplastibacterium or TPE (trypanosomatid proteobacterial endosymbiont)) that divides synchronously with the host protist; thus, at the end of the mitotic process, each daughter cell contains a single endosymbiotic microorganism [329].

Both organisms, the trypanosomatid and its endosymbiont, display a sophisticated interrelationship, in which the host is supplied with essential compounds including haem, nucleotides and essential amino acids. The endosymbiotic *Ca.* Kinetoplastibacterium spp. preferentially retain those genes necessary for the biosynthesis of compounds needed by their hosts [329,330]. Strigomonadinae can grow in defined media lacking haem and containing a reduced number of amino acids because many metabolites, important for the trypanosomatid's growth, can be synthesized by the endosymbiont and delivered to its host [330,331]. Besides the metabolic contribution, the endosymbiont affects the morphology and ultrastructure of the host cells [329,332,333]. Ultrastructural studies of *A. deanei* and *Strigomonas culicis* showed that many glycosomes are located in close association with the endosymbiont [333,334]*.* Based on studies of *A. deanei*, Motta *et al*. [333] postulated that ATP produced in the protist is used by the endosymbiont, since the bacterium's respiratory chain seemed to be inactive due to the absence of activity of cytochrome aa_3_ oxidase and succinate dehydrogenase (nonetheless, it consumes O_2_ once isolated from the host [335]). The ATP produced by the host cell can possibly be hydrolysed by the symbiont's ecto-ATPase, which may support an active transport system in the prokaryotic cell envelope [334]. The observation that different endosymbionts in protists such as *A. deanei* and *S. culicis* maintain a close association with glycosomes suggests that metabolic products synthesized in these organelles are essential for the physiological maintenance of the bacterium and the symbiotic association. Besides ATP, other phosphorylated intermediates generated by the glycolytic pathway of the host, such as the 3PGA produced by PGK could be used by the endosymbiont [334].

A search in the database revealed in *A. deanei* 12 genes potentially coding for PGKs. Three of these PGKs share the characteristic length of 502 amino acids and are potentially active, because they seem to have all residues required for substrate binding and catalysis. Another PGK with 418 residues is also potentially active (table 3). None of these four PGKs has a consensus PTS, suggesting that these enzymes are located in the cytosol of *A. deanei*. A PGK with a PAS-domain with 526 amino acids was also detected, it could be active and located in the glycosomes, since it has at its C-terminus a PTS1 sequence (-PKL), which previously has been shown functional in targeting this protein to the glycosomes of *T. cruzi* and *Leishmania major* [28,104]. Seven other genes that code for PGK-like sequences are predicted to be inactive, because they lack residues involved in catalysis or substrate binding. One of them is a PGK of 270 amino acids; the other six, between 310 and 924 residues long, share the presence of between one to three transmembrane (TM) segments. Three of these latter proteins have also a CNB domain (but no HTH domain) located between the PGK and the TM domain (table 3). All of these 12 genes belong to *A. deanei*. A PGK gene of *Ca.* Kinetoplastibacterium spp. was also identified (WP_015238965.1), coding for a product with a molecular weight of around 40 kDa but predicted to be inactive.

A similar situation was found for *S. culicis* that has at least 10 genes coding for PGKs, seven of them for active PGKs. Three of these PGKs with length of 417, 455, and 500 amino acids are probably in the cytosol and we hypothesize that the protein of 455 residues with a TM is associated with the glycosomal membrane. A PAS–PGK of 527 amino acid possesses a PTS1 (-PKL) and thus will most likely be located in the glycosomes. Three proteins of 935 residues with three different domains, PGK-CNB-TMD, have been identified; two of them (STCU_08802; STCU_06143) are, in contrast with their orthologues in *A. deanei*, predicted to be active, while the inactive (STCU_02304) protein has one additional TM segment. Among the other inactive PGKs found is one of 270 residues, and another one of 458 amino acids with a CNB domain included (table 3). Proteomics analysis on wild-type *S. culicis* (with the endosymbiont) and a corresponding aposymbiotic (Apo) strain (i.e. without endosymbiont) showed that throughout growth in batch culture, the PGK, PAS-PGK (STCU_04975) and PGKc (STCU_01151) are constitutively expressed at similar levels in both strains [336].

Five different PGK sequences belonging to the endosymbiont (*Ca.* Kinetoplastibacterium blastocrithidii) were found. However, an alignment of these sequences showed that all of them code for the protein identified with the sequence WP_015237848.1 (table 3).

From this analysis of PGK sequences reported for *A. deanei* and *S. culicis*, we infer that the 3PGA can be generated within glycosomes as well as in the cytosol of these endosymbiont-containing hosts, in a similar way as other trypanosomatids like *T. cruzi* which contain PGKs located in the glycosomes and cytosol.

##### 8.2.3.5. Leishmania

Different studies of PGK isoenzymes have been carried out for *Leishmania* spp. [28,81,279,281,337,338]. These studies have shown that these parasites have two tandemly linked PGK genes on chromosome 20 (figure 5), corresponding to the genes *pgk*B and *pgk*C in *Trypanosoma* and *Crithidia*. These genes code for a 45 and 51 kDa protein, respectively (table 3). The two polypeptides are 99.5% identical in their sequences. As in other trypanosomatids, the PGKB isoenzyme is in the cytosol, while PGKC is compartmentalized in glycosomes [81,279,281]. The isoenzyme PGKC is characterized by having a long C-terminal extension of 63 amino acids that is unique to PGKCs of the genus *Leishmania* [81,281,338]. This extension, being the only difference between PGKB and PGKC besides two residues subsititutions elsewhere in the proteins, is most likely responsible for routing the isoenzyme to the glycosomes. No PTS1 is present in its C-terminal sequence, but an internal sequence acting as glycosomal import signal has not been ruled out. A notable feature of this 63 residues long C-terminal extension is that it has alternating stretches of hydrophobic and charged, mainly positive amino acids. These characteristics may be involved in a protein–protein interaction, allowing the association of the PGKC with other soluble or membrane-associated glycosomal proteins. Additionally, it could serve as a binding site for some ligand, allowing the modulation of the activity of this isoenzyme [281]. On the other hand, theoretical and *in vitro* studies of the PGKC of *L. mexicana* by Raghunathan and co-workers [68] demonstrated that this extension has a propensity to form a transmembrane helix located between residues 451–472, ending in a cluster of two Arg residues (R471/R472). Therefore, there is also a possibility that the extension serves to anchor the PGKC to the glycosomal membrane. This idea of a membrane association could be supported by results obtained by Quiñones *et al*. [339], who observed a 56 kDa protein that is not an integral membrane protein, but seems firmly attached to the inner face of the glycosomal membrane or is a peripheral component of a membrane protein complex. Based on further theoretical considerations, Raghunathan and co-workers [338] proposed that the C-terminal extension may form a metabolite or ion transporter within the glycosomal membrane. However, *in vitro* or *in vivo* experimental studies supporting this notion are lacking. Although the 63 residues C-terminal extension is present in all *Leishmania* species analysed, with the sequence being conserved at 65–96% identity pointing to a shared function, it is important to consider that such a long extension is not common in the orthologues of trypanosomatids; it is only found also in the *Leptomonas* spp. glycosomal PGK (with 40–53% sequence identity to the *L. mexicana* PGKC extension), whereas in all other trypanosomatids studied, it is much shorter or even absent (e.g. in *Crithidia* 38, *T. brucei* 20 and *T. cruzi* 0 residues). Moreover, there is strong evidence that in all peroxisomal, including glycosomal membranes non-selective channels are present that allow the translocation of ions and metabolites with molecular weight up to 300–400 Da [340], rendering unlikely the possibility of an additional, PGK-coupled transport system only in *Leishmania*.

One aspect of the *pgk* genes in which *Leishmania* differs from other trypanosomatids studied, (i.e. *T. brucei*, *T. congolense*, *C. fasciculata* and *T. cruzi*) is the absence of a third PGK gene in a tandem array, a *pgk A* gene coding for a functional PGK with a long insertion in its N-terminal half [81,279,281]. The consequence of this absence is still unknown; however, different explanations have been proposed. *Leishmania* is less dependent on glycolysis than other trypanosomatids such as *T. brucei*; previous studies with axenic amastigotes of *Leishmania* spp. and amastigotes in *in vitro* infected (non-activated) macrophages showed that the glucose utilization is markedly lower than fatty-acid or amino acid oxidation [341,342]. However, recent research demonstrated that amastigotes in activated macrophages and in animal models rely on glycolysis [343].

Although the PGK B and C enzymes of *L. mexicana* are very similar to their developmentally expressed B and C orthologues in *T. brucei* (to which they have around 73% identity for both isoenzymes), the expression and activity attributed to each of the enzymes are rather like those of the isoenzymes of *T. cruzi* (to which the identities are approx. 76%). Both isoenzymes are also expressed simultaneously in *Leishmania*, with 80% of the PGK activity found in the cytosol and 20% in the glycosomes of cultured *L. mexicana* promastigotes [281]. This difference is related to the differences in expression levels of these isoenzymes, with the transcripts and proteins of PGKB and PGKC both being expressed at a ratio of approximately 4 : 1. The protein levels are maintained as a result of multiple levels of control, both posttranscriptional and posttranslational [279]. Regarding the function of these enzymes, the 80/20 expression of the PGK isoenzymes could reflect how fluxes have to be routed through the different branches of carbon metabolic network that is distributed over the glycosomal and cytosolic compartments (the glucose catabolism to pyruvate or via PEPCK and malate dehydrogenase to succinate, and gluconeogenesis to glucose 6-phosphate), while the ATP consumption and production and NAD^+^/NADH redox level within the glycosomes have to be kept in balance [281,344].

As for *T. cruzi*, a PAS–PGK, encoded by the LmjF.30.3380 gene, was recently described for *L. major* (LmPASPGK) [28], and is also present in other *Leishmania* species (electronic supplementary material, table SI). This enzyme has a molecular weight (58 kDa) and domain structure that are very similar to those of the *T. cruzi* PAS–PGK [4]; table 3). Little information is known about a possible ligand to its PAS domain. Its PGK activity appeared to be insensitive to FAD, FMN, 4-hydroxycinnamic acid and fatty acids. However, association of this enzyme with haem in *in vitro* assays increased its activity by 20%. Regulation of the activity is a distinctive feature of this LmPAS-PGK. It has maximal activity at pH 5.5 that decreased towards a pH close to 7.5. This effect of pH on PGK activity occurs indirectly through the PAS domain. Kinetic studies also demonstrated that the absence of the PAS domain has no effect on the affinity of the enzyme for its substrates (ATP/3PGA) [28]. It may be relevant to note that the Km values of the LmPAS-PGK for its substrates (118 µM to ATP and 661 µM) are very similar to those of PGKB of *T. cruzi* (W. Q. *et al*. 2006, unpublished results; table 2). Interestingly, a dual subcellular location has been reported for this *Leishmania* PGK isoenzyme, in glycosomes and lysosomes. The enzyme contains a PTS1 (the tripeptide -PKL) for import into glycosomes and seems to have, in that same C-terminal region before the glycosomal sorting signal, a post-Golgi-sorting motif (FLELL), which is probably responsible for its translocation to lysosomes [28]. This putative lysosomal translocation signal in LmPAS-PGK is very similar to the known dileucine-based sorting signals identified in lysosomal proteins [345,346] and appears to be conserved in the PAS-PGKs of other trypanosomatids. The lysosomal localization of LmPAS-PGK is intriguing and possibly provides a rationale for why the pH optimum for PGK activity at 5.5 has developed, a pH unlikely to be encountered within glycosomes [340], although the availability of PGK substrates (3PGA/1,3BPGA and ADP/ATP) in lysosomes and how this activity relates to a physiological role of the enzyme in these organelles remain to be established. Adhikari *et al*. [28] suggested that this LmPAS-PGK may exert different functions in the survival of the parasites, including regulation of the glycolytic flux and autophagosome formation in promastigotes, and probably has also a role in disease development in macrophages or mice. LmPAS-PGK, like other PAS proteins, seems to be an enzyme that links different cellular processes, similar to what we previously proposed for TcPAS-PGK [4]. This would not be the first report of a PAS protein with a possible function in autophagy. Nuclear receptor coactivator 4 (NCOA4) is a transcriptional co-regulatory protein that contains a basic helix–loop–helix domain, a PAS domain and seven LXXLL motifs [347]. The main function attributed to NCOA4 is to interact with several activated nuclear transcription factors, including the aryl hydrocarbon receptor and the androgen receptor [348]. However, NCOA4 can fulfil also other previously unknown functions, such as apparently acting as an autophagy cargo receptor [349].

Like in other kinetoplastids discussed in this review, a pseudogene encoding a part of a PGK-like protein was identified in *L. major* (table 3 and figure 5). However, the predicted protein is very peculiar. The software used for its sequence analysis predicted it to be a bifunctional PGK–TIM enzyme, having a part of a TIM-like protein fused at the C-terminal side of the PGK fragment. Bifunctional PGK–TIM enzymes have previously been identified in thermophilic bacteria, such as *Thermotoga maritima* [84]. In *T. maritima*, the 70 kDa PGK–TIM fusion protein is an elaborate bifunctional ‘polyprotein’ that carries both PGK and TIM activity. Unlike canonical PGKs, this PGK–TIM polyprotein forms a tetrameric complex that only exhibits significant catalytic activity at high temperatures [84]. Also *T. neapolitana* produces PGK as both an individual enzyme and a PGK–TIM fusion protein, but unlike *T. maritima*, which forms only one PGK–TIM fusion protein, *T. neapolitana* expresses two additional PGK–TIM fusion proteins, of 73 and 81 kDa [350]. A possible advantage for a cell to combine PGK and TIM in a single protein is enigmatic, because the two enzymes catalyse reactions which do not represent consecutive steps in the glycolytic pathway. Bifunctional enzymes usually catalyse two reactions involving a common metabolite. Such enzymes can also be found in parasitic protists like trypanosomatids. One example is the bifunctional enzyme dihydrofolate reductase/thymidylate synthase (DHFR-TS), encoded by a single copy gene (*dhfr-ts*), that plays a role in the reduction of folate, a key cofactor for DNA synthesis [351–353]. It has an N-terminal DHFR domain and a C-terminal TS domain, with the two domains separated by a junction peptide of variable size. Two sequential catalytic activities are contained on a single polypeptide chain, and the metabolite is channelled between both catalytic sites. Another, different kind of example is the 6-phosphofructo-2-kinase/fructose-2,6-bisphosphatase, in which the two linked enzymes are, respectively, responsible for the synthesis and hydrolysis of the potent glycolysis allosteric regulator fructose 2,6-bisphosphate [354]. However, whether PGK, or any other glycolytic enzyme is involved in the formation of such a bifunctional enzyme in kinetoplastid protists would require further studies.

##### 8.2.3.6. Leptomonas

In the monoxenous insect parasite *Leptomonas pyrrhocoris*, the presence of *pgk* genes is similar to that observed in other trypanosomatids analysed so far; its genome contains several genes encoding PGK isoenzymes. Two of these genes are arranged in tandem on chromosome 25 and the other two are located on chromosomes 4 and 7 (figure 5). These isoenzymes have molecular weights ranging from 51 and 101 kDa (table 3). Two of these PGK isoenzymes are part of multidomain proteins, with the PGK domain being associated with a CNB domain (in LpyrH10_07_2090) and a PAS domain (in LpyrH10_04_6440). The other two (LpyrH10_25_1860 and LpyrH10_25_1870), which are approximately 56% identical, are structurally canonical PGKs (table 3). It is important to highlight that analysis of the sequences of these latter two proteins revealed for both a probability for a glycosomal localization, despite the absence of consensus PTS motifs, as well as a possible localization in the mitochondrion, in agreement with the presence of mitochondrial-like amino-terminal targeting sequences in these isoenzymes (results not shown). Although there is no experimental evidence yet for the presence of glycosomes in *Leptomonas*, previous genomic analysis suggested that the initial part of the glycolytic pathway (from HK to PGK) as well as several other enzymes such as PPDK, which are commonly found in glycosomes of trypanosomatids, are similarly compartmentalized in *Leptomonas* [288]. In addition, genes for Krebs cycle enzymes and all the subunits of the pyruvate dehydrogenase complex were identified, also indicating the presence a functional mitochondrion in *L. pyrrhocoris* [288]. Based on a possible mitochondrial localization of some PGK isoenzymes, it would be valid to wonder what the meaning of the presence of these enzymes in the mitochondrion of *L. pyrrhocoris* could be. There are reports of tumour cells and protists for which the presence of a PGK in mitochondria has been documented [183,273]. As for *T. vivax*, we suggest that these isoenzymes may have a non-canonical function or an additional one to their canonical one.

Although the number of reports related to sugar and polysaccharide metabolism in *L. pyrrhocoris* is limited, this protist can use a wide variety of molecules as carbon sources. Based on genome analysis, this protist is predicted to have the ability to consume hexose sugars (glucose, fructose, mannose and galactose), pentose sugars (ribose, xylulose and ribulose) [288] and polysaccharides such as chitin, the latter being abundant in insects [355]. Additionally, this parasite can use some amino acids such as proline and glutamate as carbon sources, which are also abundant in the intestine of insects [356]. Although this organism is classified as monoxenous, variations in the carbon sources provided by its host are possible.

It is important to highlight that duplication of *pgk* genes in *Leishmania* and *Leptomonas* reported in this work is in accordance with the finding by Durrani *et al*. [321] that these organisms are prone to polyploidy, a feature that they reported in particular for genes of glycosomal matrix proteins.

##### 8.2.3.7. Crithidia

Like in several other kinetoplastids, PGK has been also experimentally characterized in *C. fasciculata* [80,125]. These studies have shown that *Crithidia*, like *T. brucei*, contains three *pgk* genes, A, B and C, in a tandem array located on chromosome 18 [125] (figure 5). Genes B and C encode a cytosolic (PGKB, 45 kDa) and glycosomal (PGKC, 48 kDa) isoenzyme, respectively. The *pgkA* gene has an ORF for a 56 kDa PGK, which has most active site residues conserved and is characterized by having an insert of 80 amino acids. This insert sequence is conserved between *T. brucei* and *C. fasciculata* with 48% positional identity [80], even though the lineages to each of these parasites have separated by almost 300 Myr [357]. The results of our bioinformatics analysis for this review for the three genes and their expressed products, as catalogued in table 3, are in full agreement with those from the previous studies by Swinkels *et al*. [80,125].

No biochemical studies have yet been carried out of the *Crithidia* PGK isoenzymes. However, proteomics analysis has shown that the glycolytic pathway is highly expressed during the early and mid-logarithmic growth phase of *in vitro* cultured choanomastigotes of *C. fasciculata* [358]. During these growth phases, various glycolytic enzymes (ALD, HK, PYK) increase their expression importantly. This could indicate that a high activity of the glycolytic pathway at mid-logarithmic phase is required to provide energy and essential precursors of certain amino acids, ribonucleotides and derived coenzymes. PGK and other enzymes such as TIM, that catalyse reactions that under physiological conditions are easily reversible, are constitutively expressed, unlike the others mentioned above [358]. Furthermore, like other trypanosomatids analysed in this work, genes that code for multidomain PGKs (i.e. a PAS–PGK and a PGK with a CNB domain) were also identified in *Crithidia*. Interestingly, the orthologue of PAS–PGK was shown to be upregulated at the transcript level in amastigotes compared to stationary phase promastigotes [359]. As observed for *T. cruzi*, *Leishmania*, *Leptomonas* and *Crithidia*, the number and nature of PGK isoenzymes can vary within different isolates. However, in the case of these kinetoplastids, the presence of ‘dead’ PGK enzymes is less common (electronic supplementary material, table SI).

##### 8.2.3.8 Summary of the functions of kinetoplastid PGKs

The differential expression of PGK isoenzymes present in kinetoplastids seems to be related to adaptation of these (free-living and parasitic) organisms to changing environmental conditions. When analysing the genomes of different kinetoplastid species, we found several important data about this glycolytic/gluconeogenic enzyme. (i) The number of genes (potentially) encoding PGK isoenzymes and PGK-related proteins (‘dead’ PGKs) can vary greatly in these protists, from 1 (in *Perkinsela*) to 6 (in *T. congolense* and *T. cruzi*). (ii) Some of these PGKs have additional domains such as a PAS, CNB and/or some transmembrane regions of which the location within the overall protein structure may vary. The two former modules are known to be responsible for regulation of a wide diversity of cellular processes (from metabolite transport to differentiation) in cells. (iii) (Pseudo)genes for one to several catalytically ‘dead’ PGK isoenzymes are present in some kinetoplastids. This lack of catalytic activity is most often due to the absence of the binding site for 3PGA/1,3BPGA or ADP/ATP, either resulting from residue substitutions within these sites or from a major deletion or truncation, or in some other cases only due to the mutation of an essential catalytic residue. PGK isoenzyme orthologues may be present in different kinetoplastid species; however, they may, according to sequence-based predictions, have different subcellular location sites. Additionally, in some species, ‘dead’ PGK enzymes have been shown to be expressed where they can represent up to 70% of the total PGK-related protein content. (iv) In the case of *Leishmania*, a pseudogene for a short protein was detected that, according to the prediction by the software used, combined stretches of PGK and TIM-related sequences. However, based on levels of similarity, structural organization of the ORF and the exclusive finding of the pseudogene in species from only one genus, one may wonder if it is a vestige of what once has been a gene for a bifunctional PGK–TIM. Further studies may be required to address this issue. (v) Some of these isoenzymes, such as PAS-PGK, are totally absent in African trypanosomes. (vi) The number of genes for PGKs and PGK-related proteins can vary significantly between strains of a species, at least for *T. cruzi*, *Leishmania* and *Leptomonas*. (vii) PGKs and PGK-related proteins can have different (experimentally determined or sequence-based predicted) subcellular locations, such as in glycosomes, mitochondrion, flagellum, lysosomes and/or cytosol. The biological function, if any, of *pgk* gene duplications and the consequent presence of several, sometimes highly diverse PGK isoenzymes in these parasites remains poorly understood. This protein has probably more relevant physiological functions than has been attributed to it so far. As has been documented in other organisms, it is very likely that several of these isoforms have additional important biological functions. However, it should be realized that for many of the PGK isoforms we described in this section about kinetoplastids, the currently available information is based on bioinformatic analysis of their genomes. In those cases, future proteomic analyses will be required to determine if expression of a (stable) protein occurs.

## 9. Phylogenetic analysis

**Figure 6.**
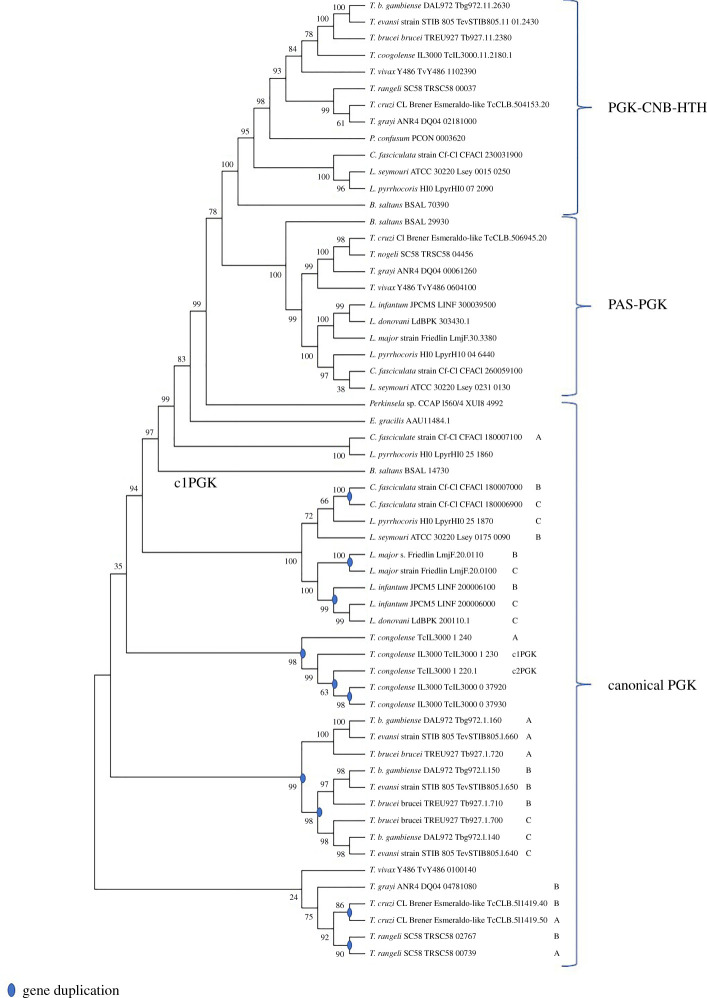
Kinetoplastid phylogenetic tree based on PGK sequences. PGK gene sequences were retrieved from the TriTrypDB, GeneDB and GenBank databases [94,123,360], and the predicted amino acid sequences aligned using Muscle (EMBL-EBI) [95]. Regions of uncertain alignment were omitted from the analysis leaving a total of 57 sequences (19 taxa) and 268 amino acid positions. The evolutionary history was inferred using the neighbour-joining (NJ) and the maximum parsimony (MP) methods [361], with similar results. The branches of the tree represent sequences, orthologous and paralogous, from different members of the Kinetoplastea phylum, with nodes depicted as filled blue ovals representing gene duplication events. Support for the nodes in the NJ and MP analyses was evaluated using the bootstrap procedure, and in each case, the bootstrap consensus tree inferred from 1000 replicates [362] was taken to represent the evolutionary history of the taxa analysed [362]. Branches corresponding to partitions reproduced in less than 50% bootstrap replicates are collapsed. The percentage of replicate trees in which the associated taxa clustered together in the bootstrap test (1000 replicates) are shown next to the branches [362]. For the NJ analysis, the evolutionary distances were computed using the p-distance method [363] and are given in the units of the number of amino acid differences per site. This analysis involved 58 amino acid sequences. All positions containing gaps and missing data were eliminated (complete deletion option). There were a total of 268 positions in the final dataset. For the MP analysis branches corresponding to partitions reproduced in less than 50% bootstrap replicates are collapsed. The percentages of replicate trees in which the associated taxa clustered together in the bootstrap test (1000 replicates) are shown next to the branches [361]. The MP tree was obtained using the subtree-pruning-regrafting (SPR) algorithm ([362], p. 126)] with search level 1 in which the initial trees were obtained by the random addition of sequences (10 replicates). This analysis involved 57 amino acid sequences. There were a total of 391 positions in the final dataset. Evolutionary analyses by NJ and MP were conducted in MEGA X [364].

We have performed a phylogenetic reconstruction of three groups of isoforms that are represented in different species and of which expression has been confirmed in at least some of them: the canonical PGKs, PAS–PGKs and PGK–CNB–HTHs. Figure 6 shows the consensus tree of relationships obtained by the neighbour-joining method for 268 amino acid positions of 57 sequences. The tree topology found for the taxa belonging to the orders Trypanosomatida, Eubodonida and Euglenozoa corresponds to what has been reported previously [293,365]. The phylogenetic analysis reveals that each paralogue of PGK forms a different cluster with the ‘canonical’ one, including the A, B and C isoenzymes. The PGKA and B sequences from stercorarian *Trypanosomas* are all grouped in a single clade. With respect to the salivarian *Trypanosomas* PGKs (A, B and C) sequences, these are grouped in two clades, one comprising all sequences from *T. b. brucei*, *T. b. gambiense* and *T. evansi*, and the second one grouping the PGK isoenzymes B and C from *T. congolense*. However, separately branching from these two clades is the cytosolic PGK sequence from *T. vivax*, which is within the stercorian clade, close to the PGKB from *T. grayi*. Another clade, subdividing itself in two clusters, comprises in one cluster all PGKB and C sequences from the different species of *Leishmania* (*L. major*, *L. donovani* and *L. infantum*), and in the second cluster, the PGKB and C sequences of their monoxenic trypanosomatid relatives *C. fasciculata* and *Leptomonas (L. pyrrhocoris* and *L. seymouri*). In independent branches are the other protistan sequences, the putative PGK (56 kDa) from *B. saltans*, the unique PGK (55 kDa) of *Perkinsela* sp. and the cytosolic PGK (45 kDa) from *E. gracilis*, these latter ones being most closely related to sequences from *L. pyrrhocoris* (LpyrH10_25_1860) and *C. fasciculata* (CFAC1_180007100), both of them with a molecular weight of 55 kDa like PGKA.

Two other major clusters are formed by the PGK–CNB–HTHs and PAS–PGKs from the kinetoplastids. The cluster of PAS–PGKs is subdivided into two main clades, one of them subdivided further in a group comprising sequences from *Leishmania* spp. and a second group with the PAS–PGKs from *C. fasciculata* and *Leptomonas*. The other cluster comprises all the sequences from stercorarian *Trypanosomas*. Although the PAS–PGK is completely absent in the salivarian *Trypanosomas*, the sequence TvY486_0604100 from *T. vivax* is in this cluster, because this gene product, although lacking a PAS domain, shares a high identity (approx. 60%) with the PGK domain of these sequences. Indeed, this *T. vivax* PGK gene presents synteny with PAS–PGK genes from all trypanosomatids that contain it (electronic supplementary material, table SII). By contrast, branching independently is the PAS–PGK from *B. saltans,* a member of the order Eubodonida. In the PGK–CNB–HTH cluster of *Trypanosomas*, all sequences from the salvarian trypanosomes are grouped in a single clade, which is separated from the clade of sequences from stercorarian trypanosomes. In a similar manner are grouped sequences from *C. fasciculata* and *Leptomonas* (*L. pyrrhocoris* and *L. seymouri*). In a separate branch is the PGK (PCON_0003620) from *P. confusum* which forms the most basal lineage of trypanosomatids, and the PGK of the free-living eubodonid *B. saltans*.

The phylogenetic tree also allows us to distinguish different types of homology, namely ‘paralogues’ (derived from gene duplication events) and ‘orthologues’ (from speciation events) [366]. Specifically, ‘canonical’ PGKs (A, B, C) of *T. brucei, T. b. gambiense* and *T. evansi* each inherited an orthologous gene ancestry, which then underwent one or more rounds of duplication and divergence to produce the different types of canonical PGKs in each descendent linage. With respect to the duplication event from which arose the paralogous PGKs A and B of *T. cruzi* and *T. rangeli*, these nodes are strongly supported by high maximum-likelihood bootstrap values (86% and 90%, respectively). The same behaviour was found for the rest of sequences from the Trypanosomatida order that are highlighted with a blue oval in figure 6.

## 10. What significance has *pkg* gene duplication?

Gene duplication is an important mechanism for acquiring new genes, and creating genome rearrangements and genetic variation [367]. It can take place through different processes that include retroposition, duplicative transposition, polyploidization, exchange between homologous chromosomes and exchange events within a single chromosome [367,368]. These mechanisms of gene acquisition have been studied in a wide variety of organisms, including protists, both with a free and parasitic lifestyle [369,370]. Gene expansion is considered to have been important for the evolution of adaptability of organisms to environmental variations and of their development to parasitism, for the latter especially involving those genes that contribute to a parasite's pathogenicity and resistance to host immunity [365,369–373]. The origin of the duplication of *pgk* genes in trypanosomatids and selective benefit of it for these organisms have as yet not been addressed.

In some organisms, such as the nematode *Strongyloides papillosus*, the evolution of parasitism is associated with a large expansion and chromosomal clustering of the CAP and Astacin gene families [374]. These two gene families experienced an extreme extent of duplication that coincided with the appearance of parasitism and were maintained during speciation events that ultimately resulted in different species of parasites that infect a wide variety of vertebrate hosts, including humans [375]. Both families of proteins, which are secreted by the parasites, fulfil important functions during infection, such as immunomodulation, tissue digestion and penetration [375,376]. In addition to being one of the most abundant gene groups in the genome, the *CAP* and *Astacin* genes are differentially expressed during the life cycle of these parasites [371].

On the other hand, studies with *Schistosoma mansoni* revealed that small-scale gene duplications (i.e. tandem, dispersed and proximal (TDP)) may be derived from retro-transposition and unequal crossing over. These genes derived by TDP duplications have undergone small divergence during evolution. An important aspect of this group of small-scale duplicated genes in *S. mansoni* is that they are part of some gene families associated with pathogenesis, involved in parasite survival during infection [372]. In *Fasciola hepatica*, something similar occurs. Although this organism has one of the largest genomes sequenced so far (1.3 Gb) [373], three times larger than that of *S. mansoni* (363 Mb) [377], its ability to infect and survive in different tissue environments (as it migrates from the intestine, through the liver and into the bile ducts) is mainly based on products of duplicated genes [373,378,379]. The genes of the tubulin, proteases and proteins involved in the glycosylation have undergone a marked process of duplication and are those that in turn exhibit an appreciable change in their expression during the life cycle [373,378]. Although gene duplication seems to be a key process for adaptation to the parasitic lifestyle of helminths, we can observe that different evolutionary solutions have been developed between different taxa that involve the duplication of different gene families.

The capacity for gene innovation introduced by duplications seems also to play an important role in apicomplexan protists. In these organisms, certain regions of the genome are enriched in multi-copy genes and some of these duplicated genes are species-specific. This specificity could indicate a role for duplication in adaptation and genetic innovation. Additionally, many of these recently duplicated genes are arranged in tandem. A distinctive feature of these tandemly arranged duplicated genes that are apicomplexan species specific is their distribution along the chromosome; they are located mainly in subtelomeric regions. Until now, the reason for the susceptibility of these regions to gene duplications is unknown. But it is clear that the duplications of these genes (which code for proteases, protein and DNA interacting proteins, etc.) contribute to the pathogenicity and resistance to host immunity of these organisms [380].

In *E. histolytica*, genes involved in lysis of host tissue and nutrient capture from the extracellular environment have undergone extensive gene duplications [381]. Furthermore, in *G. lamblia*, 40% of its genes have been identified as being duplicated and many of these genes are organized in tandem. Most of these duplicated genes encode variant-specific surface proteins (VSPs), ankyrin (Ank), and ADG (ancient duplicated genes) PKs. VSP proteins are essential for the successful parasitic life of *Giardia* in its hosts [382]. The Ank protein is a cytoskeleton component and is involved in cell cycle regulation and signal transduction [383] and ADG PKs are implicated in vesicular trafficking.

In the halophilic stramenopile protist *Halocafeteria seosinensis*, adaptation to halophilic habitats apparently depends on the acquisition of new genes through duplication. A large part of these duplicated genes (43%) are differentially expressed in response to the presence of salt in the environment [384]. In the ciliate *Paramecium tetraurelia*, most of the nearly 40 000 genes arose through at least three successive whole-genome duplications and many of these duplication processes coincided with speciation events. In addition, most of these genes (51%) are present in two copies, both highly expressed. However, duplication has apparently remained limited to gene dosing, rather than contributing to functional innovation [385].

In kinetoplastids, studies conducted with *B. saltans* revealed that its genome has high redundancy, with many tandemly duplicated genes; 34.7% of its genes are present in more than one copy. Some duplicate genes have an extremely high copy number, such as those of the GP46-like surface antigen gene family, of which 391 copies are present in the genome [282]. Some authors proposed that in the case of some asexual organisms (a characteristic attributed to *B. saltans*), the presence of multiple identical gene copies can help to counteract Muller's ratchet phenomenon. This is a process in which genomes of an asexual population accumulate harmful mutations irreversibly. However, the only way these organisms would be able to cope with it is by having multiple copies of the gene in question [386]. In parasitic kinetoplastids, gene duplications could have this and additional functions. Trypanosomatids are characterized by having a marked genomic plasticity that offers great ability to remodel or induce changes in their genome in response to environmental changes. These changes in the genome can occur at the overall chromosomal level (altered number (aneuploidy) and size) or in specific parts of individual chromosomes (gene amplification or deletion) [314,369,387,388]. Analyses of gene amplification/duplication in these organisms indicate that gene duplication is a potential source of genetic diversity and an important evolutionary factor that directly affects fitness [5,282,387,389–391]. Through this mechanism, there is an increase in the variability and alteration of gene expression in response to environmental conditions encountered in the host or by stressor agents like drugs [387]. The massive duplication of some genes would not only promote the creation of paralogous genes, but their formation would also have different consequences such as: (i) the neofunctionalization of duplicated genes; (ii) segregation of the original gene function between duplicates (subfunctionalisation); (iii) a change in the expression of the duplicated genes, in the case of parasites, for example, during the different stages of life cycle development, mediated by possible changes in their regulatory regions; and (iv) loss of function (pseudogenisation, or non-functionalization) [280]. Many of the genes that have undergone significant amplification are related to the parasite–host interaction. So appear the products of these amplified genes to be related to the process of cell adhesion/invasion, immune evasion, cytokinesis and parasite survival. Some of these duplicate gene products in trypanosomatids include surface proteins, peptidases, proteins involved in glycosylation, calpain-like cysteine protease, adenylyl cyclases and Ras-like proteins [369,387,388,392,393]. Another feature of these tandemly arranged duplicate genes is their location. In *T. cruz*i and *Leishmania* spp., these genes are all distributed internally in chromosomes and subtelomeric regions, while in *T. brucei*, several of these multigene families' clusters are enriched in subtelomeric regions [289–291]. In eukaryotes, the subtelomeric regions are preferential sites for the evolution of multigene families. However, little is known about the mechanisms involved in this process [394]. A distinctive feature of tandem duplicated genes is that many of them are pseudogenes. In the case of *T. brucei*, variant surface glycoproteins (VSGs) are involved in antigenic variation of this parasite. The *T. b. brucei* genome has approximately 800 different VSG genes, of which 18% are gene fragments and 66% full-length pseudogenes [289,291]. Some studies of these VSG pseudogenes have led to the suggestion that they can be used to generate expressed mosaic genes by ectopic recombination, and this recombination in turn allows the generation of new VSG variants [289,394]. The massive duplication into multigenic families in trypanosomatids can constitute evidence of the specialization that these parasites have to undertake to survive, as they must deal with different stressors within their hosts. *T. cruzi* and *Leishmania* spp. use this arsenal of proteins to invade and survive within the host cell, while in *T. brucei*, the multigene families are involved in antigenic variation to evade the humoral immune response and survive in the host bloodstream [387].

In the case of the *pgk* multigenic family, studies have so far mainly been performed from biochemical and structural points of views; the presence of several PGK isoenzymes is attributed to their role in the carbohydrate metabolism of these parasites. Little is known about the significance or reason for the existence of multiple, distinct PGK isoforms and the mechanism and selective force that have led to their preservation. However, the presence of various PGKs in kinetoplastids can have different physiological ‘raisons d’être’ that are closely related to each other and to the appearance of parasitism. The differentiation and retention of these isoforms may serve different purposes and be involved in different cellular processes.

### 10.1. Back-up function

Based on studies with *Saccharomyces cerevisiae*, it has been postulated that duplication and conservation of genes for metabolic enzymes are a mechanism for creating genetic robustness by ‘genetic buffering’ and functional complementation from duplicate genes [395,396]. This ‘genetic buffering’ through redundant gene networks would include alternative metabolic or regulatory or signal pathways, while complementation would be a way by which deletions in genes will have no significant effect on the phenotype [395,397]. This connection between robustness and redundancy is known as a back-up function. In the case of genetic robustness, functional compensation by duplicates might be a product of functional divergence after gene duplication. Additionally, a duplicate can be recruited into a novel gene network (or other types of functional divergence), setting up a boundary to stop its further divergence. In addition to providing robustness, gene duplication implies a continuous creation of new genes during evolution and increased complexity of molecular pathways [395]. Back-up of important or essential functions with duplicate genes plays an important role in genetic robustness, since individual duplicates would compensate for genetic dysfunctions. However, some members of a family of duplicates can acquire a specialized function, restricted expression or localization pattern that precludes functional complementation [396].

Different PGK isoforms could provide the kinetoplastid parasites with environmental robustness, since a role in the adaptation to environmental variations has been attributed to gene duplications [398]. The presence of multiple isoenzymes could allow organisms to survive through a wide range of environmental conditions without significantly altering their fitness, while they can increase the phenotypic robustness of the metabolic system [399,400]. For example, in *Drosophila melanogaster*, the presence of casein kinase II (CKII) isoforms increases phenotypic robustness and provides a flexible and parsimonious mechanism for adaptation to environmental fluctuations [400].

In *T. gondii*, metabolic plasticity is considered a crucial aspect for its successful parasitism in various host organisms and cell types. *T. gondii* tachyzoites primarily use glucose as energy source and a wide variety of anabolic precursors via glycolysis, the phosphate-pentose pathway and mitochondrial processes. They can also metabolize amino acids such as glutamine, after switching to the use of amino acids as a primary carbon and energy source when the host glucose is limiting [251]. Although *T. gondii* shows this preference for glucose, tachyzoites constitutively express also some enzymes involved in gluconeogenesis, including two isoforms of fructose-1,6-bisphosphatase (FBPase), which catalyse the dephosphorylation of fructose 1,6-bisphosphate (FBP) to fructose 6-phosphate [401]. These TgFBP1 and TgFBP2 isoenzymes are both located in the cytosol. They differ in their Vmax, Km for FBP and sensitivity to AMP. TgFBP2 exhibited a higher specific activity, lower substrate affinity and a markedly lower susceptibility to AMP inhibition. TgFBP1 only has minimal demonstrable FBPase activity *in vivo* in tachyzoites. Knockdown studies have shown that TgFBP2 is essential for growth under glucose-limiting and glucose-replete conditions and for acute virulence in mice. Rescue of the deficiency was possible with an active FBPase, but not by overexpression of TgFBP1, and was associated with global changes in central carbon metabolism and glycosylation pathways. The constitutive expression of TgFBP2, also in the presence of glucose, may have as function to increase the capacity of these parasites to rapidly adapt to changing nutrient conditions in their host cells and thus expand their host cell range [402].

As with the TgFBP isoenzymes, tachyzoites also express two catalytically active PYK enzymes (PYK1 and PYK2) with distinct subcellular location and enzymatic properties [115,403]. PYK1 is in the cytosol, while PYK2 is found mainly within the apicoplast and mitochondrion [403,404]. The cytosolic PYK1 works in canonical glycolysis to convert PEP to pyruvate, which is then transported to the mitochondrion where it enters the Krebs cycle [405]. By contrast, PYK2 catalyses pyruvate formation in the apicoplast which in turn provides substrates for the synthesis of isoprenoids and fatty acids. PYK1 is necessary for parasite growth, while PYK2 is available for the lytic cycle. The absence of both PYK isoenzymes abrogates tachyzoite growth. Co-expression of the enzymes contributes to robust regulation of central carbon metabolism.

Something similar is likely to occur with the PGK isoenzymes of *T. cruzi*. These isoenzymes would allow the use of different carbon sources. Results from biochemical studies on *T. cruzi* suggested that PGKA and B may act in the glycolytic direction, while PGKC does so in the gluconeogenic one [25]. At least in the case of *T. cruzi*, the presence of three active PGK isoenzymes (PGKA, PGKC and PAS-PGK) within glycosomes, but with different affinities for the substrates, could suggest a mechanism for reducing the effects of variations in the environment, thus increasing cellular robustness. The differences in the kinetic characteristics of the cytosolic and glycosomal PGK isoenzymes provide the parasite with greater metabolic buffer capacity. Affinity differences among isoforms will increase the flexibility in compensating larger changes in the environment [400]. The expression pattern of TgFBP2 in *T. gondii* is reminiscent of the expression pattern of the PGKC in *T. cruzi*. Although this latter isoenzyme is constitutively expressed during all phases of parasite growth, its expression is increased upon entering the stationary phase, when glucose availability becomes low. The pattern of expression and negative regulation by ATP that have been reported for the *T. cruzi* PGKC [124] suggests that this isoenzyme would have a similar function as TgFBP2 (i.e. to sustain an important gluconeogenic flux under glucose-starvation conditions). As for the PAS–PGK of *T. cruzi*, it is, with the present knowledge, difficult to attribute a specific function to its PAS domain. It could serve as a receptor for sensing nutritional changes (such as levels of glucose or other carbon sources in the different cell types where the parasite resides as amastigote, such as heart cells or muscle cells, or extracellularly as trypomastigote) that would modulate the fluxes through the glycolytic and/or gluconeogenic pathways in the parasite. The expression of PGK isoenzymes with different kinetics and mechanism of regulation as the PGKC may then enable rapid fine-tuning of the parasite's carbon metabolism over a wide range of nutrient conditions in the host and possibly contribute to the ability of these parasites to robustly proliferate within many different host cell compartments.

This idea could be supported by findings obtained through theoretical studies that provide evidence that the evolution of organisms in fluctuating environments leads to the development of robustness in metabolic networks, while robustness is lower in organisms that live in stable environments [406]. This could be an explanation for the difference in the number of PGK isoenzymes between the endosymbiotic *Perkinsela* sp. and other kinetoplastids with complex, parasitic life cycles such as *T. brucei*, *T. cruzi* and *Leishmania* spp. (table 3). Additionally, robustness of glucose metabolism may also be necessary for *P. falciparum*; the rapid growth and proliferation of the blood stage of this apicomplexan parasite is supported by robust metabolism of glucose as a source of carbon and energy via glycolysis [407]. Glycolysis would not only be necessary to meet the physiological demand of ATP, but also to support other physiological needs, because glycolytic intermediates are also directed to anabolic reactions. Similarly, in *T. cruzi*, the glycolytic enzymes in glycosomes and cytosol are interwoven in an elaborate metabolic network of catabolic and anabolic processes [340].

In other organisms such as plants of the genus *Alloteropsis*, gene dosage is associated with the emergence and establishment of the C_4_ carbon fixation pathway, with subsequent neofunctionalization [408]. C_4_ metabolism is strongly favoured by selection only under specific environmental conditions, such as drought, high temperatures and intense light. Different models suggested that a weak C_4_ cycle can emerge from enzymes that have not been adapted to the C_4_ catalytic context; gene duplications increasing the transcript abundance of C_4_-related genes in plants with a weak C_4_ cycle would then subsequently increase the strength of the pathway, which is predicted to boost carbon assimilation and fitness [409]. In other plants, such as *Arabidopsis thaliana*, duplicate genes are also associated with an increase in metabolic flux, suggesting that increased gene dosage results in increased metabolic activity [410]. Studies of *Leishmania* have shown duplication and differential expression of a large variety of genes, such as those for several transporters (including an inosine/guanosine transporter, two putative nucleoside transporters, two amino acid transporters, a glucose transporter and a pteridine transporter), proteins associated with drug resistance such as proteins involved in redox pathways, trypanothione synthetase and some hypothetical proteins [411,412]. Gene dosing is an adaptive trait that confers phenotypic plasticity in natural populations of *Leishmania* by rapidly regulating up or down the expression of transporter proteins to cope with the effects of environmental stress [411].

The duplication of *pkg* genes in *T. cruzi* may be related to gene dosage, since the creation of the glycosomal PGKA, although having relatively low catalytic activity [25], could have had the function of contributing to ATP generation inside the glycosomes and at the same time providing a mechanism for adaptation to environments with abundant glucose such as the bloodstream. The presence of a PGKA may even have become an adaptive advantage for the epimastigote form of *T. cruzi* (experiencing low glucose availability in the insect), by providing the parasite with a tool to be prepared for entering the mammalian bloodstream. PGKA could act within an adaptation mechanism to changes in glucose availability, somewhat similar to the role exerted by some glycolytic isoenzymes in yeasts [413]. The difference in location of the trypanosomatid PGK isoenzymes could have been the result of subcellular relocation upon the duplication process. In *S. cerevisiae*, too, proteins involved in catabolic processes (such as glycolysis) frequently developed new compartments (‘neolocalization’) or sublocalization patterns after duplication [414]. In the case of the PGK of *T. cruzi*, after duplication, a process of simultaneous neo- and sublocalization of the product of the *pgkA* gene has probably occurred. Additionally, the PGKA evolved new functional roles through changes of its kinetic properties, expression levels and probably, its interaction partners. The unique insert of 80 residues found in the N-terminal domain of PGKA of different kinetoplastids (salivarian and stercorian trypanosomes, *Crithidia* and *Leptomonas*) could be relevant for this latter property. This insert must have been acquired in a duplicated gene in a common ancestor. An analysis of these PGKAs, using different server predictors for segments of structural disorder, revealed several of such segments within the insert (W. Q. *et al*. 2020, unpublished results). Elements with a disordered structure within a protein are often involved in interactions with other proteins. In the case of *T. cruzi* PGKA, the insert may be responsible for the observed association of this isoenzyme with the glycosomal membrane [124], through electrostatic interactions of amino acids in the disordered segments with the membrane phospholipid head groups or with membrane proteins. Alternatively, PGKA may interact with other glycosomal proteins, for example, to channel its product (3PGA or 1,3BPGA), separate from the metabolite flux within the main glycolytic/gluconeogenic pathway complexes, to an enzyme that depends on it. In this respect, the finding of a putative glycerate kinase within the *T. cruzi* glycosomal proteome is suggestive [104]. However, this protein has not been found in the *T. brucei* glycosomal proteome, nor its gene identified in the genome databases of other trypanosomatids containing a PGKA. The lower level of conservation of the primary structure of the PGKA insert than the remainder of the enzyme (see §8.2.3.3.1) and the variation in the positions of the elements of disordered structure in the insert could indicate that the enzyme may be involved in different interactions in the other trypanosomatids. Future research is necessary to reveal the function(s) of this peculiar enzyme.

Finally, the ‘PGKL’ is another trypanosomatid protein that resulted from a duplication of a *pgk* gene, followed by recombination with other genes to produce a gene encoding a multidomain protein, PGK-CNB-HTH, and with between 1 and 4 predicted transmembrane segments at its C-terminal side. However, our analysis, and that by [60], predicted that the PGK is not active; it is considered a ‘dead’ enzyme. Also the HTH domain has evolved such that it is doubtful whether it retained DNA-binding capacity. However, and intriguingly, the protein is present in many trypanosomatids, thus must already have been present in the common ancestor, and has been relatively well conserved with an identity over 50%. Moreover, it has been shown to be expressed in several of these organisms. In *T. brucei*, the protein has been located in the flagellum, but from the sequences is predicted that it may be located elsewhere in other organisms. Together, these data suggest that the protein plays a role in the biology of these organisms, but its function remains enigmatic. In addition, genes encoding related proteins, but in which a potential HTH domain sequence could not be recognized, are also found in many kinetoplastids (table 3; electronic supplementary material, table SI). Whether they have been derived from *pgkL* genes or evolved independently is currently unknown.

In humans, the presence of duplicate genes contributes to attenuate the phenotypic consequences of deleterious human mutations, representing functional support [415]. Most duplicated genes are associated with metabolic and cell physiological processes, such as catabolism, anabolism and responses to stimuli [415]. Alterations in the gene copy number of metabolic enzymes are an omnipresent characteristic in all human cancers, and which also appear to have an impact on clinical aggressiveness and responsiveness to drugs [416].

### 10.2.

The neo- and/or subfunctionalization of PGKs could allow the adaptation of these protists to the different environmental changes to which they are exposed during their life cycle. The infection of alternative hosts during the life cycle could probably have been the selective force for the evolution of PGK isoforms, while gene duplication could have provided the gross genetic material. This proposition provides support for an alternative to Ohno's hypothesis. A *pgk* gene encodes a protein product that fulfilled a catalytic function; however, after the duplication process, the two child genes were able to specialize for additional functions to the specific one. This may have happened with the products of the *pgkA*/*B*/*C* gene array. These three isoenzymes have been shown to possess the ability to catalyse the reaction within the glycolytic and/or gluconeogenic pathway in *T. cruzi* [25,124]. In turn, another possibility is that the division of the functions of the ancestral *pgk* gene can have led to a neofunctionalization process. In this process, a series of changes in the coding region of a child gene (substitutions of non-synonymous nucleotides, addition of coding sequences for a domain and/or sorting signal for a subcellular compartment) that adapted its product to a new specialized function could have been selectively advantageous and this would then have allowed the fixation of the gene [417,418]. It is further possible that the appearance of one or more additional domains in a PGK isoenzyme has provided an ancestral trypanosomatid with an enzyme, the PAS–PGK, with new and specialized functions within the metabolism. Functions such as adaptation to environmental changes (such as internal oxygen sensor and carbon sources) and phosphorylation of glycosomal proteins have been suggested as possibilities for the PAS-PGK of *T. cruzi* [4]. It is also likely to have functions similar to those reported for *Leishmania*, where it has been shown that deletion of the PAS–PGK gene results in a 15% decreased glycolytic flux in promastigotes, as measured by lactate production, as well as an increase in the number of autophagosomes and an increased cell death [28]. Gene innovation introduced by duplication seems thus to play an important role in these parasites, similar to what has been proposed for other parasitic protists [380].

## 11. Conclusion and possible trends of future research

PGK is an enzyme that is distributed and highly conserved in Archaea, Bacteria and eukaryotes. Its main function is related to carbohydrate metabolism, specifically in glycolysis and gluconeogenesis, but the enzyme can also participate in a wide variety of other biological processes such as cell invasion, angiogenesis and tumour growth, flagellar movement, viral replication, induction of autophagy and cell death, thus fulfilling additional functions not related to its metabolic one. In many eukaryotic organisms, different PGK isoenzymes have been identified. The physiological significance of *pgk* gene duplications and the presence of different PGK isoenzymes are poorly understood. Our analyses of the genomes of most kinetoplastid parasites showed that *pgk* genes are often organized as tandem arrays and concentrated in specific chromosomes. Other thus far unreported *pgk* genes and *pseudogenes* were identified in some kinetoplastids. Several of these additionally identified *pgk* genes code for multidomain PGK enzymes and others for ‘dead’ PGK enzymes. So far, most studies of kinetoplastid PGK isoenzymes have focused almost exclusively on their biochemical and structural characterization. For that reason, we set out to address a little more the possible physiological significance of the presence of multiple PGKs in kinetoplastids and other protists. Due to the diversity of functions that PGK can fulfil, different evolutionary scenarios and functions are proposed to understand the physiological significance of PGK isoforms in kinetoplastids. Specifically, in the case of *T. cruzi*, a function beyond glycolysis/gluconeogenesis is proposed for some of the non-canonical PGKs. Providing metabolic plasticity is considered one of the main functions of the presence of isoenzymes. This plasticity would be crucial for adaptation to different environmental conditions and successful parasitism of this kinetoplastid in the extracellular and intracellular niches of its different hosts and the various host cell types that it parasitizes. Future molecular and biochemical studies of PGK isoforms of which the genes were identified in this study could give a clearer view of their physiological importance in *T. cruzi,* and by inference maybe also in related human parasites. Additionally, this analysis could also shed light on the possibility that some isoforms could serve for explorative studies for their use as drug targets in these parasites.

## Supplementary Material

Supplementary Information

## Data accessibility

Protein structures: All PGK structures available in the Protein Data Bank are listed with their accession codes in table 1. DNA sequences: The accession codes of all PGK sequences analysed in this paper and the databases from which they were retrieved are listed in table 3 and electronic supplementary material, tables SI, SII and SIII.
