# Supplementary material for: Phosphoglycerate kinase: structural aspects and functions, with special emphasis on the enzyme from Kinetoplastea
Source: Open Biol. 2020 Nov 25;10(11):200302. doi: 10.1098/rsob.200302 (PMC7729029; doi:10.1098/rsob.200302)
Supplement: Supplementary Information [file rsob200302supp1.docx]

**Royal Society Open Biology**

DOI: 10.1098/rsob.2016

**SUPPLEMENTARY MATERIAL**

**Phosphoglycerate kinase: structural aspects and functions, with special emphasis on the enzyme from Kinetoplastea**

Maura Rojas-Pirela^a^, Diego Andrade-Alviárez^b^, Verónica Rojas^a^, Ulrike Kemmerling^c^, Ana J. Cáceres^b^, Paul A. Michels^d^, Juan Luis Concepción^b^, Wilfredo Quiñones^b*^

^a^ Instituto de Biología, Facultad de Ciencias, Pontificia Universidad Católica de Valparaiso, Valparaiso 2373223, Chile

^b^ Laboratorio de Enzimología de Parásitos, Departamento de Biología, Facultad de Ciencias, Universidad de Los Andes, Mérida 5101, Venezuela

^c^ Instituto de Ciencias Biomédicas, Universidad de Chile, Facultad de Medicina, Santiago de Chile 8380453, Santigo de Chile

^d^ Centre for Immunity, Infection and Evolution and Centre for Translational and Chemical Biology, School of Biological Sciences, The University of Edinburgh, The King's Buildings, Edinburgh EH9 3FL, United Kingdom

^*^ Corresponding author. Laboratorio de Enzimología de Parásitos. Facultad de Ciencias. Universidad de Los Andes. La Hechicera, 5101-Mérida, Venezuela. Tel.: +58 274 2401302. Fax: +58 274 2401291. *E-mail address*: wilqui@ula.ve

| **Table SI.**  **Phosphoglycerate kinase (PGK) enzymes and PGK-related proteins identified by genomic analyses in different protists.** | | | | | | | | | | |
| --- | --- | --- | --- | --- | --- | --- | --- | --- | --- | --- |
| **Organism** | **Number of *pgk* genes** | **ID gene** | **Name** | **PM (kDa)** | **Associated domains** | **pI** | **Protein Length**  **a.a** | **Localization** | | **Database** |
| **Salivarian trypanosomas** | | | | | | | | | | |
| *T. brucei gambiense*  *DAL972* | 4 | Tbg972.1.150  Tbg972.1.140  Tbg972.1.160  Tbg972.11.2630 | Phosphoglycerate kinase, putative  Phosphoglycerate, putative  Phosphoglycerate kinase, putative  Phosphoglycerate kinase, putative | 45  47  56  101 | 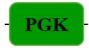  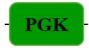  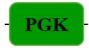  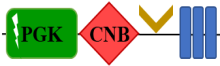 | 7.55  9.69  9.00  8.36 | 420  440  509  913 | Cytosol ◊  Cytosol ◊  Cytosol / Mitoch  Glycosome (Memb)* | | TriTrypDB [1] |
| **Stercorarian trypanosomes** | | | | | | | | | | |
| *T. cruzi*  *CL-Brener Non-Esmeraldo-Like* | 3 | TcCLB.505999.90  TcCLB.505999.100  TcCLB.506835.70 | Phosphoglycerate kinase, putative  3-Phosphoglycerate kinase, glycosomal (PGKA)  PAS-domain containing Phosphoglycerate kinase | 45  55  58 | 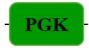  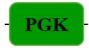  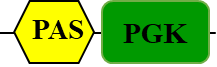 | 6.68  7.43  9.26 | 419  503  527 | Cytosol ◊  Glycosome ◊  Glycosome ◊ | | TriTrypDB [1] |
| *T. cruzi*  *DM28c2018* | 4 | C4B63_22G312  C4B63_22G311  C4B63_18g148  C4B63_2g568 | Phosphoglycerate kinase  3-Phosphoglycerate kinase glycosomal  PAS-domain containing Phosphoglycerate kinase  Phosphoglycerate Kinase | 45  55  58  100 | 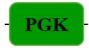  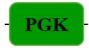  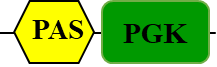  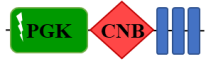 | 6.61  7.40  9.27  8.92 | 419  503  527  903 | Cytosol ◊  Gycosome ◊  Glycosome ◊  Glycosome (Memb)* | | TriTrypDB  [1] |
| *T. cruzi*  *Sylvio X10/1-202* | 6 | TCSYLVIO_000715  TCSYLVIO_007244  TCSYLVIO_009382  TCSYLVIO_005550  TCSYLVIO_005551  TCSYLVIO_003370 | 3-Phosphoglycerate kinase glycosomal  Phosphoglycerate kinase, putative  Phosphoglycerate kinase, putative  3-Phosphoglycerate kinase glycosomal  3-Phosphoglycerate kinase glycosomal  PAS-domain containing Phosphoglycerate kinase | 23  23  32  42  57  58 | 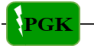  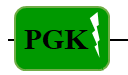  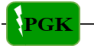  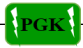  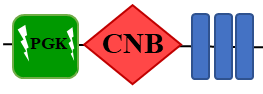  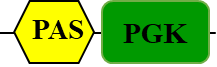 | 5.61  8.77  5.41  8.86  8.84  9.26 | 219  209  292  383  514  527 | Cytosol / Mitoch*  Cytosol *  Cytosol / Mitoch *  Cytosol *  Glycosome (Memb)*  Glycosome ◊ | | TriTrypDB [1] |
| *T. cruzi*  *TCC* | 6 | C3747_169g44  C3747_191g63  C3747_169G43  C3747_28g349  C3747_104g116  C3747_9G416 | Phosphoglycerate kinase  Phosphoglycerate kinase  3-Phosphoglycerate kinase glycosomal  PAS-domain containing Phosphoglycerate kinase  PAS-domain containing Phosphoglycerate kinase  Phosphoglycerate Kinase | 45  45  55  58  58  100 | 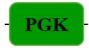  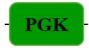  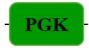  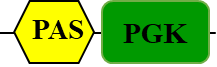  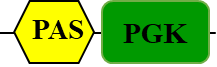  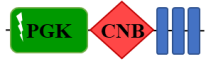 | 6.61  6.68  7.40  9.41  9.26  9.16 | 419  419  503  527  527  906 | Cytosol *  Cytosol *  Glycosome ◊  Glycosome ◊  Glycosome ◊  Glycosome (Memb)* | | TriTrypDB [1] |
| ***Leishmania*** | | | | | | | | | | |
| *Leishmania tropica*  *L590* | 6 | LTRL590_200006100  LTRL590_200006200  LTRL590_270023900  LTRL590_200006000  LTRL590_300036100 | Phosphoglycerate kinase, putative  Phosphoglycerate kinase, putative  phosphoglycerate kinase (pseudogene), putative  Phosphoglycerate kinase, putative  PAS-domain containing phosphoglycerate kinase, putative | 11  15  29  33  58 | 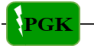  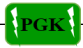  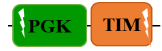  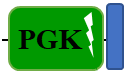  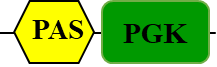 | 5.60  10.72  10.56  9.26  8.10 | 108  140  270  314  527 | Cytosol*  Cytoso/Mitoch*  Cytosol*  Cytosol*  Glycos/ Lys ◊ |  | |
| *L. infantum*  *JMP5* | 3 | LINF_200006100  LINF_200006000  LINF_300039500 | Phosphoglycerate kinase C-glycosomal (PGKC)  Phosphoglycerate kinase C-glycosomal (PGKC)  PAS-domain containing phosphoglycerate kinase, putative | 45  51  58 | 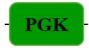  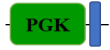  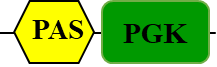 | 9.21  8.32  8.37 | 479  479  527 | Cytosol *  Glycosome*  Glycos/ Lys ◊ | TriTrypDB  [1] | |
| *L. donovani*  *BPK282A1* | 3 | LdBPK_200120.1  LdBPK_200110.1  LdBPK_303430.1 | Phosphoglycerate kinase B cytosolic (fragment)  Phosphoglycerate kinase C glycosomal (PGKC)  PAS-domain containing phosphoglycerate kinase, putative | 12  51  58 | 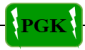  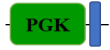  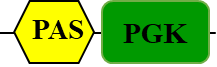 | 10.8  9.33  8.37 | 107  479  527 | Cytosol *  Glycosome ◊  Glycos/ Lys ◊ | TriTrypDB  [1] | |
| ***Leptomonas*** | | | | | | | | | | |
| *Leptomonas seymuri*  *ATCC 30220* | 3 | Lsey_0175_0090  Lsey_0015_0250  Lsey_0231_0130 | Phosphoglycerate kinase cytosolic  Phosphoglycerate kinase putative  PAS-domain containing phosphoglycerate kinase, putative | 52  102  58 | 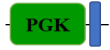  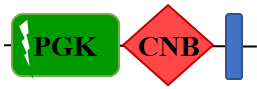  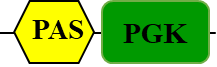 | 8.69  8.96  9.10 | 481  922  527 | Glycosome (Memb)*  Glycosome (Memb)*  Cytosol * | TriTrypDB  [1] | |

Sequences of different PGKs were retrieved from the the kinetoplastid Genomic Resource (TriTrypDB) [1]. Alignments and determination of identity percentages reported in the text were performed using Clustal Omega and Muscle (EMBL-EBI) [2]; query sequences used were Tb927.1.710, Tb927.1.700, Tb927.1.720, Tb927.11.2380, TcCLB.511419.40 and TcCLB.511419.50. Identification of domains and motifs present in PGK sequences was done using the following bioinformatics servers: PAS domain: Protein Blast (NCBI) [3], InterProScan [4], and Simple Modular Architecture Research Tool (SMART) [5]; e-values ranges between 1 e-6 and 8.9 e-9. CNB domain: NCBI, EMBL-EBI and SMART; e-values ranges between 2.6 e-6 and 4 e-36. HTH (Helix-Turn-Helix) motif: ExPaSy [6]; e-values ranges between -0.08 and 1.45. Predicting transmembrane protein topology with a hidden Markov model (TMHMM prediction) [7], and Phobius [8]; e-values ranges between 0.8 and 1. A specific e-value for each identified protein is given in Supplementary Table 3. The subcellular localization prediction of identified PGKs was based on either recognition of a PTS1 consensus sequence for glycosomal localization as described by Acosta et al. 2019 [9], or using the following servers: DeepLoc [10], WoLF PSORT [11], and Cell-Ploc [12].

Subcellular Localization Symbology: ***:** Predicted location through bioinformatics tools, **◊:** Established localization through experimental studies in a non-sequenced isolate, **▲:** Based on consensus sequence proposed in other studies [9]. Localization Symbology: Cytosol: Cytosol; Mitoch: Mitochondrion; Glycosome (memb); Glycosomal membrane; Lys: lysosome.

**Domain Symbology:** PGK domain
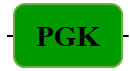
, PAS domain
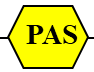
, Transmembrane domain
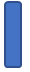
/
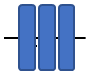
,PGK domain having lost residues involved in binding substrate (3PGA)
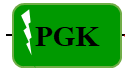
, PGK domain having lost residues involved in binding ATP
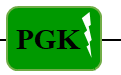
,PGK domain having lost residues involved in binding substrate (3PGA) and residues binding ATP
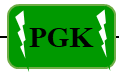
 , cyclic nucleotide-binding domain
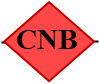
.

**Table SII. Synteny and organization of the phosphoglycerate kinase genes in the genome in some species of Kinetoplastea**

| **Organism** | **GeneID** | **Coding protein** | **Synteny** | **Upstream gene** | **Ups. Gene Name** | **Downstream gene** | **Down. gene Name** | **In which organisms?** |
| --- | --- | --- | --- | --- | --- | --- | --- | --- |
| **Free-living protists** | | | | | | | | |
| *B. saltans*  *strain Lake Konstanz* | BSAL_14730 | Phosphoglycerate kinase, putative | Yes | BSAL_14725 | Hypothetical protein, putative | BSAL_14735 | Hypothetical protein, putative | *T. brucei, T. cruzi, C. fasciculata, L. pyrrhocoris, T. rangeli, T. congolense, T. evansi,* |
|  | BSAL_29930 | Phosphoglycerate kinase, putative | Yes | BSAL_29925 | Hypothetical protein, putative | BSAL_29935 | Cellular apoptosis susceptibility-domain protein, putative | *C. fasciculata, T. grayi, L. major strain Friedlin, L. pyrrhocoris, P. confusum*, T. rangeli, T. cruzi, T. vivax* |
|  | BSAL_70390 | Phosphoglycerate kinase, putative | Yes | BSAL_70385 | Eukaryotic peptide chain release factor subunit 1, putative | BSAL_70395 | Hypothetical protein, putative | *C. fasciculata, T. grayi, L. pyrrhocoris, P. confusum, T. rangeli, T. cruzi* |
| **Endosymbiotic** | | | | | | | | |
| *Perkinsela* sp. | XU18_4992 | Phosphoglycerate kinase | --- | XU18_4991 | Peptidyl-tRNA hydrolase | XU18_4993 | Hypothetical protein | *---* |
| **Obligate parasites** | | | | | | | | |
| ***Paratrypanosoma*** | | | | | | | | |
| *P. confusum*  *CUL13* | PCON_0003620 | Phosphoglycerate kinase | Yes | PCON_0003610 | Hypothetical protein | PCON_0003630 | Unspecified product | *T. brucei, B. saltans, C. fasciculata, T. grayi, L. pyrrhocoris, T. rangeli, T. cruzi, T. congolense, T. evansi, T. vivax* |
| **Salivarian trypanosomes** | | | | | | | | |
| *T. brucei* TREU927 | Tb927.1.710 | PGKB | Yes | Tb927.1.700 | PGKC | Tb927.1.720 | PGKA | *T. cruzi, C. fasciculata, L. major strain Friedlin, L. pyrrhocoris, T. congolense, T. evansi, T. vivax.* |
|  | Tb927.1.700 | PGKC | Yes | Tb927.1.690 | Hypothetical protein, conserved | Tb927.1.710 | PGKB | *T. cruzi, C. fasciculata, T. grayi, L. major strain Friedlin, L. pyrrhocoris, T. congolense, T. evansi, T. vivax* |
|  | Tb927.1.720 | PGKA | Yes | Tb927.1.710 | PGKB | Tb927.1.730 | NADH-ubiquinone oxidorreductase complex I subunit | *T. cruzi, B. saltans, C. fasciculata, T. grayi. L. pyrrhocoris, T. congolense, T. evansi* |
|  | Tb927.11.2380 | Phosphoglycerate kinase, putative | Yes | Tb927.11.2370 | mRNA export factor MEX67 | Tb927.11.2390 | Tubulin-tyrosine ligase family, putative | *C. fasciculata, T. grayi, L. pyrrhocoris, P. confusum, T. rangeli, T. cruzi, T. congolense, T. evansi, T. vivax* |
| *T. congolense IL3000* | TcIL3000_1_220.1 | Phosphoglycerate kinase | Yes | TcIL3000_1_210 | Hypothetical protein, conserved | TcIL3000_1_230 | Phosphoglycerate kinase, putative | *T. brucei, T. cruzi, C. fasciculata, T. grayi, L. major strain Friedlin, L. pyrrhocoris, T. evansi, T. vivax.* |
|  | TcIL3000_1_230 | Phosphoglycerate kinase, putative | Yes | TcIL3000_1_220 | Phosphoglycerate kinase | TcIL3000_1_240 | Phosphoglycerate kinase, putative | *T. brucei, T. cruzi, C. fasciculata, L. major strain Friedlin, L. pyrrhocoris, T. evansi, T. vivax* |
|  | TcIL3000_1_240 | Phosphoglycerate kinase, putative | Yes | TcIL3000_1_230 | Phosphoglycerate kinase, putative | TcIL3000_1_250 | NADH-ubiquinone oxidorreductase complex I subunit | *T. brucei, T. cruzi, B. saltans, C. fasciculata, T. grayi, L. pyrrhocoris. T. rangeli, T. evansi* |
|  | TcIL3000.11.2180.1 | Phosphoglycerate kinase, putative (fragment) | Yes | TcIL3000.11.2170 | mRNA export factor MEX67 | --- | --- | *T. brucei, C. fasciculata, T. grayi, L. pyrrhocoris, P. confusum, T. rangeli, T. cruzi, T. evansi, T. vivax* |
| *T. evansi*  *STIB805* | TevSTIB805.1.640 | Phosphoglycerate kinase | Yes | TevSTIB805.1.630 | Hypothetical protein, conserved | TevSTIB805.1.650 | Phosphoglycerate kinase | *T. brucei, T. cruzi, C. fasciculata, T. grayi, L. major strain Friedlin, L. pyrrhocoris, T. congolense , T. vivax* |
|  | TevSTIB805.1.650 | Phosphoglycerate kinase | Yes | TevSTIB805.1.640 | Phosphoglycerate kinase | TevSTIB805.1.660 | Phosphoglycerate kinase | *T. brucei, T. cruzi, C. fasciculata, L. major strain Friedlin, L. pyrrhocoris, T. congolense, T. vivax* |
|  | TevSTIB805.1.660 | Phosphoglycerate kinase | Yes | TevSTIB805.1.650 | Phosphoglycerate kinase | TevSTIB805.1.670 | Hypothetical protein, conserved | *T. brucei, T. cruzi, B. saltans, C. fasciculata, T. grayi, L. pyrrhocoris, T. congolense.* |
|  | TevSTIB805.11_01.2430 | Phosphoglycerate kinase, putative | Yes | TevSTIB805.11_01.2420 | Hypothetical protein, conserved | TevSTIB805.11_01.2440 | Hypothetical protein, conserved | *T. brucei, C. fasciculata, T. grayi, L. pyrrhocoris, P. confusum, T. rangeli, T. cruzi, T. congolense, T. vivax* |
| *T. vivax*  *Y486* | TvY486_0604100 | Phosphoglycerate kinase, putative | Yes | TvY486_0604090 | Calmodulin, putative | TvY486_0604110 | CAS/CSE/importin domain protein, putative | *B. saltans, C. fasciculata, T. grayi, L. major strain Friedlin, L. pyrrhocoris, P. confusum*, T. rangeli, T. cruzi* |
|  | TvY486_0100140 | Phosphoglycerate kinase, putative | Yes | TvY486_0100130 | Hypothetical protein, conserved | TvY486_0100150 | Phosphoglycerate kinase, putative (fragment) | *T. brucei, T. cruzi, C. fasciculata, T. grayi, L. major strain Friedlin, L. pyrrhocoris, T. rangeli, T. congolense, T. evansi* |
|  | TvY486_1102390 | Phosphoglycerate kinase, putative | yes | TvY486_1102380 | Hypothetical protein | TvY486_1102400 | Flabarin-like protein | *T. brucei, C. fasciculata, T. grayi, L. pyrrhocoris, P. confusum, T. rangeli, T. cruzi, T. congolense, T. evansi* |
|  | TvY486_0100150 | Phosphoglycerate kinase, putative (fragment) | yes | TvY486_0100140 | Phosphoglycerate kinase, putative | TvY486_0100160 | NADH-ubiquinone oxidorreductase complex I subunit | *T. brucei, T. cruzi, C. fasciculata, T. grayi, L. major strain Friedlin, L. pyrrhocoris, T. congolense, T. evansi* |
| **Stercorarian and reptilian trypanosomes** | | | | | | | | |
| *T. cruzi*  CL Brener Esmeraldo-like | TcCLB.511419.40 | Phosphoglycerate kinase, putative | Yes | TcCLB.511419.30 | Hypothetical protein, conserved | TcCLB.511419.50 | 3-phosphoglycerate kinase. glycosomal | *T. brucei, C. fasciculata, T. grayi, L. major strain Friedlin, L. pyrrhocoris, T. rangeli, T. congolense, T. evansi, T. vivax* |
|  | TcCLB.511419.50 | 3-Phosphoglycerate kinase, glycosomal | Yes | TcCLB.511419.40 | Phosphoglycerate kinase, putative | TcCLB.511419.60 | NADH-ubiquinone oxidoreductase complex I subunit, putative | *T. brucei, B. saltans, C. fasciculata, T. grayi, L. pyrrhocoris, T. rangeli, T. congolense, T. evansi* |
|  | TcCLB.506945.20 | PAS-domain containing Phosphoglycerate kinase, putative | Yes | TcCLB.506945.10 | Hypothetical protein, conserved | TcCLB.506945.30 | CAS/CSE/importin domain protein, putative | *B. saltans, C. fasciculata, T. grayi, L. major strain Friedlin, L. pyrrhocoris, P. confusum,* T. rangeli, T. vivax* |
|  | TcCLB.504153.20 | Phosphoglycerate kinase, putative | Yes | TcCLB.504153.10 | Hypothetical protein, conserved | TcCLB.504153.30 | Flabarin, putative | *T. brucei, B. saltans, C. fasciculate, T. grayi, L. pyrrhocoris, P. confusum, T. rangeli, T. congolense, T. evansi, T. vivax* |
| *T. rangeli*  *SC58* | TRSC58_02767 | Phosphoglycerate kinase | Yes | --- | --- | --- | --- | *T. cruzi, T. grayi, L. pyrrhocoris, T. vivax* |
|  | TRSC58_00739 | Phosphoglycerate kinase | Yes | --- | --- | --- | --- | *T. cruzi, B. saltans, T. congolense* |
|  | TRSC58_04456 | PAS-domain containing Phosphoglycerate kinase | Yes | --- | --- | --- | --- | *B. saltans, C. fasciculata, T. grayi, L. major strain Friedlin, L. pyrrhocoris, P. confusum*, T. cruzi, T. vivax* |
|  | TRSC58_00037 | Phosphoglycerate kinase | Yes | --- | --- | --- | --- | *T. brucei, B. saltans, C. fasciculata,, T. grayi, L. pyrrhocoris, P. confusum, T. cruzi, T. congolense, T. evansi, T. vivax* |
| *T. grayi*  *ANR4* | DQ04_04781080 | Phosphoglycerate kinase | Yes | DQ04_04781070 | Hypothetical protein | --- | --- | *T. brucei, T. cruzi, L. pyrrhocoris, T. rangeli, T. congolense, T. evansi, T. vivax* |
|  | DQ04_00061260 | PAS-domain containing Phosphoglycerate kinase, putative | Yes | DQ04_00061250 | putative CAS/CSE/importin domain protein | DQ04_00061270 | Putative calmodulin | *B. saltans, C. fasciculata, L. major strain Friedlin, L. pyrrhocoris, P. confusum*, T. rangeli, T. cruzi, T. vivax* |
|  | DQ04_02181000 | Phosphoglycerate kinase | Yes | --- | --- | DQ04_02181010 | Nuclear RNA export factor 1/2 | *T. brucei, B. saltans, C. fasciculata, L. pyrrhocoris, P. confusum, T. rangeli, T. cruzi, T. congolense, T. evansi, T. vivax* |
|  | DQ04_13681000 | 3-Phosphoglycerate kinase, glycosomal | Yes | --- | --- | DQ04_13681010 | Hypothetical protein | *T. brucei, T. cruzi, C. fasciculata, L. pyrrhocoris, T. rangeli+, T. congolense, T. evansi* |
| ***Leishmania*** | | | | | | | | |
| *Leishmania major*  *strain Friedlin* | LmjF.20.0110 | Phosphoglycerate kinase B, cytosolic | Yes | LmjF.20.0100 | Phosphoglycerate kinase C, glycosomal | LmjF.20.0120 | NADH-ubiquinone oxidoreductase complex I subunit, putative | *T. brucei, T. cruzi, C. fasciculata, L. pyrrhocoris, T. congolense, T. evansi, T. vivax* |
|  | LmjF.20.0100 | Phosphoglycerate kinase C, glycosomal | Yes | LmjF.20.0090 | Ion channel/Calcium-activated BK potassium channel alpha subunit, putative | LmjF.20.0110 | Phosphoglycerate kinase B, cytosolic | *T. brucei, T. cruzi, C. fasciculata, L. pyrrhocoris, T. congolense, T. evansi, T. vivax* |
|  | LmjF.27.1720 | Phosphoglycerate kinase (pseudogene) putative | Yes | LmjF.27.1710 | Eukaryotic translation release factor, putative | LmjF.27.1730 | Flabarin, putative | *Only Leishmania species* |
|  | LmjF.30.3380 | PAS-domain containing Phosphoglycerate kinase, putative | Yes | LmjF.30.3370 | Hypothetical protein, conserved | LmjF.30.3390 | CAS/CSE/importin domain protein, putative | *B. saltans, C. fasciculata, T. grayi, L. pyrrhocoris, P. confusum*, T. rangeli, T. cruzi, T. vivax* |
| ***Leptomonas*** | | | | | | | | |
| *Leptomonas*  *pyrrhocoris*  *H10* | LpyrH10_25_1870 | Phosphoglycerate kinase C, glycosomal (PGKC), putative mitochondrial | Yes | LpyrH10_25_1860 | 3-Phosphoglycerate kinase, glycosomal, putative mitochondrial | LpyrH10_25_1880 | Hypothetical protein, conserved | *T. brucei, T. cruzi, C. fasciculata, T. grayi, L. major strain Friedlin, T. rangeli, T. congolense, T. evansi, T. vivax* |
|  | LpyrH10_25_1860 | 3-Phosphoglycerate kinase, glycosomal, putative mitochondrial | Yes | LpyrH10_25_1850 | Hypothetical protein, conserved, putative mitochondrial | LpyrH10_25_1870 | Phosphoglycerate kinase C, glycosomal (PGKC), putative mitochondrial | *T. brucei, T. cruzi, C. fasciculata, T. grayi, T. rangeli+, T. congolense, T. evansi* |
|  | LpyrH10_04_6440 | PAS-domain containing Phosphoglycerate kinase, putative, putative mitochondrial | Yes | LpyrH10_04_6430 | Hypothetical protein, conserved | LpyrH10_04_6450 | CAS/CSE/importin domain protein, putative | *B. saltans, C. fasciculata, T. grayi, L. major strain Friedlin, P. confusum*, T. rangeli, T. cruzi, T. vivax* |
|  | LpyrH10_07_2090 | Phosphoglycerate kinase, putative | Yes | LpyrH10_07_2080 | Eukaryotic translation release factor, putative | LpyrH10_07_2100 | Hypothetical protein, conserved, putative mitochondrial | *T. brucei, B. saltans, C. fasciculata, T. grayi, P. confusum, T. rangeli, T. cruzi, T. congolense, T. evansi, T. vivax* |
| ***Crithidia*** | | | | | | | | |
| *Crithidia*  *fasciculata*  *Cf-Cl* | CFAC1_180007000 | Phosphoglycerate kinase B, cytosolic | Yes | CFAC1_180006900 | Phosphoglycerate kinase B, cytosolic | CFAC1_180007100 | Phosphoglycerate kinase B, cytosolic | *T. brucei, T. cruzi, L. major strain Friedlin, L. pyrrhocoris, T. congolense, T. evansi, T. vivax* |
|  | CFAC1_180006900 | Phosphoglycerate kinase B, cytosolic | Yes | CFAC1_180006800 | Ion channel/Calcium-activated BK potassium channel alpha subunit, putative | CFAC1_180007000 | Phosphoglycerate kinase B, cytosolic | *T. brucei, T. cruzi, L. major strain Friedlin, L. pyrrhocoris, T. congolense, T. evansi, T. vivax* |
|  | CFAC1_180007100 | Phosphoglycerate kinase B, cytosolic | Yes | CFAC1_180007000 | Phosphoglycerate kinase B, cytosolic | CFAC1_180007200 | NADH-ubiquinone oxidoreductase complex I subunit, putative | *T. brucei, T. cruzi, B. saltans, T. grayi, L. pyrrhocoris, T. congolense, T. evansi* |
|  | CFAC1_260059100 | PAS-domain containing Phosphoglycerate kinase, putative | Yes | CFAC1_260059000 | Zinc finger C-x8-C-x5-C-x3-H type (and similar), putative | CFAC1_260059200 | CAS/CSE/importin domain protein, putativ | *B. saltans, T. grayi, L. major strain Friedlin, L. pyrrhocoris, T. rangeli P. confusum*, T. cruzi, T. vivax* |
|  | CFAC1_230031900 | Phosphoglycerate kinase/Cyclic nucleotide-binding domain containing protein, putative | Yes | CFAC1_230031800 | Hypothetical protein, conserved | CFAC1_230032000 | Eukaryotic translation release factor, putative | *T. brucei, B. saltans, T. grayi, L. pyrrhocoris, P. confusum, T. rangeli, T. cruzi, T. congolense, T. evansi, T. vivax* |

--- : Data not available

*: Unspecified product

+: Hypothetical protein

**Table SIII.** e-values obtained for similarities of regions of proteins described in this review with known, canonical protein domains listed above the columns of this table, when performing searches using the servers and software indicated underneath.

| **Organism** | | **Gene** | **e-value** | | | | | |
| --- | --- | --- | --- | --- | --- | --- | --- | --- |
|  |  | | **Domains described** | | | | | |
|  |  |  | **PGK** | **PAS** | **CNB** | **TM** | **HTH** | **PGK/TIM** |
| *Diplonema* | BAQ25443 | | 8.2e^-150^ |  |  |  |  |  |
| *Bodo saltans* | BSAL_14730  BSAL_29930  BSAL_70390 | | 5.5e^-155^  8.5e^-138^  5.95e^-56^ | 8.9e-9 | 5.61e^-8^ | 0.8-1 |  |  |
| *Perkinsela* sp. *CCA* | XU18_4992 | | 2.4e^-130^ |  |  |  |  | |
| *P. confusum* | PCON_0085140  PCON_0003620 | | 7.6e^-111^  7.9e^-87^ |  | 3.6e^-10^ | 0.8-1 |  |  |
| *T. brucei* | Tb927.1.710  Tb927.1.700  Tb927.1.720  Tb927.11.2380 | | 4.1e^-160^  4.2e^-161^  2.3e^-157^  2.0e^-104^ |  | 2.1e^-10^ | 0.8-1 | 1.45 |  |
| *T. congolense* | TcIL3000_1_220.1  TcIL3000_1_230  TcIL3000_1_240  TcIL3000.11.2180.1  TcIL3000_0_37920  TcIL3000_0_37930 | | 5.3e^-160^  2.1e^-161^  1.6e^-159^  2.1e^-98^  4.9e^-161^  5.3e^-160^ |  | 4e^-36^ |  | 29 (GYM) |  |
| *T. evansi* | TevSTIB805.1.640  TevSTIB805.1.650  TevSTIB805.1.660  TevSTIB805.11_01.2430 | | 2.3e^-161^  5.1e^-158^  2.3e^-157^  8.2e^-95^ |  | 3.3e^-9^ | 0.9-1 | 1.45 |  |
| *T. vivax* | TvY486_0604100  TvY486_0100140  TvY486_1102390  TvY486_0100150 | | 4.9e^-121^  6.0e^-159^  1.21e^-70^  2.7e^-106^ |  | 2.1e^-9^ | 0.9-1 |  |  |
| *T. cruzi* | TcCLB.506127.9  TcCLB.511419.40  TcCLB.511419.50  TcCLB.506945.20  TcCLB.504153.20 | | 2.7e^-9^  1.4e^-161^  6.9e^-157^  1.6e^-175^  2.4e^-101^ | 1.7e^-8^ | 3.8e^-7^ | 0.9-1 |  |  |
| *T. rangeli* | TRSC58_02767  TRSC58_00739  TRSC58_04456  TRSC58_00037 | | 1.2e^-159^  6.4e^-159^  1.14e^-154^  3.27e^-93^ | 4.8e^-8^ | 6.1e^-06^ | 1 | 0.87 |  |
| *T. grayi* | DQ04_04781080  DQ04_00061260  DQ04_02181000  DQ04_13681000 | | 3.3e^-160^  7.2e^-174^  1.3e^-97^  6.9e^-58^ | 3.8e^-7^ | 1.3e^-8^ |  | 1.14 |  |
| *Leishmania major* | LmjF.20.0110  LmjF.20.0100  LmjF.27.1720  LmjF.30.3380 | | 1.2e^-155^  3.8e^-158^  2.7e^-7^  2.3e^-163^ | 6.1e^-8^ |  | 1 |  | 1.4e^-5^ |
| *Leptomonas*  *pyrrhocoris* | LpyrH10_25_1870  LpyrH10_25_1860  LpyrH10_04_6440  LpyrH10_07_2090 | | 2.7e^-159^  4e^-139^  1.1e^-161^  2.07e-74 | 7.5e^-8^ | 1.7e^-10^ | 1 |  |  |
| *Crithidia*  *fasciculata* | CFAC1_180007000  CFAC1_180006900  CFAC1_180007100  CFAC1_260059100  CFAC1_230031900 | | 2.4e^-157^  4.0e^-157^  3.5e^-172^  9.1e^-173^  3.9e^-73^ | 6.5e^-7^ | 3.7e^-10^ | 0.9-1 | -0.08 |  |
| *Angomonas deanei* | AGDE_06248  AGDE_05604  AGDE_10081  AGDE_10148  AGDE_05595  AGDE_08002  AGDE_07220  AGDE_10178  AGDE_05077  AGDE_04297  AGDE_02770  AGDE_05968 | | 1.6e^-92^  6.0e^-136^  6.0e^-136^  6.0e^-136^  3.4e^-159^  5.3e^-135^  2.9e^-75^  4.9e^-171^  2.7e^-75^  2.9e^-75^  2.9e^-75^  1.0e^-25^ | 5.2e^-8^ | 1.3e^-8^  1.2e^-8^  1.3e^-8^  1.3e^-8^  1.3e^-8^ | 0.8-1  0.8-1  0.8-1  0.8-1  0.8-1 |  |  |
| *Strigomonas culicis* | STCU_09535  STCU_09108  STCU_07788  STCU_01151  STCU_05393  STCU_04883  STCU_04975  STCU_02304  STCU_08802  STCU_06143 | | 1.3e^-156^  1.5e^-160^  9.1e^-160^  1.7e^-160^  1.7e^-160^  2.0e^-152^  1.3e^-172^  7.6e^-76^  7.6e^-76^  7.6e^-76^ | 2.2e^-7^ | 7.7e^-11^  7.7e^-11^  7.7e^-11^ | 1  1  1  1  1  1 |  |  |
| *T. brucei gambiense* | Tbg972.1.150  Tbg972.1.140  Tbg972.1.160  Tbg972.11.2630 | | 2.1e^-160^  2.3e^-161^  2.3e^-157^  8.18e^-95^ |  | 3.2e^-9^ |  | 1.45 |  |
| *T. cruzi*  *CL-Brener Non-Esmeraldo-Like* | TcCLB.505999.90  TcCLB.505999.100  TcCLB.506835.70 | | 5.5e^-161^  2.3e^-159^  1.3e^-175^ | 1.7e^-8^ |  |  |  |  |
| *T. cruzi*  *DM28c2018* | C4B63_22G312  C4B63_22G311  C4B63_18g148  C4B63_2g568 | | 6.0e-^159^  3.5e^-157^  1.63e^-175^  3.88e^-100^ | 1.7e^-8^ | 2.7e^-6^ | 1 |  |  |
| *T. cruzi*  *Sylvio X10/1* | TCSYLVIO_000715  TCSYLVIO_007244  TCSYLVIO_009382  TCSYLVIO_005550  TCSYLVIO_005551  TCSYLVIO_003370 | | 7.4e^-127^  5.7e^-94^  3.7e^-172^  6.85e^-58^  5.3e^-21^  1.6e^-175^ | 1.7e^-8^ | 4.8e^-7^ |  |  |  |
| *T. cruzi*  *TCC* | C3747_169g44  C3747_191g63  C3747_169G43  C3747_28g349  C3747_104g116  C3747_9G416 | | 1.4e^-161^  5.5e^-161^  6.9e^-157^  6.95e^-176^  1.63e^-175^  5.65e^-102^ | 1.7e^-08^  1.7e^-08^  1.0e^-06^ | 1 |  |  |  |
| *L. infantum*  *JMP5* | LINF_200006100  LINF_200006000  LINF_300039500 | | 1.8e^-158^  2.9e^-158^  5.6e^-178^ | 6.4e^-8^ | 1 |  |  |  |
| *L. donovani*  *BPK282A1* | LdBPK_200120.1  LdBPK_200110.1  LdBPK_303430.1 | | 1.8e^-41^  2.4e-158  4.1e^-178^ | 6.8e^-8^ |  | 1 |  |  |
| *Leptomonas seymuri* | Lsey_0175_0090  Lsey_0015_0250  Lsey_0231_0130 | | 1.3e^-159^  6.4e^-75^  3.8e^-170^ | 1.6e^-8^ | 4.0e^-9^ | 1  0.9-1 |  |  |

*PAS domain*: Protein Blast (NCBI) [3], InterProScan [4], and Simple Modular Architecture Research Tool (SMART) [5]. CNB domain: NCBI, EMBL-EBI, SMART. HTH (Helix-Turn-Helix) motif: ExPaSy [6], GYM [13], and iDNA-Prot [14]. Membrane helix: SMART [5], Predicting transmembrane protein topology with a hidden Markov model (TMHMM prediction) [7], and Phobius [8].

**Supplementary references**

1. Aslett M *et al.* 2010 TriTrypDB: a functional genomic resource for the Trypanosomatidae. *Nucleic Acids Res.* **38**, D457-462. (doi:10.1093/nar/gkp851)

2. McWilliam H, Li W, Uludag M, Squizzato S, Park YM, Buso N, Cowley AP, Lopez R. 2013 Analysis Tool Web Services from the EMBL-EBI. *Nucleic Acids Res.* **41**, W597-600. (doi:10.1093/nar/gkt376)

3. Marchler-Bauer A *et al.* 2015 CDD: NCBI’s conserved domain database. *Nucleic Acids Res.* **43**, D222-226. (doi:10.1093/nar/gku1221)

4. Finn RD *et al.* 2017 InterPro in 2017-beyond protein family and domain annotations. *Nucleic Acids Res.* **45**, D190–D199. (doi:10.1093/nar/gkw1107)

5. Letunic I, Bork P. 2018 20 years of the SMART protein domain annotation resource. *Nucleic Acids Res.* **46**, D493–D496. (doi:10.1093/nar/gkx922)

6. Artimo P *et al.* 2012 ExPASy: SIB bioinformatics resource portal. *Nucleic Acids Res.* **40**, W597-603. (doi:10.1093/nar/gks400)

7. Krogh A, Larsson B, von Heijne G, Sonnhammer EL. 2001 Predicting transmembrane protein topology with a hidden Markov model: application to complete genomes. *J. Mol. Biol.* **305**, 567–580. (doi:10.1006/jmbi.2000.4315)

8. Käll L, Krogh A, Sonnhammer ELL. 2007 Advantages of combined transmembrane topology and signal peptide prediction--the Phobius web server. *Nucleic Acids Res.* **35**, W429-432. (doi:10.1093/nar/gkm256)

9. Acosta H, Burchmore R, Naula C, Gualdrón-López M, Quintero-Troconis E, Cáceres AJ, Michels PAM, Concepción JL, Quiñones W. 2019 Proteomic analysis of glycosomes from Trypanosoma cruzi epimastigotes. *Mol. Biochem. Parasitol.* **229**, 62–74. (doi:10.1016/j.molbiopara.2019.02.008)

10. Almagro Armenteros JJ, Sønderby CK, Sønderby SK, Nielsen H, Winther O. 2017 DeepLoc: prediction of protein subcellular localization using deep learning. *Bioinforma. Oxf. Engl.* **33**, 3387–3395. (doi:10.1093/bioinformatics/btx431)

11. Horton P, Park K-J, Obayashi T, Fujita N, Harada H, Adams-Collier CJ, Nakai K. 2007 WoLF PSORT: protein localization predictor. *Nucleic Acids Res.* **35**, W585-587. (doi:10.1093/nar/gkm259)

12. Chou K-C, Shen H-B. 2008 Cell-PLoc: a package of Web servers for predicting subcellular localization of proteins in various organisms. *Nat. Protoc.* **3**, 153–162. (doi:10.1038/nprot.2007.494)

13. Narasimhan G, Bu C, Gao Y, Wang X, Xu N, Mathee K. 2002 Mining protein sequences for motifs. *J. Comput. Biol. J. Comput. Mol. Cell Biol.* **9**, 707–720. (doi:10.1089/106652702761034145)

14. Lin W-Z, Fang J-A, Xiao X, Chou K-C. 2011 iDNA-Prot: identification of DNA binding proteins using random forest with grey model. *PloS One* **6**, e24756. (doi:10.1371/journal.pone.0024756)
